# The CRL5–SPSB3 ubiquitin ligase targets nuclear cGAS for degradation

**DOI:** 10.1038/s41586-024-07112-w

**Published:** 2024-02-28

**Authors:** Pengbiao Xu, Ying Liu, Chong Liu, Baptiste Guey, Lingyun Li, Pauline Melenec, Jonathan Ricci, Andrea Ablasser

**Affiliations:** 1 Global Health Institute, Swiss Federal Institute of Technology Lausanne (EPFL), Lausanne, Switzerland; 2 Institute for Cancer Research (ISREC), Swiss Federal Institute of Technology Lausanne (EPFL), Lausanne, Switzerland; 3 Present Address: School of Medicine, Jiangnan University, Wuxi, China

**Keywords:** Innate immunity, Proteolysis

## Abstract

Cyclic GMP-AMP synthase (cGAS) senses aberrant DNA during infection, cancer and inflammatory disease, and initiates potent innate immune responses through the synthesis of 2′3′-cyclic GMP-AMP (cGAMP)^1–7^. The indiscriminate activity of cGAS towards DNA demands tight regulatory mechanisms that are necessary to maintain cell and tissue homeostasis under normal conditions. Inside the cell nucleus, anchoring to nucleosomes and competition with chromatin architectural proteins jointly prohibit cGAS activation by genomic DNA^8–15^. However, the fate of nuclear cGAS and its role in cell physiology remains unclear. Here we show that the ubiquitin proteasomal system (UPS) degrades nuclear cGAS in cycling cells. We identify SPSB3 as the cGAS-targeting substrate receptor that associates with the cullin–RING ubiquitin ligase 5 (CRL5) complex to ligate ubiquitin onto nuclear cGAS. A cryo-electron microscopy structure of nucleosome-bound cGAS in a complex with SPSB3 reveals a highly conserved Asn-Asn (NN) minimal degron motif at the C terminus of cGAS that directs SPSB3 recruitment, ubiquitylation and cGAS protein stability. Interference with SPSB3-regulated nuclear cGAS degradation primes cells for type I interferon signalling, conferring heightened protection against infection by DNA viruses. Our research defines protein degradation as a determinant of cGAS regulation in the nucleus and provides structural insights into an element of cGAS that is amenable to therapeutic exploitation.

## Abstract (web summary)

The ubiquitin proteasomal system degrades nuclear cGAS in cycling cells.

## Main

**Fig. 1:**
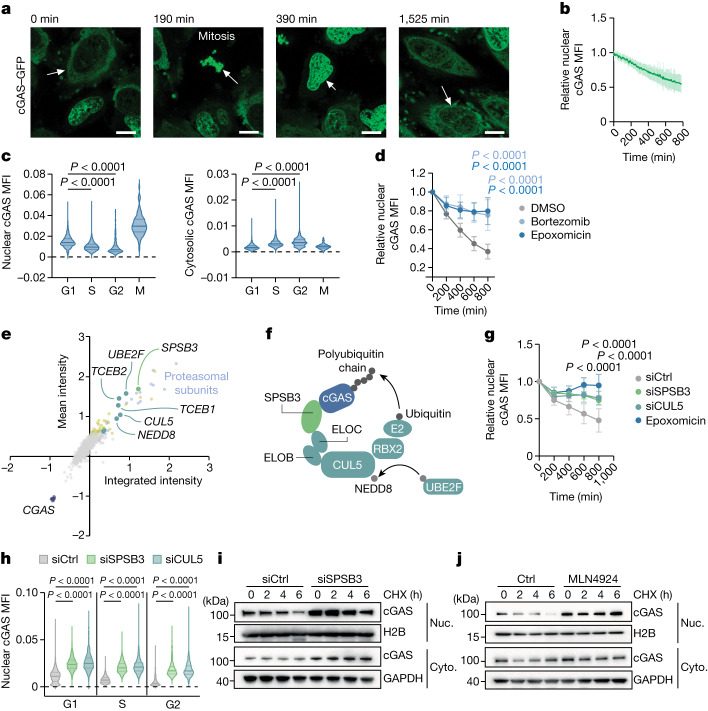
Identification of SPSB3 and CUL5 in the regulation of nuclear cGAS. **a**, Representative image sequence demonstrating cGAS chromosome attachment in mitosis followed by intranuclear redistribution and degradation in cGAS–GFP-expressing HeLa cells (Supplementary Video 1). Scale bars, 10 μm. **b**, The relative nuclear cGAS–GFP mean fluorescence intensity (MFI) in post-mitotic HeLa cells. *n* = 29. **c**, QIBC analysis of endogenous nuclear (left) and cytosolic (right) cGAS levels in HeLa cells. **d**, The relative nuclear cGAS–GFP MFI in post-mitotic HeLa cells that were treated with epoxomicin (*n* = 31), bortezomib (*n* = 30) or DMSO (*n* = 31). **e**, Focused RNAi-based screen of UPS factors regulating nuclear cGAS–GFP abundance. Each dot shows the cGAS–GFP mean intensity blotted against integrated intensity. Knockdowns of genes that yielded increased cGAS–GFP levels (>3 s.d.) relative to the control siRNA are shown in yellow. Genes encoding cGAS (dark blue), proteasomal subunits (light blue), CRL5 complex components (dark green) and SPSB3 (light green) are highlighted. **f**, Schematic of CRL5–SPSB3-directed cGAS ubiquitylation. **g**, The relative nuclear cGAS–GFP MFI in post-mitotic HeLa cells treated with siRNA against *SPSB3* (siS*PSB3*; *n* = 18) or *CUL5* (*n* = 14), non-targeting control siRNA (*n* = 16) or epoxomicin (*n* = 14). **h**, QIBC analysis of nuclear cGAS levels in HeLa cells that were treated with siRNA against *SPSB3* or *CUL5* or control siRNA. **i**,**j**, Nuclear (Nuc.) and cytosolic (Cyto.) cGAS–GFP was analysed using immunoblotting in cGAS–GFP-expressing HeLa cells that were treated with siRNA against *SPSB3* or control siRNA (**i**) or MLN4924 (**j**) and incubated with cycloheximide (CHX) for the indicated time courses. H2B and GAPDH were used as the loading controls. For **b**, **d** and **g**, data are mean ± s.d. Statistical analysis was performed using one-way analysis of variance (ANOVA) with Šídák’s multiple-comparison test (**c** and **h**) and two-way ANOVA with Tukey’s multiple-comparison test (**d** and **g**). For **a**, **i** and **j**, one representative experiment of three independent experiments is shown. Source Data

In mitosis, when the nuclear envelope disassembles, cGAS is rapidly recruited onto chromosomes and, through this mechanism, is relocated into the nuclear interior^16,17^ (Fig. 1a and Supplementary Video 1). Recent findings reported that the nuclear pool of cGAS is largely immobile and inactive^9^. This state is achieved through the tight tethering of cGAS to the acidic patch at the nucleosome surface masking essential elements required for DNA binding and enzyme activation^8,10–14^. Suppression of intranuclear cGAS is further aided by the chromatin architectural protein BAF, which shields double-stranded DNA from cGAS binding^15^. These insights elucidated principles of cGAS regulation inside the nucleus. However, how cells coordinate the presence of cGAS on chromatin with general housekeeping genomic processes is unclear.

## Intranuclear degradation of cGAS

**Extended Data Fig. 1:**
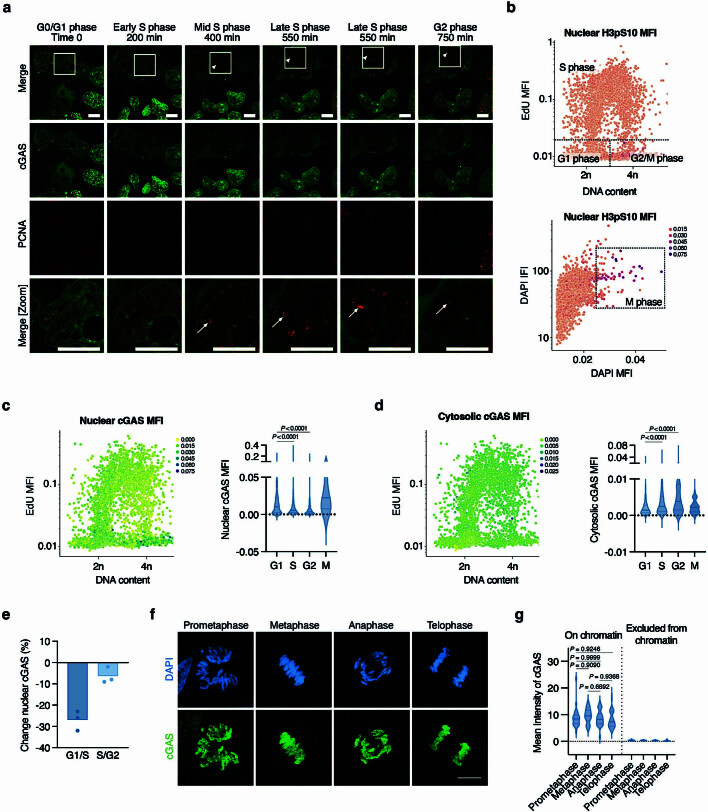
Nuclear cGAS levels decrease in cycling cells. **a**, Representative image sequence demonstrating cGAS intranuclear degradation in HeLa cells expressing cGAS-GFP (green) and PCNA (red) (see Supplementary Video 2). The PCNA distribution pattern is used to determine cell cycle phase with PCNA foci formation (arrows) in mid S phase. Scale bar, 10 μm. **b**, QIBC plots of DNA content versus mean EdU intensity (top) or mean DAPI intensity versus integrated DAPI intensity (bottom), with mean nuclear H3pS10 intensity shown by a colour scale. Dashed lines indicate the labelled cell cycle populations. 2n and 4n refer to the number of sets of chromosomes. **c**, QIBC plot of DNA content versus EdU mean intensity, with mean nuclear cGAS intensity shown by a colour scale (left). QIBC analysis of cGAS-GFP expressing HeLa cells (right). **d**, QIBC plot of DNA content versus EdU mean intensity, with mean cytosolic cGAS intensity shown by a colour scale (left). QIBC analysis of cGAS-GFP expressing HeLa cells (right). **e**, Relative change of nuclear cGAS level in G1 phase versus S phase (G1/S) or S phase versus G2 phase (S/G2) by QIBC analysis of HeLa cells. Dots indicate individual QIBC assays. **f**, **g**, Representative confocal images (**f**) or quantification (**g**) of endogenous cGAS level at different stages of mitosis in HeLa cells. (Prometaphase, *n* = 25; Metaphase, *n* = 34; Anaphase, *n* = 52; Telophase, *n* = 55). Scale bar, 10 μm. Numbers indicate individual cells. Data are mean ± SD (**c**, **d**, **g**). One-way ANOVA with Šídák’s multiple comparison test (**c**, **d**, **g**) was used for statistical analysis. One representative of three independent experiments is shown (**a**). Source Data

To determine the fate of nuclear cGAS, we tracked GFP-tagged cGAS using live-cell imaging analysis of HeLa cells. In agreement with previous observations^16^, we observed that, after mitosis, the abundance of nuclear cGAS progressively decreased over the course of the subsequent cell cycle (Fig. 1a,b, Extended Data Fig. 1a and Supplementary Video 2). Quantitative image-based cytometry (QIBC)^18^ analysis revealed a continuous decrease in nuclear cGAS from G1 to G2 phase of the cell cycle that was most accentuated in G1 phase and paralleled by a complementary increase in cytosolic cGAS levels (Extended Data Fig. 1b–e). During mitosis, when cGAS co-localizes with chromatin, no further change in cGAS levels was recorded (Extended Data Fig. 1f,g). We verified these observations in naive HeLa cells and confirmed that the nuclear pool, but not the cytosolic pool, of endogenous cGAS gradually decreases during interphase (Fig. 1c).

**Extended Data Fig. 2:**
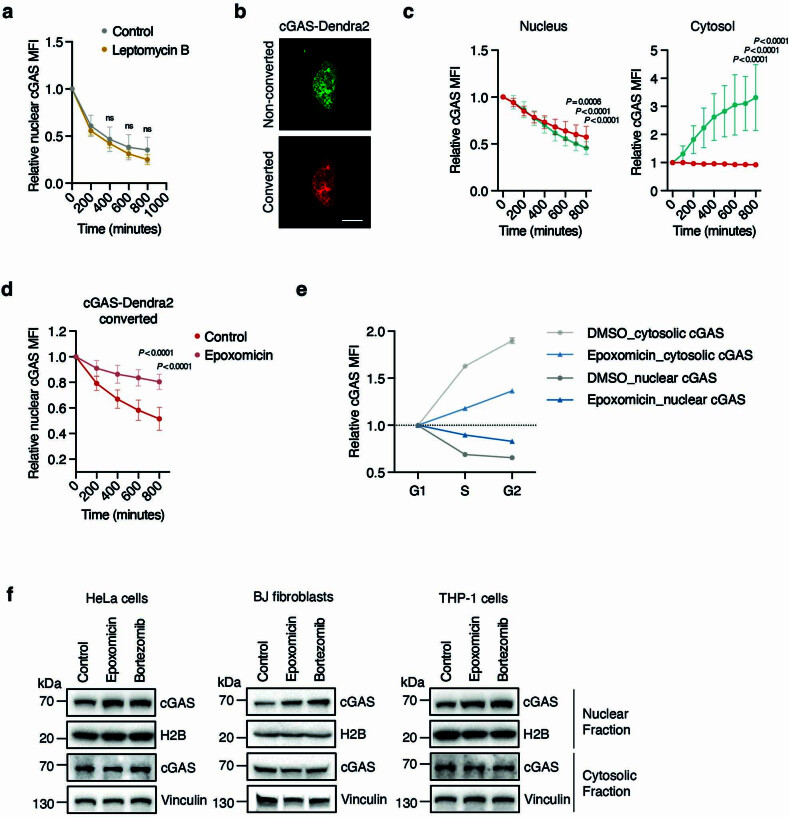
SPSB3 and CUL5 control nuclear cGAS levels. **a**, Relative nuclear cGAS-GFP MFI in post-mitotic HeLa cells treated with leptomycin B (*n* = 20) or DMSO (*n* = 12). **b**, Representative image of a HeLa cell expressing cGAS-Dendra2 before and after photoconversion. Scale bar, 10 μm. **c**, Relative cGAS-Dendra2 MFI in the nucleus (left; *n* = 28 cells per condition) or cytoplasm (right; *n* = 29 cells per condition) in cells that underwent photoconversion (red) or not (green). **d**, Relative nuclear photoconverted cGAS-Dendra2 MFI measurement in post-mitotic HeLa cells treated with epoxomicin (*n* = 27) or DMSO (*n* = 25). **e**, QIBC analysis of endogenous cGAS levels in HeLa cells treated with epoxomicin or DMSO. **f**, Cytosolic and nuclear fractions were extracted from indicated cells treated with DMSO, epoxomicin or bortezomib for 16 h and analysed by immunoblot. Vinculin and H2B were used as loading control for cytosolic and nuclear fractions, respectively. Data are mean ± SD (**a**, **c**, **d**) or mean ± SEM (**e**). Numbers indicate individual cells (**a**, **c**, **d**). *P* values were obtained by two-way ANOVA with Šídák’s multiple comparison test (**a**, **c**, **d**). One representative of three (**f**) independent experiments is shown. Source Data

Nuclear export of cGAS might account for the observed changes in subcellular cGAS distribution^19^. However, chemical inhibition of the major nuclear export machinery by leptomycin B did not affect the decline in nuclear cGAS (Extended Data Fig. 2a). Moreover, selective tracking of nuclear cGAS using a photoconvertable fusion construct (cGAS–Dendra2) did not reveal cytosolic accumulation of photoconverted cGAS, indicating that cGAS is not transported from the nucleus to the cytosol (Extended Data Fig. 2b,c). To explain the nuclear loss of cGAS, we considered protein degradation through the UPS^20^. Live-cell imaging showed that blocking the proteasome with epoxomicin or bortezomib stabilized intranuclear cGAS (Fig. 1d, Extended Data Fig. 2d and Supplementary Videos 3 and 4). The stabilizing effect of proteasome inhibitors on endogenous nuclear cGAS was verified by QIBC and observed in several human cell lines (Extended Data Fig. 2e,f). These results establish proteolysis through the UPS as an essential principle in the regulation of nuclear cGAS.

## Identification of CRL5–SPSB3 in cGAS control

**Extended Data Fig. 3:**
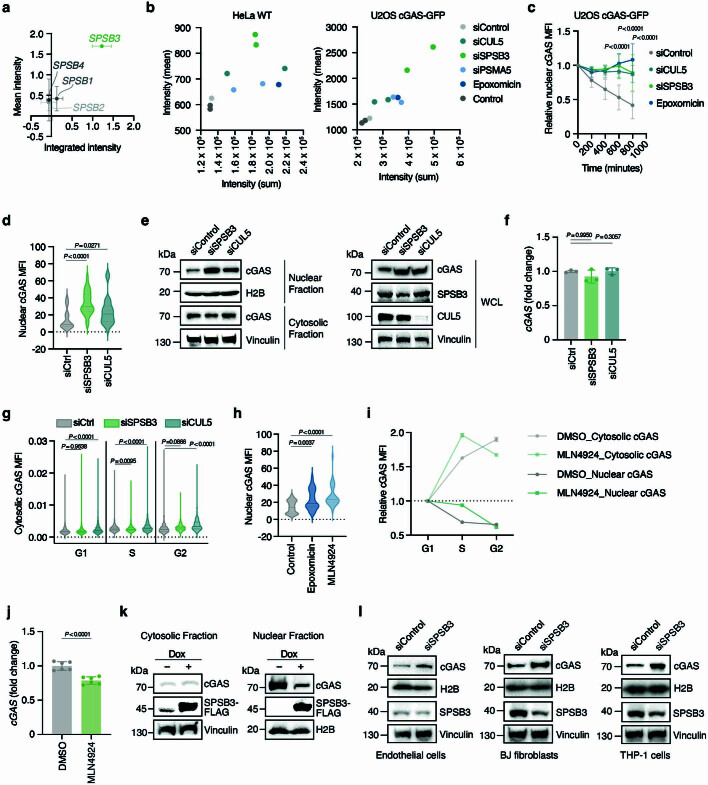
SPSB3 targets nuclear cGAS. **a**, Results from the siRNA screen highlighting cGAS-GFP nuclear abundance by mean fluorescence intensity and integrated fluorescence intensity in cells treated with siRNAs against SPSB family members (*n* = 3). **b**, Nuclear cGAS measurements by confocal microscopy in HeLa cells (endogenous cGAS) or U2OS cells (cGAS-GFP expression) transfected with siRNAs against *CUL5*, *SPSB3* and *PSMA5* or control siRNA or treated with epoxomicin or DMSO (*n* = 2). **c**, Relative nuclear cGAS-GFP MFI measurement in post-mitotic U2OS cells treated with siRNA against *SPSB3* (*n* = 15) or *CUL5* (*n* = 15), control siRNA (*n* = 14) or epoxomicin (*n* = 15). **d**, Nuclear cGAS-GFP measurements by confocal microscopy in HeLa cells treated with siRNA against *SPSB3* (*n* = 31) or *CUL5* (*n* = 33) or control siRNA (*n* = 22). **e**, Whole cell lysates, cytosolic and nuclear fractions were extracted from HeLa cells transfected with non-targeting control siRNA of siRNAs targeting *SPSB3* or *CUL5* and analysed by immunoblot. Vinculin and H2B were used as loading control of whole cell lysates, cytosolic and nuclear fractions, respectively. **f**, mRNA levels of cGAS were measured by RT-qPCR in HeLa cells treated with non-targeting control siRNA or siRNAs against *SPSB3* or *CUL5* for 5 days. Ratios of relative cGAS mRNA levels normalized to the control are shown (*n* = 3). **g**, QIBC analysis of endogenous cytosolic cGAS level in HeLa cells treated with siRNA against *SPSB3*, *CUL5* or control siRNA. **h**, Nuclear cGAS-GFP measurements by confocal microscopy in HeLa cells treated with epoxomicin (*n* = 32), MLN4924 (*n* = 29), or DMSO (*n* = 32). **i**, QIBC analysis of endogenous cGAS levels in HeLa cells treated with MLN4924 or DMSO. **j**, mRNA levels of cGAS were measured by RT-qPCR in HeLa cells treated with MLN4924 or DMSO. Ratios of relative cGAS mRNA levels normalized to the control are shown (*n* = 6). **k**, Cytosolic and nuclear fractions were extracted from HeLa cells expressing doxycycline (Dox)-inducible *SPSB3* that were treated or not with Dox for 4 days). Immunoblots probing cGAS and SPSB3 (FLAG) are shown. Vinculin and H2B were used as loading control for cytosolic and nuclear fractions, respectively. **l**, Whole cell lysates and nuclear fractions collected from primary endothelial cells, BJ-5ta fibroblasts or differentiated THP-1 cells treated with non-targeting control siRNA or siRNA against *SPSB3* for 5 days were analysed by immunoblot. Vinculin and H2B were used as loading control for whole cell lysates and nuclear fractions, respectively. Numbers indicate individual cells (**c**, **d**, **h**) or technical replicates(**a**, **f**, **j**). Data are mean ± SD (**a**, **c**, **d**, **f**, **g**, **h**, **j**) or mean ± SEM (**i**). *P* values were obtained by two-way ANOVA with Tukey’s multiple comparison test (**c**), one-way ANOVA with Dunnett’s multiple comparison test (**d**, **h**), one-way ANOVA (**f**), one-way ANOVA with Šídák’s multiple comparison test (**g**) or two-tailed Student’s *t*-test (**j**). One representative of three (**e**, **f**, **k**-**l**) independent experiments is shown. Source Data

To identify factors involved in nuclear cGAS degradation, we conducted an RNA interference (RNAi)-based genetic screen targeting 972 genes associated with the UPS and monitored nuclear cGAS abundance using confocal microscopy (Supplementary Table 1). Validating the experimental approach, siRNA-mediated knockdown of distinct subunits of the proteasome abrogated nuclear cGAS degradation (Fig. 1e and Supplementary Table 1). Notably, among the top scoring hits, we identified all components of the core multisubunit CRL5 scaffolding complex (*CUL5* (encoding cullin 5), *RBX2* (encoding RING-box protein 2), *NEDD8*, *TCEB1* and *TCEB2*), the CUL5-specific NEDD8 E2 ligase UBE2F and a poorly characterized substrate receptor SPSB3^21–24^ (Fig. 1e,f and Supplementary Table 1). SPRY-domain- and SOCS-box-containing (SPSB) proteins bind—through their SOCS box—to the elongin B–elongin C (ELOBC) heterodimeric adaptor connecting to the CUL5–RBX2 complex and recruit substrates through the SPRY domain specifying protein ubiquitylation^22,25^. In contrast to SPSB3, none of the other SPSB family members scored as hits in the screen, implying a specific role for SPSB3 in controlling the stability of nuclear cGAS protein (Extended Data Fig. 3a and Supplementary Table 1). Using live-cell imaging, confocal microscopy and western blot analysis, we independently confirmed that a knockdown of *SPSB3* and *CUL5* attenuated the degradation of nuclear, but not cytosolic, cGAS, while leaving *CGAS* transcript levels unaffected (Fig. 1g and Extended Data Fig. 3b–f). *SPSB3* and *CUL5* depletion rescued nuclear cGAS degradation during the entire interphase (Fig. 1h and Extended Data Fig. 3g). Likewise, broad pharmacological targeting of cullin–RING E3 ligases by the neddylation inhibitor MLN4924 blocked nuclear cGAS degradation (Extended Data Fig. 3h–j). Reciprocally, overexpression of *SPSB3* reduced nuclear cGAS levels (Extended Data Fig. 3k). Consistent with a function in selectively controlling nuclear cGAS turnover, a knockdown of *SPSB3* or treatment with MLN4924 increased the half-life of nuclear cGAS in cycloheximide chase experiments (Fig. 1i,j). Regulation of endogenous nuclear cGAS protein levels by SPSB3 was validated in various human cell lines and primary human endothelial cells (Extended Data Fig. 3l). Together, these data identify a role for the CUL5–RBX2–ELOBC E3 ligase assembly and its substrate receptor SPSB3, a complex hereafter referred to as CLR5–SPSB3, in the control of nuclear cGAS stability.

## CLR5^SPSB3^ ubiquitylates cGAS

**Fig. 2:**
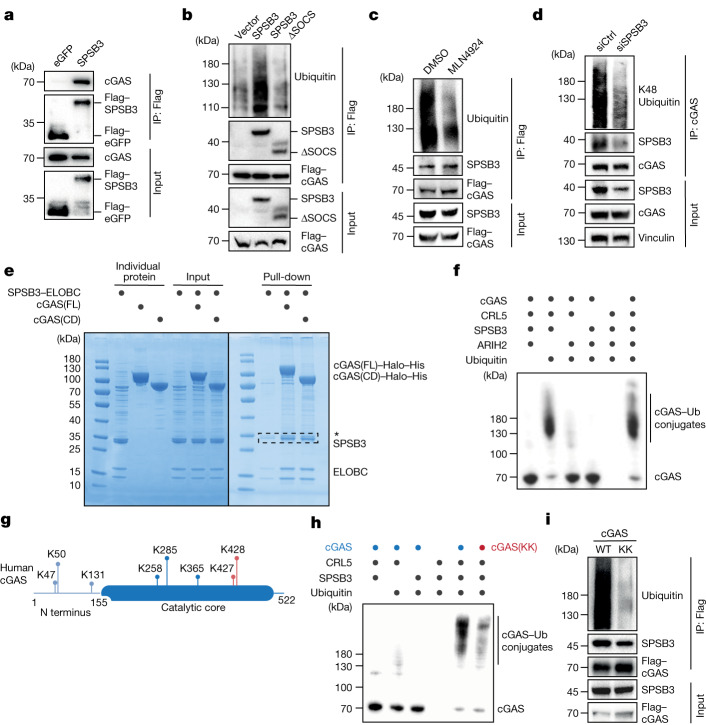
CRL5–SPSB3 ubiquitylates cGAS. **a**, Immunoprecipitation (IP) of Flag-tagged SPSB3 or Flag-tagged eGFP from HEK293T cells transfected with cGAS and SPSB3-Flag or eGFP-Flag followed by immunoblot analysis. **b**, IP of Flag-tagged cGAS from HEK293T cells transfected with constructs for Flag-tagged cGAS, HA-tagged ubiquitin, empty vector, SPSB3 or SPSB3(ΔSOCS) followed by immunoblot analysis. **c**, IP of Flag-tagged cGAS from HEK293T cells transfected with cGAS-Flag, HA-tagged ubiquitin and SPSB3 followed by treatment with DMSO or MLN4924. The samples were analysed by immunoblotting. **d**, HeLa cells were treated with control siRNA or siRNA targeting *SPSB3* for 3 days and analysed using immunoprecipitation with anti-cGAS antibodies. The samples were analysed by immunoblotting. **e**, In vitro Ni-NTA pull-down assaying cGAS complex formation with the SPSB3–ELOBC heterotrimer. His–Halo-tagged cGAS full-length (FL) or catalytic domain (CD) cGAS was used. **f**, Immunoblot analysis showing in vitro ubiquitylation reactions of cGAS by CRL5–SPSB3 in the presence or absence of the RBR E3 ligase ARIH2. **g**, Ubiquitylation sites of human cGAS identified by MS. Lysine residues present in the conserved catalytic core were further validated. **h**, Immunoblot analysis showing in vitro ubiquitylation reactions of WT cGAS and cGAS(K427R/K428R) mutant (cGAS(KK)) by CRL5–SPSB3. **i**, IP of Flag-tagged cGAS from HEK293T cells transfected with Flag-tagged WT-cGAS or cGAS(KK), HA-tagged ubiquitin and SPSB3. The samples were analysed by immunoblotting. Vinculin was used as the loading control. One representative experiment of two (**a** and **i**) or three (**b**–**f** and **h**) independent experiments is shown.

**Extended Data Fig. 4:**
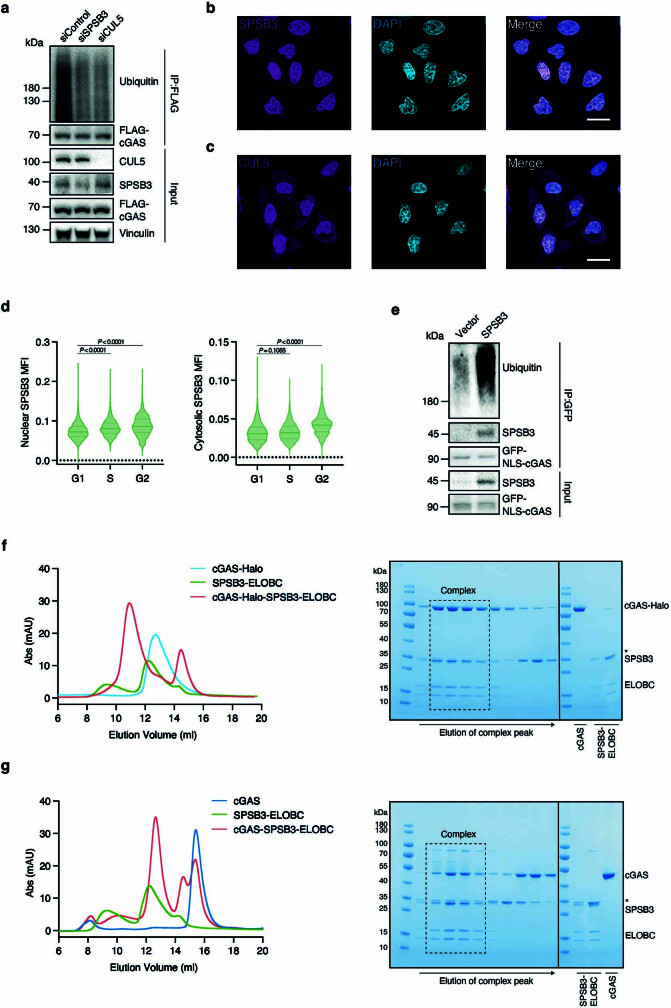
CRL5^SPSB3^ targets nuclear cGAS. **a**, IP of Flag-tagged cGAS from HEK293T cells transfected with cGAS-Flag and HA-ubiquitin and treated with indicated siRNAs. Samples were analysed by immunoblot. Vinculin was used as a loading control. **b**, **c**, Representative confocal microscopy images of HeLa cells stained for SPSB3 (**b**) or CUL5 (**c**) and DAPI (blue). Scale bars, 20 μm. **d**, QIBC analysis of endogenous nuclear (left) or cytosolic (right) SPSB3 levels in HeLa cells. **e**, IP of GFP-tagged NLS-cGAS from HEK293T cells transfected with constructs for GFP-tagged NLS-cGAS, HA-tagged ubiquitin with or without SPSB3. Samples were analysed by immunoblot. **f**, Size-exclusion chromatography (SEC) and SDS–PAGE of an assembled Halo-cGAS-SPSB3-ELOBC complex. **g**, SEC and SDS–PAGE of an assembled cGAS-SPSB3-ELOBC complex. Data are mean ± SD (**d**). *P* values were obtained by one-way ANOVA with Šídák’s multiple comparison test (**d**). One representative of three (**a**-**d**) or two (**e**-**g**) independent experiments is shown. Source Data

We next performed immunoprecipitation experiments to assess cGAS binding and ubiquitylation by CLR5^SPSB3^. SPSB3 efficiently immunoprecipitated cGAS after ectopic expression in HEK293T cells (Fig. 2a). Furthermore, expression of SPSB3, but not a SPSB3 mutant with a defective SOCS box, promoted cGAS ubiquitylation and this effect was blunted by treatment with MLN4924 (Fig. 2b,c). Conversely, depletion of *SPSB3* or *CUL5* reduced the ubiquitylation of ectopically expressed cGAS (Extended Data Fig. 4a). We targeted *SPSB3* in naive HeLa cells by siRNA and observed reduced Lys48-linked ubiquitylation of endogenous cGAS, consistent with a role of CRL5–SPSB3 in promoting proteasomal degradation of the protein (Fig. 2d). Both CUL5 and SPSB3 localize inside the nucleus and nuclear SPSB3 levels moderately increase during interphase (Extended Data Fig. 4b–d). SPSB3 efficiently modifies cGAS fused to a nuclear-localization signal (NLS), verifying that SPSB3 directs CLR5 ubiquitin ligase activity against the nuclear cGAS pool (Extended Data Fig. 4e).

**Extended Data Fig. 5:**
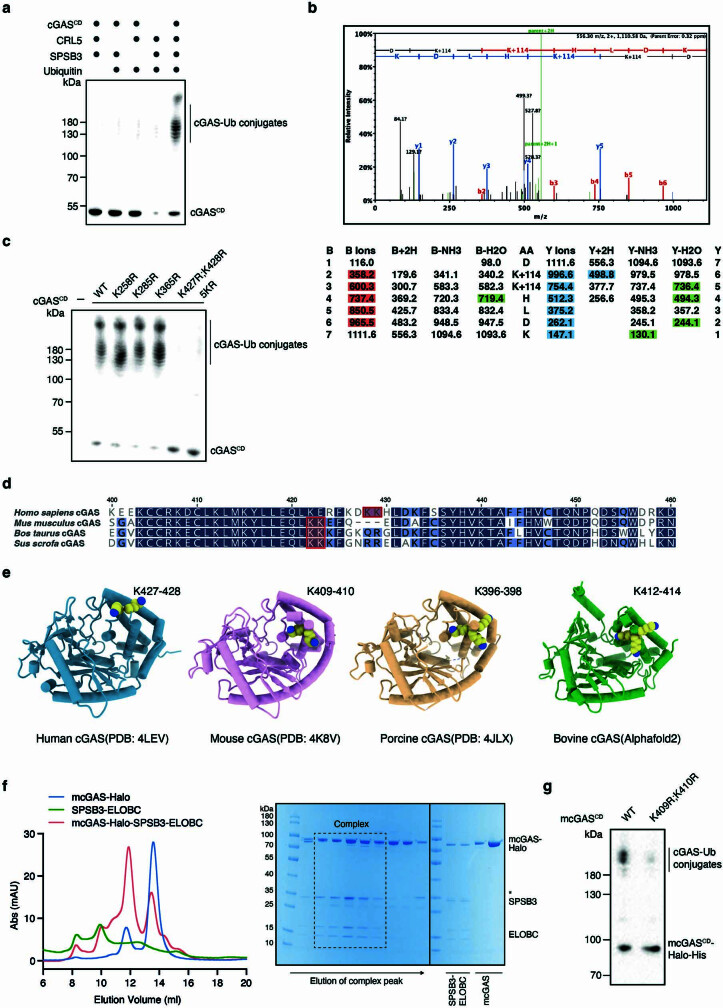
Conserved mechanisms of cGAS ubiquitylation by CRL5^SPSB3^. **a**, Immunoblot showing in vitro ubiquitylation reactions of human cGAS catalytic domain (CD; aa: 155–522 aa) by CRL5^SPSB3^. **b**, Representative spectrum of a peptide comprising ubiquitylated K427 and K428 lysine residues identified by mass spectrometry. The sequence of the peptide, DKKHLDK, is shown at top of the spectrum and ubiquitylation at K427 and K428 is indicated by the 114 increases of mass, as shown in the fragmentation table below. **c**, Immunoblot showing in vitro ubiquitylation reactions of wild-type cGAS CD and distinct lysine residue substitutions of cGAS CD by CRL5^SPSB3^. **d**, Sequence alignment of amino acids surrounding K427 and K428 in human cGAS, murine cGAS, bovine cGAS or porcine cGAS. The K427/K428 lysine pair in human cGAS subject to ubiquitylation by CRL5^SPSB3^ is highlighted in dark red and the structurally conserved lysine pair of the other cGAS homologues as shown in (**e**) are highlighted in red. **e**, Comparison of human cGAS (PDB: 4LEV)^42^, mouse cGAS (PDB: 4K8V)^5^, porcine cGAS (PDB: 4JLX)^36^ and bovine cGAS (Alphafold2 prediction). The lysine pair on cGAS is rendered by sphere. **f**, Size-exclusion chromatography and SDS–PAGE of an assembled mouse cGAS-Halo-SPSB3-ELOBC complex. **g**, Immunoblot showing in vitro ubiquitylation reactions of wild-type mouse cGAS CD (aa: 147–507) and the corresponding K409R/K410R mutant by CRL5^SPSB3^. Representatives of at least two independent experiments (**a**, **c**, **f**, **g**) are shown. Source Data

To directly examine cGAS recognition by SPSB3, we reconstituted SPSB3-target binding in vitro using a truncated version of SPSB3 comprising the core structured part (amino acids 83–326) stabilized by the ELOBC heterodimer (Supplementary Note 1). Recombinant cGAS robustly interacted with the SPSB3–ELOBC heterotrimeric complex (Fig. 2e and Extended Data Fig. 4f,g). Deletion of the unstructured N terminus of cGAS (cGAS catalytic domain; amino acids 155–522) did not affect its interaction with the substrate receptor complex, suggesting that SPSB3 recognizes an element within the cGAS catalytic core (Fig. 2e). In vitro ubiquitylation assays demonstrated that the minimal functional CRL5–SPSB3 ligase complex promotes ubiquitylation of full-length cGAS and its catalytic domain (Fig. 2f and Extended Data Fig. 5a). Consistent with accelerating CLR5-dependent ubiquitylation through E3–E3 super-assembly formation, addition of the RING-between-RING (RBR) E3 ligase ARIH2 enhanced cGAS ubiquitylation in vitro^26,27^ (Fig. 2f). To determine which lysine residues are targeted by CRL5–SPSB3, we analysed ubiquitylated cGAS using mass spectrometry (MS), which revealed five sites modified by CRL5–SPSB3 that are situated in the catalytic core region (Fig. 2g and Extended Data Fig. 5b). Mutagenesis identified the neighbouring lysine pair Lys427-Lys428 as the major residues ubiquitylated by CRL5–SPSB3 with minor contributions emerging from N-terminal lysine residues (Fig. 2h and Extended Data Fig. 5c). Substitution of Lys427-Lys428 largely abolished cGAS ubiquitylation and nuclear degradation in cells, corroborating the critical role of these residues in dictating cGAS protein stability (Fig. 2l). Notably, despite the lack of sequence identity for the Lys427-Lys428 lysine pair at the amino acid level for cGAS orthologues, cGAS from mouse and other non-primate mammals encode a conserved, surface-exposed lysine pair (for example, *Mus musculus* cGAS Lys409-Lys410) in a region upstream within the same C terminal α-helix (Extended Data Fig. 5d,e). We verified robust ubiquitylation of the mouse cGAS catalytic domain by CLR5–SPSB3 in vitro and confirmed that the mouse cGAS Lys409-Lys410 lysine pair is the major target site of ubiquitin modification (Extended Data Fig. 5f,g). Together, these results demonstrate cGAS ubiquitylation by CLR5–SPSB3 and establish conserved targeting of a specific lysine pair within the catalytic core as the underlying mechanism of cGAS degradation.

## Structure of the cGAS–SPSB3 complex

**Extended Data Fig. 6:**
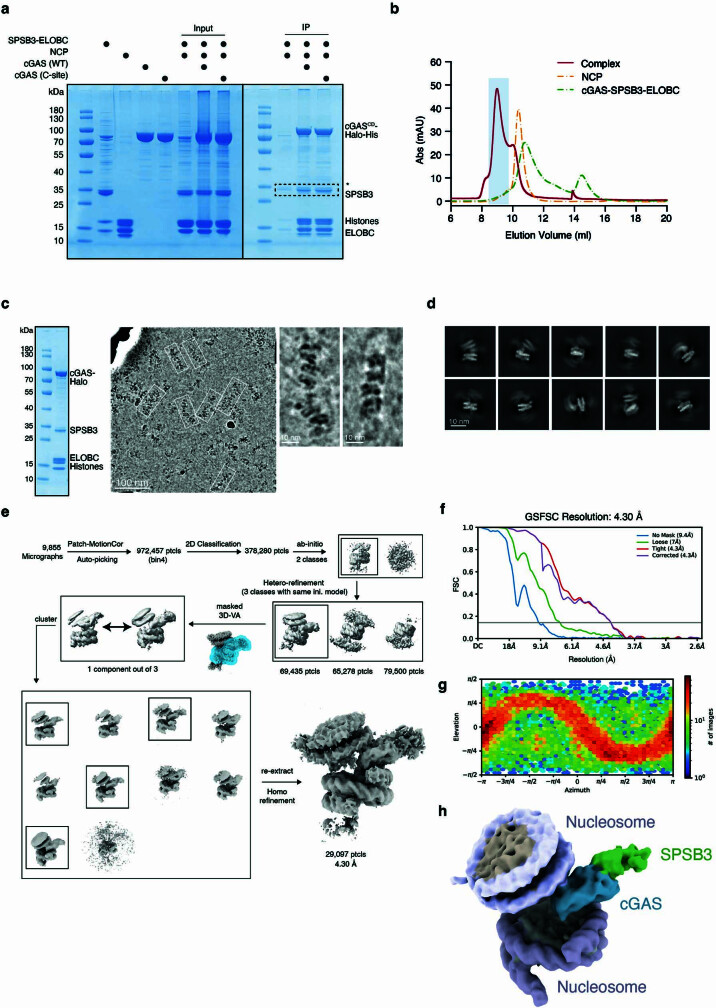
Purification and cryo-EM data processing of nucleosome-bound cGAS in complex with SPSB3-ELOBC. **a**, In vitro Ni-NTA pull-down assaying nucleosome-bound His-Halo-tagged wild-type (WT) cGAS or site C mutant cGAS (K285A/R300A/K427A) complex formation with the SPSB3-ELOBC heterotrimer. **b**, Size-exclusion chromatography of nucleosome-bound cGAS in complex with SPSB3-ELOBC. **c**, Left: Coomassie blue staining SDS-PAGE and cryo-EM analysis of the complex peak (red) shown in (**b**). Middle: Representative micrograph of the nucleosome-cGAS-SPSB3-ELOBC complex in vitrified ice from 9,855 raw images. Scale bar, 100 nm. Right: Zoomed-in cutaways highlighting the complex fibre. Scale bar, 10 nm. **d**, Selected 2D class averages of nucleosome-cGAS-SPSB3-ELOBC complex particles. Scale bar, 10 nm. **e**, Cryo-EM data processing flow chart. **f**, Corrected gold-standard Fourier shell correlation (FSC) curves of the nucleosome-cGAS-SPSB3-ELOBC complex for the 3D EM reconstruction maps. **g**, Angular distribution of nucleosome-cGAS-SPSB3-ELOBC complex particles included in the final reconstruction. **h**, Final 3D reconstruction of the nucleosome-cGAS-SPSB3-ELOBC complex; cGAS in blue, SPSB3 in green, nucleosomal DNA in lilac, histones in grey. One representative of at least three independent experiments is shown (**a**, **b**, **c**). Source Data

**Extended Data Fig. 7:**
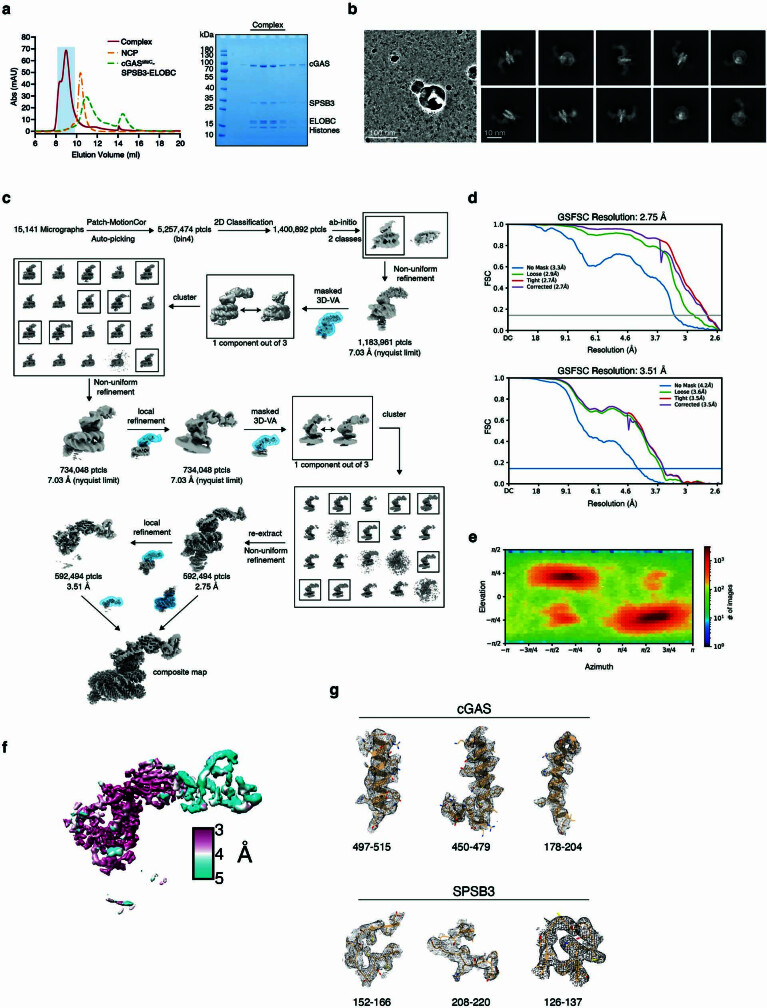
Purification and cryo-EM data processing of nucleosome-bound cGAS C site mutant in complex with SPSB3-ELOBC. **a**, Left: Size-exclusion chromatography of nucleosome-bound cGAS C site mutant (K285A/R300A/K427A) in complex with SPSB3-ELOBC. Right: Coomassie blue staining SDS-PAGE and cryo-EM analysis of the complex peak (red). **b**, Representative micrograph and selected 2D class averages of nucleosome-cGAS^siteC^-SPSB3-ELOBC complex particles in vitrified ice from 15,141 raw images. Scale bar left, 100 nm; scale bar right, 10 nm. **c**, Cryo-EM data processing flow chart. **d**, Corrected gold-standard Fourier shell correlation (FSC) curves of the consensus 3D reconstruction of the nucleosome-cGAS^siteC^-SPSB3-ELOBC complex (top) and the focused 3D reconstruction with a mask on cGAS^siteC^-SPSB3-ELOBC (bottom). **e**, Angular distribution of the nucleosome-cGAS^siteC^-SPSB3-ELOBC complex particles included in the final reconstruction. **f**, Final 3D reconstruction of the cGAS^siteC^-SPSB3-ELOBC complex coloured according to the local resolution. **g**, Cryo-EM density maps (grey mesh) of cGAS^siteC^ and SPSB3 contoured at 5σ. The protein structures fitted into the density map are shown by stick models. One representative of at least three independent experiments is shown (**a**, **b**). Source Data

Inside the nucleus, cGAS is attached to chromatin by strong interactions with the histone H2A–H2B-formed ‘acidic patch’ on the surface of the nucleosome^9–14^. Considering a role in nuclear cGAS regulation, we therefore hypothesized that SPSB3 might recognize nucleosome-bound cGAS. Consistent with this idea, immunoprecipitation assays and size-exclusion chromatography (SEC) indicated that SPSB3–ELOBC forms a complex with nucleosome-bound cGAS (Extended Data Fig. 6a,b). Human cGAS, but not mouse cGAS, is capable of cross-bridging individual nucleosomes in vitro based on contacts between basic surface patches of the human-specific third DNA-binding site, the C site, and nucleosomal DNA^10,13,28^. Mutational analysis showed that C site residues are not involved in cGAS binding to SPSB3, an observation that is consistent with a shared mechanism of nuclear cGAS detection by the SPSB3 substrate receptor (Extended Data Figs. 6a and 7a).

**Fig. 3:**
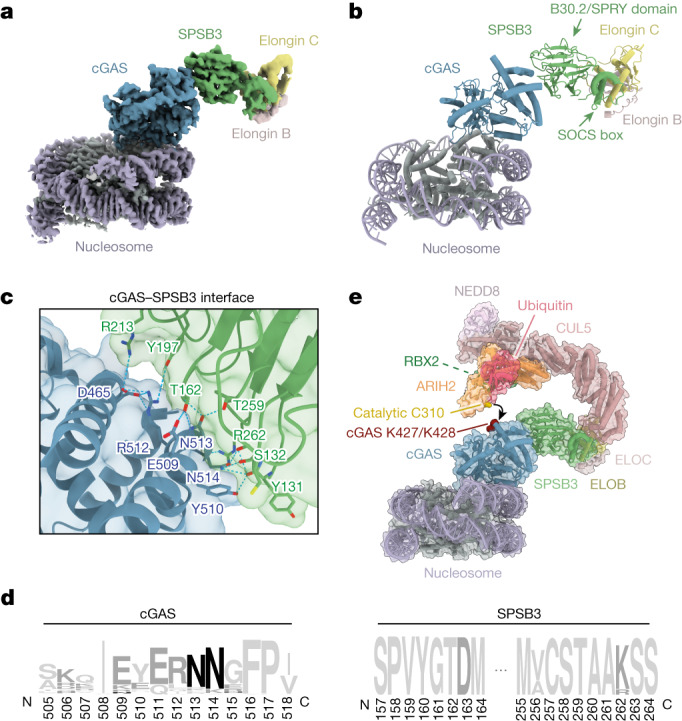
The structural basis of cGAS targeting by SPSB3. **a**, A composite cryo-EM density map of the nucleosome–cGAS–SPSB3–ELOBC complex, assembled from two focused-refinement maps (nucleosome–cGAS and cGAS–SPSB3–ELOBC). Different contour levels were used for optimal visualization using UCSF ChimeraX^37^. cGAS(site C), mutated in the DNA-binding C site, was used to obtain this dataset. **b**, Ribbon representation of the nucleosome–cGAS–SPSB3–ELOBC complex structure. The arrows indicate the B30.2/SPRY and SOCS box domains of SPSB3. **c**, Detailed view of the binding interface between cGAS and SPSB3. **d**, Sequence logo analysis of the cGAS and SPSB3 interface derived from 150 vertebrate species. **e**, Model of nucleosome-bound cGAS targeted by an activated (neddylated) CRL5–SPSB3 complex with RBR E3 ligase ARIH2 priming polyubiquitylation by transferring the first ubiquitin onto cGAS. Lysine residues that have been identified as cGAS ubiquitylation sites by MS and are essential for SPSB3-mediated degradation of cGAS are coloured red; the catalytic Cys310 of ARIH2 is coloured gold. The model was built by docking the nucleosome–cGAS–SPSB3–ELOBC model into a model comprising the ELOBC–CUL5 (Protein Data Bank (PDB): 4JGH)^33^, CUL5–NEDD8–RBX2–ARIH2 (PDB: 7ONI)^31^ and ARIH1–UB (PDB: 7B5M)^32^ complexes.

**Extended Data Fig. 8:**
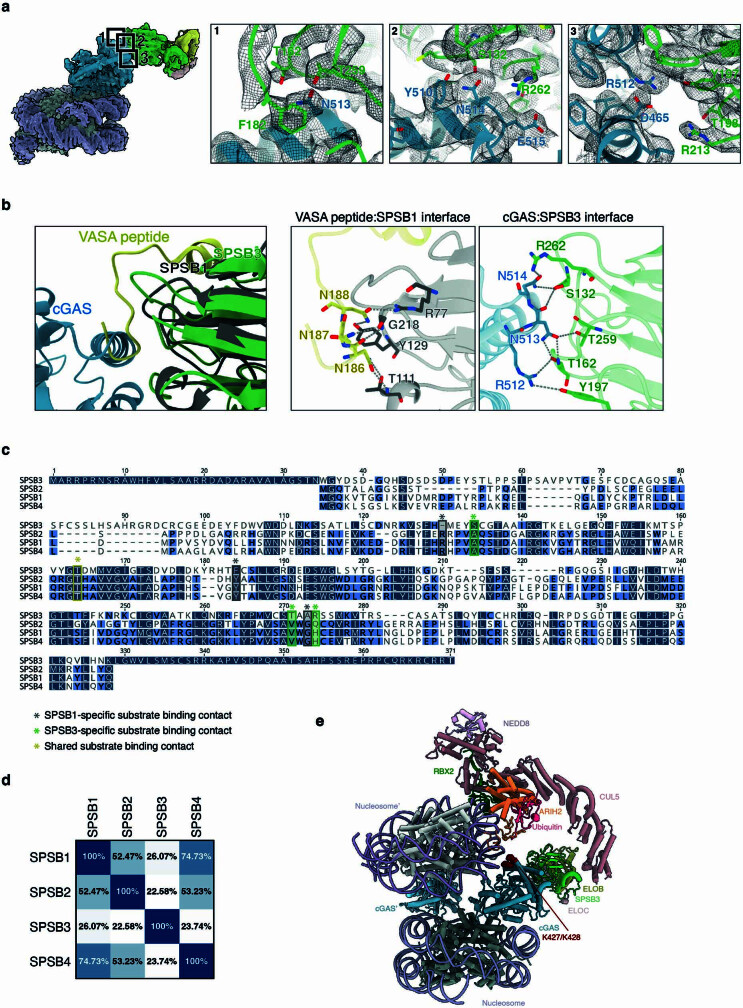
A unique interface on SPSB3 for binding cGAS. **a**, Right: Ribbon diagram and a 3D reconstruction of the nucleosome-cGAS^siteC^-SPSB3-ELOBC complex. cGAS in blue, SPSB3 in green, nucleosomal DNA in lilac, histones in grey, ELOB in yellow, ELOC in antique white. Left: EM densities (shown as mesh at 5σ) for cGAS residues interacting with the SPSB3. Shown are cGAS N513, N514 and R512/D465 interactions with SPSB3 respectively. **b**, The cGASSPSB3 model is aligned with the VASA-SPSB1 model (PDB: 3F2O)^29^, individual interacting residues are shown by sticks. **c**, Sequence alignment of SPSB1/2/3/4. Residues used for substrate recognition are highlighted. **d**, Percent identity matrix of human SPSB1/2/3/4. **e**, Model of the nucleosome/cGAS 2:2 sandwich targeted by an activated (neddylated) CRL5-SPSB3 complex with RBR E3 ligase ARIH2 priming polyubiquitylation by transferring the first ubiquitin onto cGAS. cGAS K427/K428 is competitively targeted by either nucleosomal DNA or the E3 ligase. The model is built by docking the nucleosome-cGAS-SPSB3-ELOBC model into a model composed of the nucleosome/cGAS 2:2 complex (PDB: 6Y5D)^10^, ELOBC-CUL5 (PDB: 4JGH)^33^, CUL5-NEDD8-RBX2-ARIH2 (PDB: 7ONI)^31^, and ARIH1-UB (PDB: 7B5M)^32^ complexes.

To define the molecular basis of cGAS recognition by SPSB3, we determined cryo-electron microscopy (cryo-EM) structures of wild-type (WT) and C site-mutated cGAS (cGAS(K285A/R300A/K427A), hereafter cGAS(site C)) catalytic core domains in a complex with a nucleosome core particle and the SPSB3–ELOBC heterotrimer (Fig. 3a,b, Extended Data Figs. 6c–g and 7b–g and Extended Data Table 1). The overall topology of the cGAS–SPSB3 subcomplex within the two distinct maps is identical, but the complex incorporating WT cGAS is not as well resolved, probably owing to the heterogeneity of the sample with formation of varying higher-order cGAS-bridged nucleosome fibres (Extended Data Fig. 6c). We therefore focused on the structural analysis of the SPSB3–cGAS(site C) complex. A 3.5 Å final reconstruction of this complex reveals an intermolecular interface contributed by the C-terminal helix on cGAS (amino acids 497–515) and five variable loops extending from the bent β-sandwich core of SPSB3 opposite face of the SOCS box domain (Fig. 3a–c). The cGAS residues Asn513 and Asn514 form hydrogen bonds to SPSB3 loop residues Ser132, Thr162, Thr259 and Arg262 (Fig. 3c and Extended Data Fig. 8a). Moreover, a set of acidic residues on cGAS, including Asp465, Glu509, Tyr510, Arg512 and Glu515, bridges to residues Thr162, Tyr197 and Arg213 on SPSB3 through electrostatic interactions reinforcing complex formation (Fig. 3c and Extended Data Fig. 8a). Although the overall composition of the substrate-recognition module—comprising the connecting loops extruding from the central SPRY domain^29^—is identical across SPSB paralogues, the residues involved in side-chain contacts to the substrate are unique to SPSB3, reflecting its specific role in cGAS binding (Extended Data Fig. 8b–d). Accordingly, the cGAS interface gives rise to a distinctive NN minimal sequence-recognition motif differing from the previously described SPSB-recognized consensus motif^29,30^. Notably, contacts comprising these major interactions are shared across all vertebrate DNA-sensing cGAS enzymes and SPSB3 receptors, respectively, defining a conserved mechanism of cGAS protein targeting by E3 ligases (Fig. 3d).

To model ubiquitylation of cGAS by CRL5–SPSB3, we docked an activated (neddylated) ELOBC–CRL5–ARIH2 E3–E3 complex^31–33^ to the SPSB3–cGAS–nucleosome assembly^10^ (Fig. 3e). In this model, cGAS and ubiquitin-bound ARIH2 are situated at opposite ends of the bent and elongated CRL5–SPSB3 core, facilitating ARIH2 to reach across the SPSB3-bound cGAS substrate for ubiquitin ligation. Notably, the ARIH2 active-site cysteine is juxtaposed with Lys427 and Lys428 of cGAS, rationalizing preferential ligation of this particular substrate site. Human cGAS, but not mouse cGAS, can bridge individual nucleosomes through C-site contacts that engage a second nucleosome in vitro^10,13^. Modelling the 2:2 cGAS–nucleosome complex onto the cGAS–SPSB3–ELOBC–CLR5 complex results in steric clashes between the second nucleosome and catalytic subunits of CRL5 (Extended Data Fig. 8e). Thus, the mononucleosome–cGAS complex is the substrate for CRL5–SPSB3 ubiquitylation.

**Fig. 4:**
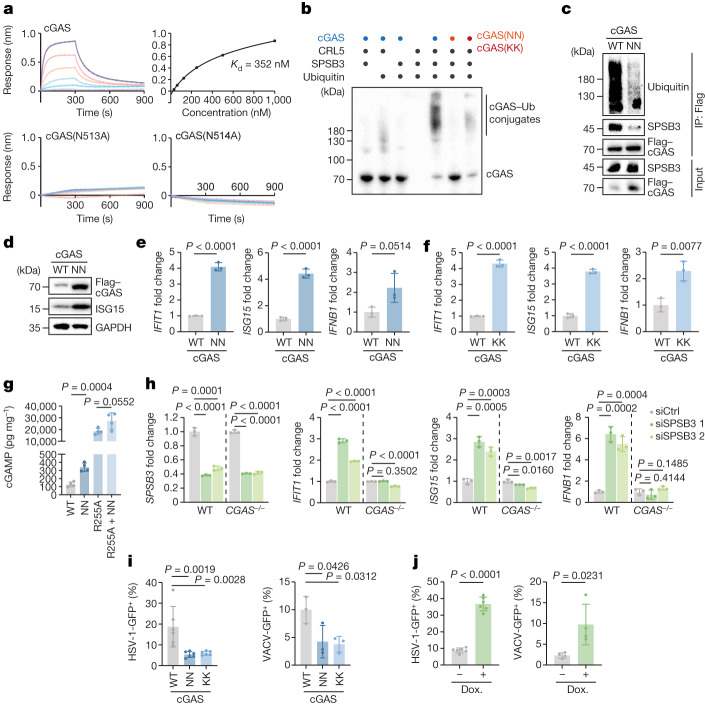
Defective nuclear cGAS ubiquitylation elevates the cellular type I IFN tone. **a**, BLI binding assays of His–Halo-tagged cGAS, cGAS(N513A) or cGAS(N514A) with SPSB3–ELOBC. The binding affinity of WT cGAS with SPSB3–ELOBC is shown at the top right. **b**, Immunoblot showing in vitro ubiquitylation reactions of cGAS, cGAS(NN) or cGAS(KK) by CRL5–SPSB3. **c**, IP of Flag-tagged cGAS from HEK293T cells transfected with Flag-tagged WT cGAS or cGAS (NN), HA-ubiquitin and SPSB3 followed by immunoblot analysis. **d**, Immunoblot analysis of the levels of cGAS or cGAS(NN) induced in HeLa *CGAS*-KO cells by doxycycline. GAPDH was used as the loading control. **e**,**f**, The expression of *IFIT1*, *ISG15* and *IFNB1* was assessed using quantitative PCR with reverse transcription (RT–qPCR) after induction of cGAS, cGAS(NN) (**e**) or cGAS(KK) (**f**) using doxycycline. The relative mRNA levels normalized to the control are shown. *n* = 3. **g**, cGAMP production in HeLa *CGAS*-KO cells reconstituted with doxycycline-inducible cGAS constructs. *n* = 4. **h**, The mRNA levels of *SPSB3*, *IFIT1*, *ISG15* and *IFNB1* were measured using RT–qPCR in HeLa and HeLa *CGAS*-KO cells treated with non-targeting control siRNA or *SPSB3* siRNAs for 5 days. The relative mRNA levels normalized to the control are shown. *n* = 3. **i**, *CGAS*-KO HeLa cells expressing doxycycline-inducible cGAS, cGAS(NN) or cGAS(KK) were infected with HSV-1-GFP (left; *n* = 6) or VACV-GFP (right; *n* = 3) and analysed using flow cytometry for GFP expression. **j**, HeLa cells expressing doxycycline-inducible SPSB3 or not were infected with HSV-1-GFP (left; *n* = 6) or VACV-GFP (right; *n* = 4) and analysed using flow cytometry. For **e**–**j**, Data are mean ± s.d. The numbers indicate technical replicates (**e**–**f** and **h**) or independent biological experiments (**g**, **i** and **j**). *P* values were calculated using one-way ANOVA (**g**–**i**) or two-tailed Student’s *t*-tests (**e**, **f** and **j**). One representative experiment of two (**a**–**c**) or three (**d**–**f** and **h**) independent experiments is shown. Source Data

**Extended Data Fig. 9:**
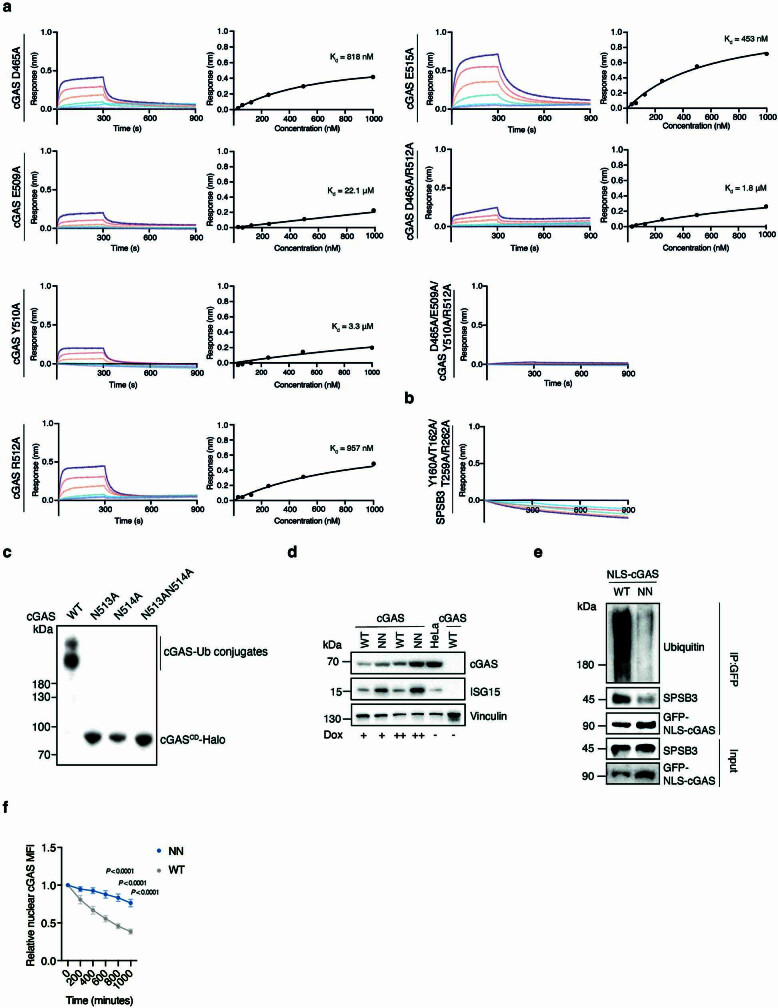
Effect of structure-guided mutations on cGAS binding to SPSB3. **a**, Left panels: bio-layer interferometry binding assays of His-Halo-tagged cGAS mutants with SPSB3-ELOBC. Right panels: binding affinity of cGAS mutants with SPSB3-ELOBC. **b**, Bio-layer interferometry binding assays His-Halo-tagged cGAS with an SPSB3 mutant in complex with ELOBC. **c**, Immunoblot showing in vitro ubiquitylation reactions of wild-type human cGAS catalytic domain (CD; aa: 155–522) or distinct cGAS CD mutants by CRL5^SPSB3^. **d**, Cell lysates from HeLa cells, HeLa cGAS KO cells reconstituted with doxycycline-inducible wild-type cGAS (WT), cGAS(NN) treated with different concentrations of doxycycline for 3 days were analysed by immunoblot. Vinculin was used as a loading control. **e**, IP of GFP-tagged cGAS from HEK293T cells transfected with constructs for GFP-tagged NLS-cGAS (WT) or GFP-tagged cGAS(NN), HA-tagged ubiquitin, and SPSB3. Samples were analysed by immunoblot. **f**, Relative nuclear cGAS-GFP MFI measurement in post-mitotic HeLa cGAS KO cells reconstituted with cGAS (WT) or cGAS(NN) (*n* = 15 cells per condition). Data are mean ± SD. Two-way ANOVA with Šídák’s multiple comparison test was used for statistical analysis. One representative of two (**d**-**e**) or three (**a**-**c**) independent experiments is shown. Source Data

We next combined the cGAS–SPSB3 complex structure with biochemical and cellular analysis to establish a molecular mechanism of nuclear cGAS control. Analysing the interaction using bio-layer interferometry (BLI), we observed that SPSB3 detects cGAS with submicromolar affinity (*K*_d_ = 352 nM) (Fig. 4a). Notably, cGAS single substitutions of either asparagine residue of the predicted interface (Asn513, Asn514) disrupted all detectable binding to SPSB3, demonstrating that the conserved NN motif on cGAS is the main contributor to SPSB3 recognition (Fig. 4a). Single point mutations of the supporting cGAS residues (Asp465, Glu509, Tyr510, Arg512), but not the adjacent position (Glu515), weakened the interaction with SPSB3, and a combined mutation of all four cGAS residues completely blocked binding, therefore confirming their importance in reinforcing complex formation (Extended Data Fig. 9a). Likewise, a combined substitution of cGAS-interacting residues on SPSB3 (Tyr160, Thr162, Thr259, Arg262) abrogated cGAS binding (Extended Data Fig. 9b). As expected from its central role in substrate receptor recruitment, mutation of the NN motif prevented cGAS ubiquitylation by CRL5–SPSB3 in vitro (Fig. 4b and Extended Data Fig. 9c). Cellular expression of cGAS or cGAS–NLS together with SPSB3 confirmed that cGAS(N513A/N514A) (hereafter cGAS(NN)) lost the ability to bind to SPSB3 and to undergo ubiquitylation (Fig. 4c, Extended Data Fig. 9d,e and Supplementary Video 5). Consistently, compared with WT cGAS, cGAS(NN) showed markedly increased protein abundance after reconstitution in HeLa *CGAS*-knockout (KO) cells, establishing the NN pair as a bona fide degron motif that dictates the nuclear stability of cGAS (Fig. 4d, Extended Data Fig. 9f and Supplementary Video 5).

## Nuclear cGAS impacts IFN tone

**Extended Data Fig. 10:**
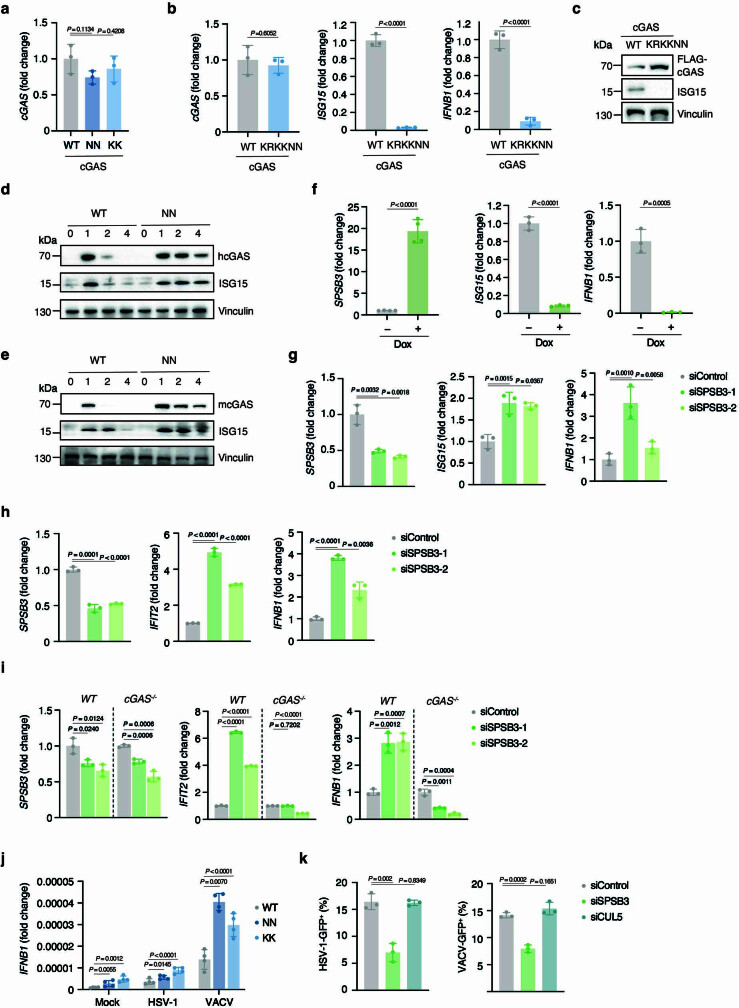
Disrupted nuclear cGAS ubiquitylation primes type I IFN signalling. **a**, mRNA levels of *cGAS* were measured by RT-qPCR in HeLa cGAS KO cells reconstituted with cGAS (WT), cGAS(NN), or cGAS(KK). Ratios relative to WT levels are shown (*n* = 3). **b**, Expression levels of *cGAS*, *ISG15* or *IFNB1* were assessed by RT-qPCR in HeLa cGAS KO cells reconstituted with doxycycline-inducible cGAS (WT) or a cGAS mutant defective in DNA binding and ubiquitylation (KRKKNN (K173E/R176E/K407E/K411A/N513/514A)) after 4 days of doxycycline treatment. Ratios of relative *cGAS*, *ISG15* and *IFNB1* mRNA levels normalized to the control are shown (*n* = 3). **c**, Cells from (**b**) were lysed and analysed by immunblot for cGAS and ISG15 levels. Vinculin was used as a loading control. **d**, cGAS and ISG15 measurements by immunoblot in HeLa cGAS KO cells transfected with mRNA of encoding for human *cGAS* or *cGAS N513A/N514A* (NN). Cells were harvested on day 0, 1, 2, or 4 after transfection. Vinculin was used as a loading control. **e**, cGAS and ISG15 measurements by immunoblot in CT26 cGas KO cells transfected with mRNA of encoding for mouse *cGas* or *cGAS N498A/N499A* (NN). Cells were harvested on day 0, 1, 2, or 4 after transfection. Vinculin was used as a loading control. **f**, Expression of *SPSB3*, *ISG15* or *IFNB1* were assessed by RT-qPCR 3 days after enforced expression or not of SPSB3 with doxycycline in HeLa cells. Ratios of relative *SPSB3*, *ISG15* and *IFNB1* mRNA levels normalized to the control are shown (*SPSB3*, *n* = 3; *ISG15*, *IFNB1*, *n* = 4). **g**-**i**, mRNA expression levels of genes as indicated were assessed by RT-qPCR in primary endothelial cells (*n* = 3) (**g**), BJ-5ta fibroblast cells (*n* = 3) (**h**), and differentiated THP-1 cells (*n* = 3) or THP-1 cGAS KO cells (*n* = 3) (**i**) treated with non-targeting control siRNAs or siRNAs against *SPSB3* for 5 days. Ratios of the relative expression of each transcript compared to the controls are shown. **j**, Induction of *IFNB1* mRNA was measured by RT-qPCR in HeLa cGAS KO cells reconstituted with cGAS (WT), cGAS(NN), or cGAS(KK) infected with HSV-1 (MOI = 0.1) or VACV (MOI = 1) for 18 h (*n* = 4). **k**, HeLa cells treated with non-targeting control siRNAs or siRNAs against *SPSB3* or *CUL5* for 5 days were infected with HSV-1-GFP (left, MOI = 0.01) or VACV-GFP (right, MOI = 0.5) for 24 h (*n* = 3). GFP^+^ cells were quantified by flow cytometry. Data are mean ± SD, numbers indicate the number of technical replicates (**a**-**b**, **f**-**k**). *P* values were obtained by one-way ANOVA (**a**, **f**, **g**-**k**) or two-tailed Student’s *t*-test (**b**, **f**). One representative of two (**a**) or three (**b**-**k**) independent experiments is shown. Source Data

To determine the biological consequences of nuclear cGAS control by CRL5–SPSB3, we next monitored cell-intrinsic type I interferon (IFN) responses, the most prominent cellular outcome of cGAS activity^34,35^. Reconstitution of *CGAS-*KO HeLa cells with SPSB3-binding-deficient cGAS(NN) or ubiquitylation-resistant cGAS(K427R/K428R) mutant (hereafter cGAS(KK)) induced increased levels of *IFNB1* and IFN-stimulated gene (ISG) expression relative to reconstitution with WT cGAS (Fig. 4d–f and Extended Data Fig. 10a). Additional mutation of residues involved in DNA binding^8,36^ abrogated the type I IFN response induced by the cGAS(NN) variant, demonstrating that cellular activation results from aberrant engagement of nuclear DNA (Extended Data Fig. 10b,c). Accordingly, expression of the degradation-deficient cGAS(NN) boosted the production of cGAMP, both in the context of WT cGAS and of a nucleosome-binding-deficient hyperactive cGAS mutant (R255A)^10^ (Fig. 4g). We transfected *CGAS*-KO cells with mRNAs allowing for transient expression of human CGAS or mouse cGas and observed markedly prolonged expression of nuclear cGAS(NN) (CGAS Asn498-Asn499 in mice) compared with WT cGAS (Extended Data Fig. 10d,e). Consistently, sustained expression of nuclear cGAS was paralleled by an enhanced type I IFN response in mRNA-treated cells (Extended Data Fig. 10d,e). Conversely, ectopic expression of *SPSB3* from a doxycycline-inducible promoter lowered baseline expression of ISGs in HeLa cells (Extended Data Fig. 10f). Using a complementary approach, we found that silencing of *SPSB3* elevated type I IFN signalling in various cell lines and this response was strictly dependent on cGAS (Fig. 4h and Extended Data Fig. 10g–i). To probe the relevance of heightened type I IFN signalling, we infected HeLa *CGAS*-KO cells reconstituted with either WT cGAS or degradation-defective cGAS variants at a low multiplicity of infection with the DNA viruses herpes simplex virus-1 (HSV-1) and vaccinia virus (VACV). In both cases, expression of degradation-defective cGAS mutants reduced the susceptibility of cells to infection and increased the expression of type I IFN (Fig. 4i and Extended Data Fig. 10j). Similarly, after depletion of *SPSB3*, cells displayed reduced viral infectivity compared with the controls (Extended Data Fig. 10k). By contrast, enforced degradation of cGAS by SPSB3 overexpression increased the susceptibility of the cells to infection with both HSV-1 and VACV (Fig. 4j). Together, these results demonstrate that nuclear cGAS levels affect the cellular IFN tone and reveal a role for CRL5–SPSB3 in cell-intrinsic immunity.

## Discussion

Along with previously defined interactions with nucleosomes, our results provide a complete structural model of the nuclear regulation of cGAS. Cytosolic exposure of chromatin in mitosis recruits cGAS onto nucleosomes, establishing the tightly chromatin-tethered nuclear cGAS pool. After mitotic exit, cGAS levels are tuned by SPSB3-directed ubiquitylation, resulting in low intranuclear protein abundance before the next mitosis. In contrast to a previous hypothesis, we do not find evidence for nuclear cGAS serving as a reservoir to replenish its corresponding cytosolic pool, at least under steady-state conditions^19^. Rather, we speculate that the presence of cGAS inside the nucleus is related to a nuclear function that is yet to be fully clarified. Coordination between nuclear cGAS levels and cell cycle activity might be necessary for balancing this function and the risk of autoreactivity, specifically before genome (DNA) duplication in the replicative phase of the cell cycle. The identified SPSB3-binding site on cGAS featuring the NN degron motif is distinct from previously described DNA- and protein-binding surfaces. Thus, in addition to the removal of nucleosome-bound cGAS, SPSB3 might target DNA-bound cGAS assemblies and free cGAS. This broad binding ability along with the irreversible nature of the degradation process engenders a highly potent mechanism of cGAS inactivation. The conservation of the NN degron, the key to the compatibility for SPSB3-directed protein removal, together with the conservation of the interfaces on SPSB3, points to a common mechanism in limiting the abundance of cGAS to tune cell-intrinsic innate immunity.

Our functional analysis demonstrates a relevance of levelling cGAS to maintain immune homeostasis. Disruption of the NN degron motif or depletion of SPSB3 boosted cGAS activity from within the nucleus resulting in priming of innate immunity. Conversely, enhanced SPSB3 activity suppressed tonic cGAS-dependent type I IFN responses. The CRL5–SPSB3-mediated checkpoint on cGAS, as defined in this study, may provide opportunities for the development of immune-targeted therapies that enhance or counteract cGAS–STING immunity in a given disease context. The structure of the cGAS–SPSB3 interface offers insights for the rational design of this type of cGAS-targeting pharmacology.

## Methods

### Cell culture

HeLa (CCL-2) cells were obtained from Sigma-Aldrich. HEK293T cells were a gift from D. Trono (EPFL), originally purchased from ATCC. U2OS cells, THP-1 cells and BJ-5ta cells were obtained from ATCC. CT26 mouse colorectal carcinoma cells were a gift from the D. Hanahan laboratory (EPFL). Primary human endothelial cells were obtained from a commercial supplier (Cell Biologics). HeLa and HEK293T cells were cultured in Dulbecco’s modified Eagle medium (DMEM, Thermo Fisher Scientific, 41965039) supplemented with 10% (v/v) heat-inactivated fetal bovine serum (FBS) (Thermo Fisher Scientific, Gibco, 10270106), 100 IU ml^−1^ penicillin–streptomycin (BioConcept, 4-01F00-H), 2 mM l-glutamine (Thermo Fisher Scientific, 25030024) and 1 mM sodium pyruvate (BioConcept, 5-60F00-H) at 37 °C under atmospheric O_2_ and 5% CO_2_. THP-1 cells were cultured in RPMI 1640 medium (Thermo Fisher Scientific, 21875091) supplemented with 10% FBS, 1× penicillin–streptomycin–l-glutamine (Corning, 30-009-Cl) and 1× 2-mercaptoethanol (Gibco) at 37 °C under atmospheric O_2_ and 5% CO_2_, and differentiated with 10 μg ml^−1^ phorbol-12-myristate-13-acetate (Sigma-Aldrich, P8139) for 2 days before the experiments. BJ-5ta cells were cultured in a mixed medium consisting of 80% DMEM (Thermo Fisher Scientific, 41965039) and 20% of medium 199 (Thermo Fisher Scientific, 41150087) supplemented with 10% FBS, 2 mM l-glutamine and 1× penicillin–streptomycin–l-glutamine. Primary human endothelial cells were cultured in complete human endothelial cell medium (Cell Biologics, H1168), according to the supplier’s instructions. CT26 cells were cultured in RPMI 1640 medium (Thermo Fisher Scientific, 21875091) supplemented with 10% FBS, 100 IU ml^−1^ penicillin–streptomycin (BioConcept, 4-01F00-H), 1 mM sodium pyruvate (BioConcept, 5-60F00-H) and 10 mM HEPES buffer (BioConcept, 5-31F00-H). *CGAS*-KO HeLa cells were reported previously^38^. U2OS *CGAS-*KO cells were generated as described previously^38^. *CGAS-*KO THP-1 cells were purchased from Invivogen. CT26 CGAS-KO cells were generated as described previously^38^.

HeLa cells, displaying a functional cGAS–STING pathway, were used for most of the experiments. U2OS cells, expressing cGAS, were used to independently validate hits obtained from the siRNA screen. HEK293T cells were used for immunoprecipitation and ubiquitylation studies using transiently expressed tagged constructs. BJ-5ta cells, THP-1 cells and primary endothelial cells were used to ascertain the relevance of cGAS nuclear degradation by the CRL5–SPSB3 ubiquitin ligase across distinct cell types and primary human cells. Cell lines were repeatedly tested for mycoplasma by PCR. No method of cell line authentication was used.

### Plasmids

For CRISPR–Cas9 plasmids, single guide RNAs (sgRNAs) targeting cGAS were designed using the web tool CRISPOR^39^. sgRNAs targeting cGAS were cloned into the pSpCas9(BB)-2A-GFP (PX458) plasmid (Addgene, 48138). pBABE-HA-ubiquitin was generated by inserting amplified HA-ubiquitin sequences from HA-ubiquitin (Addgene, 18712) into the pBABE vector. The pEFBos-cGAS-Flag, pEFBos-SPSB3-Flag and pEFBos-eGFP-Flag vectors were obtained by inserting cGAS, SPSB3 and eGFP sequences flanked by 5′ XhoI and 3′ NotI sites into the pEFBos vector. The pEFBos-based cGAS mutations (N513A/N514A, K427R/K428R and KRKKNN (K173E, R176E, K407E, K411A, N513A/N514A) and SPSB3 truncations (SPSB3ΔSOCS) were obtained by site-directed mutagenesis. The pTRIPZ-based cGAS and cGAS mutations (N513A/N514A, K427R/K428R and KRKKNN) were generated by inserting the coding sequences of cGAS flanked by 5′ XhoI and 3′ NotI sites into the pTRIPZ vector. The primers used for plasmid generation and sgRNA sequences are provided in Supplementary Table 2.

### Generation of cell lines

To establish HeLa cells or U2OS cells with inducible expression of distinct cGAS constructs, corresponding *CGAS*-KO cells were infected with a pTRIPZ lentiviral vector carrying cGAS-GFP or cGAS-Dendra2 and a puromycin-resistance gene. Cells were selected with puromycin (1 μg ml^−1^). To generate HeLa cGAS–GFP cells co-expressing PCNA–mCherry, HeLa cGAS–GFP cells were infected with a pRRL lentiviral vector (gift from D. Trono) carrying PCNA-mCherry and a blasticidin-resistance gene. Cells were selected using blasticidin (5 μg ml^−1^). HeLa cells expressing doxycycline-inducible Flag–cGAS, Flag–cGAS(NN), Flag–cGAS(KK) or Flag–cGAS(KRKKNN) were generated from HeLa *CGAS*-KO cells by infection with a pTRIPZ lentiviral vector carrying the corresponding inserts and a puromycin-resistance gene. Cells were selected with puromycin (1 μg ml^−1^).

### Transfection

For plasmid transfection, cells plated in a six-well plate were transfected with plasmids and GeneJuice transfection reagent (Millipore, 70967) according to the manufacturer’s protocol. For siRNA transfection, 3 × 10^4^ cells were transfected with Lipofectamine RNAiMAX transfection reagent (Invitrogen, 13778075) and 40 pmol siRNA according to the manufacturer’s protocol and incubated for 3–5 days. The medium containing transfection reagents was replaced with fresh medium 6 h after transfection. siRNA targeting *SPSB3* (s40519, s40520) and *CUL5* (s15588) and a negative control siRNA (4390847) were purchased from Thermo Fisher Scientific. Sequences of siRNAs are provided in Supplementary Table 2. For mRNA transfection, 2 × 10^5^ cells per well CT26 *Cgas*-KO mouse colorectal carcinoma cells or HeLa *CGAS*-KO cells were seeded into 12-well plates. Then, 500 ng of mRNA prediluted in Opti-MEM (Thermo Fisher Scientific) was mixed with 1.8 μl Lipofectamine RNAiMAX (Thermo Fisher Scientific; prediluted 1:20 in OptiMEM) for each well. After 15 min incubation at room temperature, mRNA mixture was added to cells. After 6 h, the medium was changed to remove mRNA. Cells were collected on day 0, 1, 2 or 4. mRNAs were generated by in vitro transcription at the mRNA platform of the University of Zürich (CH) as described previously^40^. Sequences of mRNAs are provided in Supplementary Table 2.

### RNAi screen targeting factors of the UPS

To identify regulators of nuclear cGAS stability, HeLa cells expressing doxycycline-inducible cGAS–GFP were treated with siRNAs targeting 972 UPS genes (three independent siRNAs per target) using the A30140 Silencer Select Human Ubiquitin siRNA library (Thermo Fisher Scientific) (Supplementary Table 1). Each plate (96-well PhenoPlate (PerkinElmer)) comprised 8 control wells, including 2 wells with negative control siRNA, 2 wells with a *cGAS*-targeting siRNA, and 4 wells containing epoxomicin (100 ng ml^−1^). For reverse transfection, 5 μl of siRNA (200 nM in Opti-MEM (Thermo Fisher Scientific)) was mixed with 5 μl Lipofectamine RNAiMAX (Thermo Fisher Scientific) (prediluted 1:20 in OptiMEM) inside each well. After 15 min incubation at room temperature, 3,500 cells were added to each well in 100 μl complete DMEM (10% FBS, 1% penicillin–streptomycin–glutamine solution, 1 μg ml^−1^ doxycycline). After 48 h, epoxomicin was added to the respective control wells. After incubation overnight (day 3 after reverse transfection), cells were washed with PBS and fixed by addition of a paraformaldehyde 4% solution in PBS supplemented with Hoechst (1 μg ml^−1^). Fixed cells were stored in PBS at 4 °C before imaging. Imaging of cells (entire well; 21 images at ×10 magnification) was performed using the IN Cell Analyzer 2200 microscope (GE Healthcare Life Sciences). Analysis of nuclear cGAS–GFP levels was performed using the CellProfiler software. In brief, each nucleus was identified by its Hoechst signal and intranuclear cGAS–GFP MFI and the integrated fluorescence intensity was quantified. The final results were obtained by calculating the relative values for MFI and integrated fluorescence intensity relative to the controls (*cGAS*-targeting siRNA = −1; negative control siRNA = 0; epoxomicin = 1) and data were visualized by blotting relative MFI levels against integrated fluorescence intensity levels. Genes were defined as hits if their relative fluorescence intensity values deviated from the negative control siRNA by more than 3 s.d.

### Live-cell imaging

HeLa cells expressing cGAS–GFP were plated in Falcon 96-well microplates (black; clear flat-bottom; tissue-culture-treated) (Corning) at a density of 3,500 to 7,500 cells per well in complete FluoroBrite (Thermo Fisher Scientific) DMEM (10% FBS, 2 mM l-glutamine, 1% penicillin–streptomycin and 1 μg ml^−1^ doxycycline). The next day, cells were transfected or not with siRNAs and incubated for 2 days. Drug treatment, including epoxomicin (200 nM), bortezomib (100 nM) and leptomycin B (80 nM), was performed 1 h before live-cell recording. Live-cell imaging of cGAS(WT)–GFP was performed using a confocal Leica SP8 Inverted microscope equipped with an HC PL APO ×63/1.40 NA oil-immersion objective and HyD detectors, operated with the Leica LAS X software. For experiments with cGAS(WT)–GFP and cGAS(NN) or cGAS–Dendra2, live-cell imaging was performed on the Zeiss LSM980 Inverted microscope (Carl Zeiss) equipped with a motorized, heated stage and a full incubation chamber maintaining 37 °C and 5% CO_2_ and operated with the Zeiss ZEN software. Images were acquired every 3–5 min. Image analysis was performed using Fiji (v.2.3.0) on a nucleus-by-nucleus basis and data were further quantified using GraphPad PRISM 9 (v.9.3.1). In experiments using cGAS–Dendra2, Dendra2 was photoactivated before live-cell imaging using the 405 nm laser (700 Hz; laser power: 7.5%) to scan the same region of interest four times (frame average). For cell cycle phase analysis, a single cell nucleus was tracked over time using PCNA–mCherry as a mask for the nucleus, while recording the cGAS–GFP nuclear intensity. A change in PCNA–mCherry signal from a diffuse pattern to a dot-like pattern was used to define the transition from G1 to S phase of the cell cycle, whereas the reverse change was used to define the transition from S to G2 phase of the cell cycle.

### Immunofluorescence and confocal imaging

Cells were plated in CellCarrier-96 Ultra Microplates (Perkin Elmer, 6055302) at a density of 10,000 cells per well with at least 5 h incubation for adherence of the cells. For immunofluorescence, cells were washed once with PBS, then cells were fixed by adding paraformaldehyde 4% in CBS buffer (10 mM MES pH 6.9, 138 mM KCl, 2 mM MgCl_2_, 2 mM EGTA) for 5–10 min at room temperature. Cells were washed three times for at least 5 min in PBS before blocking for 1–2 h at room temperature with PBS supplemented with 0.1% (v/v) Triton X-100 and 5% (v/v) heat-inactivated FBS and incubating overnight at 4 °C with the primary antibodies diluted in staining solution (PBS supplemented with 0.1% (v/v) Triton X-100) and 1% (w/v) bovine serum albumin (Sigma-Aldrich, A7906)). A list of the antibodies and the concentrations used is provided in Supplementary Table 2. The cells were washed with PBS three times for 5 min and incubated for 1 h at room temperature in secondary antibodies diluted in staining solution. From then on, the plate was protected from light. Cells were washed twice (5 min each) in PBS and incubated for 30–60 min in Hoechst 33342 (Sigma-Aldrich, B2261) 0.2 μg ml^−1^ in PBS. Cells were kept in 100 μl PBS per well and either imaged directly or kept at 4 °C until imaging. Imaging was performed using the Zeiss LSM 980 Inverted microscope (Carl Zeiss) using the Plan-Apochromat ×63/1.40 NA oil-immersion objective. Image analysis and quantifications were performed using Fiji (v.2.3.0).

### Quantification of cGAMP levels by ELISA

cGAMP levels were quantified as previously described^15^. In brief, cells were collected by trypsination (trypsin-EDTA (0.05%), Life Technologies). Cell pellets were lysed in RIPA lysis buffer containing 50 mM Tris, 150 mM NaCl, 1% (w/v) sodium deoxycholate, 0.03% (v/v) SDS, 0.005% (v/v) Triton X-100, 5 mM EDTA, 2 mM sodium orthovanadate and cOmplete Protease Inhibitor Cocktail (Roche). Lysed cells were centrifuged for 10 min at 18,200 *g* and 4 °C. The supernatant was used for the cGAMP ELISA assay (Cayman 2′3′-cGAMP ELISA kit, 501700) according to the manufacturer’s instructions. The protein concentration in the supernatant was measured using the BCA Pierce Protein assay kit and was used to normalize cGAMP levels.

### QIBC and analysis

Cells were plated in CellCarrier-96 Ultra Microplates (Perkin Elmer, 6055302) at a density of 10,000 cells per well with 24 h incubation for adherence of the cells. For experiments with cGAS–GFP cells, 1 μg ml^−1^ doxycycline was added for 24 h before fixation. For experiments with drug treatment, epoxomicin (200 nM) or MLN4924 (1 μM) was added for 16 h or 8 h before fixation. For experiments with siRNA knockdown, cells were transfected with Lipofectamine RNAiMAX transfection reagent (Invitrogen, 13778075) according to the manufacturer’s protocol followed by 5 days of incubation before fixation. To define the population of cells in a different cell cycle phase, EdU and DNA were fluorescently labelled using the Click-IT EdU Imaging Kit with Alexa Fluor 594 Azide (Invitrogen, C10086) and DAPI according to the manufacturer’s instructions. Cells were then stained with primary and secondary antibodies as described before.

High-content images were acquired on the fully automated Operetta CLS (PerkinElmer) system at ×20 operated with Harmony (PerkinElmer) software. Analysis of fluorescence intensities was performed using CellProfiler software. Nuclear intensity measurements were determined within a nuclear mask generated from DAPI staining. Cytoplasmic intensity measurements were determined within a ringed mask outside the nucleus and inside the cell border generated from the digital phase contrast channel. The background was determined by LowerQuartileIntensity and subtracted from the mean intensity of the given fluorescent marker. Colour-coded scatter plots were generated using KNIME v.4.7.4 (KNIME) with custom scripts. The DNA content was determined using the integrated intensity of DAPI signal. The S phase cell population was determined by the mean intensity of EdU signal. The G2/M phase cell population was determined by the DNA content and the M phase population was then extracted by the mean intensity of DAPI signal, which was further confirmed by anti-H3pS10 staining. Finally, the intensities of different populations were collected and analysed using GraphPad PRISM 9 (v.9.3.1).

### Antibodies

The following primary antibodies were used: mouse monoclonal anti-vinculin (hVIN-1; Sigma-Aldrich, V9264, immunoblot, 1:5,000), rabbit monoclonal anti-GAPDH (14C10; Cell Signaling Technology, 2118, immunoblot, 1:3,000), mouse monoclonal anti-Flag (M2; Sigma-Aldrich, F1804, immunoblot, 1:5,000), rabbit monoclonal anti-ISG15 (EPR24482-49; Abcam, ab285367, immunoblot, 1:1,000), rabbit polyclonal anti-ISG15 (Cell Signaling Technology, 2743S, immunoblot, 1:1,000), rabbit polyclonal anti-cGAS (Novus, NBP3-16666, immunoprecipitation, 1:50), rabbit monoclonal anti-cGAS (D3O8O; Cell Signaling Technology, 31659, immunoblot, 1:1,000), rabbit monoclonal anti-cGAS (D1D3G; Cell Signaling Technology, 15102, immunoblot, 1:1,000), rabbit monoclonal anti-cGAS (E5V3W; Cell Signaling Technology, 79978, immunoblot, 1:1,000; immunofluorescence, 1:200), mouse monoclonal anti-ubiquitin (P4D1; Santa Cruz, sc-8017, 1:500), rabbit monoclonal anti-ubiquitin (linage-specific K48; EP8589; Abcam ab140601, immunoblot, 1:200), rabbit polyclonal anti-SPSB3 (Aviva, ARP71676_P050, immunofluorescence 1:100, immunoblot, 1:1,000), rabbit polyclonal anti-H3pS10 (Sigma-Aldrich, 06-570, immunofluorescence 1:200), rabbit polyclonal anti-cullin-5 (Abcam, ab264284, immunofluorescence 1:100), mouse monoclonal anti-His-tag (27E8; Cell Signaling Technology, 2366, immunoblot, 1:1,000), rabbit monoclonal anti-H2B (Abcam, 52484, immunoblot, 1:5,000). The following HRP-conjugated secondary antibodies were used: donkey anti-rabbit IgG (H+L)-HRP (Jackson ImmunoResearch, 711-036-152, immunoblot: 1:5,000) and donkey anti-mouse IgG (H+L)-HRP (Jackson ImmunoResearch, 715-036-151, immunoblot: 1:5,000). The following fluorescence-conjugated secondary antibodies were used: goat anti-rabbit IgG (H+L) cross-adsorbed secondary antibody, Alexa Fluor 568-conjugated (Invitrogen, A-11011, immunofluorescence 1:800), goat anti-rabbit IgG (H+L) cross-adsorbed secondary antibody, Alexa Fluor 488-conjugated (Invitrogen, A-11008, immunofluorescence 1:800), Click-iT EdU Imaging Kit with Alexa Fluor 647 (Thermo Fisher Scientific, C10086). A list of antibodies is provided in Supplementary Table 2.

### RT–qPCR analysis

Cells were lysed in the RLT buffer (Qiagen). RNA was extracted according to the manufacturer’s protocol (Qiagen RNeasy Mini Kit). RNA was reverse transcribed using PrimeScript RT Reagent Kit (Takara) and analysed by RT–qPCR in triplicates using the Maxima SYBR Green/ROX qPCR Master Mix (Thermo Fisher Scientific). The qPCR reactions were run on the QuantStudio 7 Real-Time PCR system (Thermo Fisher Scientific). *GAPDH* was used as the housekeeping gene for normalization. Primer sequences are provided in Supplementary Table 2.

### Cell fractionation

Cells from a 10 cm^2^ dish were lysed with cold 400 μl buffer A (10 mM HEPES pH 7.9, 10 mM KCl, 1.5 mM MgCl_2_, 0.34 M sucrose, 10% glycerol, 1 mM DTT, 20% Triton X-100) on ice for 5 min. Cytosolic fractions were collected by centrifugation at 3,500 rpm, 4 °C for 5 min. Pellets were washed twice with buffer A before being resuspended with 400 μl buffer B (3 mM EDTA, 0.2 mM EGTA) on a rotator at 4 °C for 30 min. The soluble nuclear fractions were collected by centrifugation at 4,000 rpm, 4 °C for 5 min. The chromatin pellets were then washed with buffer B twice until they became almost invisible before being lysed with salt buffer (50 mM Tris pH 7.5, 300 mM NaCl, 1% Triton X-100, 1 mM DTT) and boiled with 1× loading buffer for 20 min.

### Immunoblotting and immunoprecipitation

Cells were collected, quickly rinsed with 1× PBS and lysed in lysis buffer (20 mM Tris pH 7.4, 0.5% Triton X-100, 150 mM NaCl, 1.5 mM MgCl_2_, 2 mM EGTA, 2 mM DTT and 1× cOmplete Protease Inhibitor Cocktail (Roche)) on ice for 30 min and centrifuged at 12,000 rpm, 4 °C for 10 min. The supernatants were boiled with 4× loading buffer (200 mM Tris pH 6.8, 8% SDS, 40% glycerol, 0.4 M DTT, 0.4% bromophenol blue) for 10 min. Proteins were resolved by SDS–PAGE using SurePAGE precast gels (GenScript) and transferred to nitrocellulose membranes using the Trans-Blot Turbo RTA Midi Nitrocellulose Transfer Kit (Bio-Rad) according to the manufacturer’s instructions. Membranes were blocked with 3% skimmed milk in PBST (PBS with 0.05% Tween-20) at room temperature for 1 h and then incubated with the primary antibody (diluted in PBST) at 4 °C overnight. After washing in PBST, membranes were incubated with the secondary antibody at room temperature for 1 h. Membranes were washed with PBST, visualized with western blotting detection reagent (Bio-Rad) and imaged using the ChemiDoc XRS Bio-Rad Imager and Image Lab Software.

For immunoprecipitation, cells were seeded into six-well plates and were transfected with the indicated plasmids. Then, 16 h after transfection, cells were lysed in IP lysis buffer (PBS supplemented with 1% TEGITOL solution, 0.5% sodium deoxycholate, 1× cOmplete Protease Inhibitor Cocktail (Roche) and 1 μl benzonase) on ice for 30 min and centrifuged at 12,000 rpm 4 °C for 10 min. The supernatants were transferred into new tubes and mixed with anti-Flag M2 magnetic beads (Sigma-Aldrich, M8823) or ChromoTek GFP-Trap agarose (Proteintech) at 4 °C overnight on a rotator. After 3–6 washes with IP wash buffer (PBS supplemented with 300 mM NaCl), beads or agaroses were boiled with 1× loading buffer for 10 min. Gels were loaded with 20 μl samples, and then analysed using SDS–PAGE and immunoblotting. Full scans of the immunoblots are shown in Supplementary Fig. 1.

### Cycloheximide chase experiments

After treatment of cGAS–GFP-expressing HeLa cells with 500 nM MLN4924 for 1 h or siSPSB3/siControl for 48 h, cells were treated with cycloheximide (40 μg ml^−1^) for 0, 2, 4 and 6 h before collection. Cells were fractionated using the commercial subcellular protein fractionation kit (Thermo Fisher Scientific, 78840). The fractionated samples were diluted in Laemmli buffer and boiled for 20 min at 98 °C. Expression of the indicated proteins was determined using immunoblotting.

### Protein expression and purification

Halo-tagged human cGAS WT, Halo-tagged cGAS catalytic domain (amino acids 155–522) and the corresponding substitutions in the pET-28 vector were expressed in BL21 (DE3) bacteria (Sigma-Aldrich, CMC0014). A single colony was inoculated in a culture flask with 100 ml LB with kanamycin (50 μg ml^−1^) and incubated with shaking (200 rpm, Infors-HT Multitron) at 37 °C overnight as preculture. Large-scale expression of the protein was started the next day by pouring 100 ml of the preculture in a 5 l Erlenmeyer flask containing 2 l LB with kanamycin (50 μg ml^−1^). The cells were grown until the optical density at 600 nm reached 0.7. Expression was then induced by adding isopropyl β-d-1-thiogalactopyranoside (IPTG) to a final concentration of 0.5 mM while transferring the culture to an 18 °C shaking incubator overnight. Bacteria were collected by centrifugation, solubilized in HisTrap buffer A (20 mM HEPES, 500 mM NaCl, 20 M imidazole, 1 mM DTT and 5% glycerol, pH 7.5) supplemented with 4-(2-aminoethyl)-benzolsulfonylfluoride-hydrochloride (AEBSF) and cOmplete protease inhibitor (Roche), lysed by sonication, cleared by centrifugation at 20,000*g* and then passed through a 5 ml nickel-immobilized metal-affinity chromatography column (Cytiva, HisTrap HP, 17524802) on an fast protein liquid chromatography (FPLC) system. The protein of interest was eluted with buffer B (20 mM HEPES, 500 mM NaCl, 500 mM imidazole, 1 mM DTT and 5% glycerol, pH 7.5). Halo-tagged cGAS was then purified by SEC through the Superdex 75 Hiload 16/600 column (Cytiva 28-9893-33). For untagged cGAS, TEV enzyme was mixed with Halo-tagged cGAS overnight, and untagged cGAS was then purified by SEC through the Superdex 75 Hiload 16/600 column (Cytiva 28-9893-33). Halo-tagged mouse cGAS was prepared using the same procedure. All mutants were generated using a PCR-based technique with appropriate primers and confirmed by DNA sequencing.

The cDNA of human *SPSB3* (amino acids 83–326) or its corresponding mutant was cloned into a pET-28 vector with an N-terminal His6-SUMO tag. SPSB3 was expressed in BL21 (DE3) (Sigma-Aldrich, CMC0014). A single colony was inoculated into a culture flask with 400 ml LB with kanamycin (50 μg ml^−1^) and incubated with shaking (200 rpm, Infors-HT Multitron) at 37 °C overnight as preculture. Large-scale expression of the complex (8 l in total: 2 l in four 5 l Erlenmeyer flasks) was started the next day by pouring 100 ml of preculture into 2 l of Auto Induction Media Terrific Broth (Formedium, AIMTB0210) with kanamycin (50 μg ml^−1^). The flasks were incubated with shaking at 37 °C for 6 h, then incubated at 18 °C overnight. The cells were then collected by centrifugation (4,000*g*, 15 min). The cell pellet of a 2 l expression culture was transferred into a Falcon 50 ml tube. The 2 l expression cells were solubilized in PBS with 1 mM DTT, 1 mM EDTA and 2% glycerol at pH 7.5, supplemented with AEBSF and cOmplete protease inhibitors (Roche), then lysed by sonication. The cell lysate was clarified by centrifugation followed by 0.45 μm filtration. The supernatant was first purified on a 5 ml nickel-immobilized metal-affinity chromatography column (Cytiva, HisTrap HP, 17524802) on a FPLC system (Cytiva Aktä Pure). ULP1 cleavage was performed at 4 °C overnight in a 3,500-Da molecular weight cut-off dialysis tube against PBS with 5% glycerol at pH 7.5. To remove the ULP1 protease as well as free SUMO, the sample was passed through a HisTrap and washed with PBS with 20 mM Imidazole. The flowthrough was collected and concentrated using the Superose 6 HiLoad 16/600 SEC (Cytiva, 29323952) equilibrated in PBS. All mutants were generated using a PCR-based technique with appropriate primers and confirmed by DNA sequencing.

Recombinant proteins required for in vitro ubiquitylation assays were expressed in BL21(DE3) cells, except for UBE1 and the neddylated CUL5–RBX2 complex (purchased from Bio-Techne, E-305-025 and E3-451-025). In brief, the human *UBE2R1* (UniProt: P49427) coding sequence was cloned into the pET-28-His-thrombin vector to enable expression as an N-terminal His-thrombin fusion protein; the human *UBE2L3* (UniProt: P68036) coding sequence was cloned into the pGEX-4T-1 vector to enable expression as an N-terminal GST-fusion protein; the human *ARIH2* (UniProt: O95376) coding sequence was cloned into the pGEX-4T-1 vector to enable expression as an N-terminal GST-fusion protein. After transformation of BL21(DE3) competent cells, protein expression (UBE2R1, UBE2L3, ARIH2) was induced by addition 1 mM IPTG when the optical density at 600 nm reached 0.6, and bacteria were cultured at 18 °C overnight. For His-tagged proteins, cell pellets were collected and suspended in lysis buffer (20 mM imidazole, 50 mM Tris pH 8.0, 300 mM NaCl, 5% glycerol, 5 mM BME) supplemented with cOmplete protease inhibitors (Roche). After sonication, the cell lysates were clarified by centrifugation at 20,000*g* for 30 min. The supernatants were collected and incubated with Ni-NTA agarose at 4 °C for 2 h. Resins were washed and bound protein was eluted by 250 mM imidazole in buffer (50 mM Tris pH 8.0, 300 or 400 mM NaCl, 5% glycerol, 5 mM BME). To remove tags, TEV protease or thrombin was added to the eluates and untagged proteins were further purified by gel filtration with Superdex 75 increase 10/300 column (Cytiva, 29148721) equilibrated with buffer (25 mM Tris pH 8.0, 200 mM NaCl, 5% glycerol, 1 mM DTT). Protein purify was checked by SDS–PAGE electrophoresis and pure fractions were pooled, flash-frozen in liquid nitrogen and stored at −80 °C until further use.

For GST-tagged proteins, cell pellets were collected and suspended in lysis buffer (50 mM Tris pH 8.0, 300 mM NaCl, 5% glycerol, 5 mM BME) supplemented with cOmplete protease inhibitors (Roche). After sonication, the cell lysates were clarified by centrifugation at 20,000*g* for 30 min. The supernatants were collected and incubated with Glutathione Sepharose Resins followed by washing, and the bound protein was eluted using 15 mM GSH in lysis buffer. Removal of GST tag was facilitated by adding thrombin to the eluates and the untagged protein was further purified by a second Glutathione Sepharose column followed by gel filtration with the Superdex 75 increase 10/300 column equilibrated with 25 mM Tris pH 8.0, 200 mM NaCl, 5% glycerol and 1 mM DTT. The fractions containing pure protein were pooled, flash-frozen in liquid nitrogen and stored at −80 °C until further use.

### BLI analysis

BLI analyses were performed at 25 °C using the GatorPrime biosensor system (GatorBio) with anti-His probes (GatorBio, 160009). cGAS or cGAS mutants (50 μg ml^−1^) were immobilized onto the anti-His biosensor for 1 min. The tips were washed with PBS buffer for 2 min to obtain a baseline reading, then the biosensors were dipped into wells containing the various concentrations of SPSB3 or its mutant for 5 min. Then, a 10 min buffer wash was performed to allow the dissociation of molecules from the sensor. Data analysis was performed with GraphPad PRISM 9 using a standard 1:1 binding model. Three independent experiments were performed for each sample.

### Cryo-EM data acquisition

A total of 1 mg cGAS(WT)-Halo-SPSB3-ELOBC or 1 mg cGAS(site C)-Halo-SPSB3-ELOBC complex was incubated with 1 mg recombinant mononucleosome core particle (Active motif, 81770) for 30 min on ice in PBS. Fractionations corresponding to the complex were collected on the Supedex 200 increase 10/300 GL column (Cytiva) in PBS and concentrated to 1 mg ml^−1^. Aliquots of 3 μl of the cGAS–Halo–SPSB3–ELOBC–nucleosome complexes were loaded onto glow-discharged holey carbon grids (Electron Microscopy Sciences, CF-2/2-3Cu-50, C-Flat, Cu, R 2/2, 300 mesh). Grids were blotted for 4 s and plunge-frozen in liquid ethane using the Vitrobot at 4 °C with 100% humidity. The grids were screened for particle presence and ice quality on a the TFS Glacios microscope (200 kV), and the grids with the best quality were transferred to a TFS Titan Krios G4. Cryo-EM data were collected using the TFS Titan Krios G4 transmission electron microscope (TEM), equipped with a Cold-FEG on the Falcon IV detector in electron-counting mode. Falcon IV gain references were collected just before data collection. Data were collected with TFS EPU v.2.12.1 using the aberration-free image shift protocol (AFIS), recording eight micrographs per ice hole. Videos were recorded at ×96,000 magnification, corresponding to a pixel size of 0.83 Å at the specimen level, with defocus values ranging from −0.8 to −1.8 μm. Exposures were adjusted automatically to 60 e^−^ Å^−2^ total dose, resulting in an exposure time of approximately 5 s per video. In total, 9,855 micrographs in EER format were collected for the cGAS WT complex and 15,141 micrographs in EER format were collected for the cGAS(site C) sample.

### Cryo-EM data processing

Motion correction was performed on raw stacks without binning using the cryoSPARC implementation of motion correction. For the cGAS(WT)–Halo–SPSB3–ELOBC–nucleosome complex, a total of 972,457 particles was template-based automatically picked and particles were binned by a factor of 4. Two rounds of two-dimensional (2D) classification were performed, resulting in a particle set of 378,280 particles. Selected particles resulting from the 2D classification were used for ab initio reconstruction with two classes, and the one containing the nucleosome shape was selected as the initial model. A hetero-refinement was performed on three repeating initial models on the resulting 2D particles. A class with clear cGAS density was selected, including 69,435 particles. To deal with the sample heterogeneity, a mask on a single nucleosome and cGAS–SPSB3–ELOBC was generated for masked 3D-variability analysis with three modes. A mode showing variability on SPSB3 was selected for clustering with ten classes, among which, four classes containing SPSB3–ELOBC density were selected and re-centred and re-extracted. The particles were processed for iterative CTF refinement and non-uniform refinement in cryoSPARC to 4.30 Å. The reported resolutions are based on the gold-standard Fourier shell correlation (FSC) 0.143 criterion. For the cGAS(site C)–Halo–SPSB3–ELOBC–nucleosome complex, a total of 5,257,474 particles was template-based automatically picked and particles were binned by a factor of 4. Two rounds of 2D classification were performed, resulting in a particle set of 1,400,892 particles. Selected particles resulting from the 2D classification were used for ab initio reconstruction with two classes, and the one containing the nucleosome shape was selected as the initial model, including 1,183,961 particles. A non-uniform refinement refines the map to 7.03 Å. To deal with the sample heterogeneity, a mask on the single nucleosome and cGAS–SPSB3–ELOBC was generated for masked 3D-variability analysis with three modes. A mode showing variability on SPSB3 was selected for clustering with 20 classes, among which, 9 classes containing SPSB3 density were selected. Then, a mask on cGAS–SPSB3–ELOBC was generated and used for local refinement, followed by a masked 3D-variability analysis with three modes with the same mask. A mode showing variability on ELOBC was selected for clustering with 20 classes, among which, seven classes containing ELOBC density were selected, re-centred and re-extracted. The particles were processed for iterative CTF refinement and non-uniform refinement in cryoSPARC to a resolution of 2.75 Å. A local refinement on cGAS–SPSB3–ELOBC refined the map to 3.51 Å. The reported resolutions are based on the gold-standard FSC 0.143 criterion. Local-resolution variations were estimated using cryoSPARC^41^.

### Model building and refinement

The nucleosome–cGAS–SPSB3–ELOBC model was generated using a published nucleosome cGAS structure (PDB: 6X59 (ref. ^11^), 6Y5E (ref. ^10^) and 4LEV (ref. ^42^)). Direct prediction of the cGAS–SPSB3–ELOBC complex failed to find SPSB3 interacting with cGAS by Alphafold2. The SPSB3–ELOBC model from AlphaFold2 was docked into the cryo-EM map in ChimeraX^37^ and tuned by ISOLDE^43^ and Coot^44^. The whole model was then refined in PHENIX^45^. Several loop regions of cGAS and SPSB3 were manually adjusted to fit into the map using Coot. The model was refined in real space again in PHENIX. All structure figures were made using UCSF Chimera^46^ and UCSF ChimeraX^37^.

### Sequence alignments

A total of 150 human cGAS/SPSB3 orthologues in vertebrates were individually aligned and downloaded in ClustalW format from the Ensembl database. The sequence logo was derived from WebLogo (https://weblogo.berkeley.edu) on the indicated alignment regions. The human SPSB1/2/3/4 sequence as well as their percentage identity matrix analysis were downloaded from UniProt. Geneious Prime software was used to generate the sequence alignments shown in Extended Data Figs. 5d and  8c.

### In vitro ubiquitylation assays

Human cGAS ubiquitylation assay was performed at 37 °C for 40 min. Reactions (20 μl) consisted of 50 μM ubiquitin, 100 nM UBE1, 1 μM UBE2R1, 0.35 μM CUL5–RBX2–NEDD8 complex, 0.7 μM SPSB3–ELOBC complex and 1 μM cGAS or cGAS mutants in buffer (50 mM Tris-HCl pH 7.5, 50 mM NaCl, 2.5 mM MgCl_2_, 1 mM ATP, 2 mM DTT). If needed, 1 μM UBE2L3 and 300 nM ARIH2 were also added to the reaction. The reactions were initiated by ATP and quenched with 5× SDS–PAGE loading buffer after 40 min. Ubiquitylated cGAS products were separated on 4–20% SurePAGE (GenScript) and were probed with cGAS monoclonal antibodies (Cell Signaling, E5V3W). The ubiquitylation assay for mouse cGAS was slightly modified: 200 nM UBE1, 2 μM UBE2L3 and 600 nM ARIH2 were added to the reaction and the incubation time was extended to 3 h at 37 °C. Ubiquitylated mouse cGAS products were probed with His-tag monoclonal antibodies (Cell Signaling, 27E8).

### Identification of ubiquitylation sites on cGAS

To map lysine residues on cGAS modified by ubiquitin, in vitro ubiquitylation reactions were performed in the presence of UBE2L3 and ARIH2. After SDS–PAGE electrophoresis, the gel was stained with Coomassie Blue and putative ubiquitylated cGAS bands were excised for MS analysis. MS-based proteomic experiments were performed at the Proteomics Core Facility of the School of Life Sciences at EPFL. The selected gel pieces were excised and washed twice with 50% ethanol in 50 mM ammonium bicarbonate (Sigma-Aldrich) for 20 min and dried by vacuum centrifugation. Proteins were reduced with 10 mM dithioerythritol (Merck-Millipore) for 1 h at 56 °C followed by a washing–drying step as described above. Reduced proteins were alkylated with 55 mM iodoacetamide (Sigma-Aldrich) for 45 min at 37 °C in the dark followed by a washing–drying step as described above. Proteins were digested overnight at 37 °C using MS-grade Trypsin Gold (Promega) at a concentration of 12.5 ng μl^−1^ in 50 mM ammonium bicarbonate supplemented with 10 mM CaCl_2_. The resulting peptides were extracted in 70% ethanol, 5% formic acid (Merck-Millipore) twice for 20 min, dried by vacuum centrifugation and stored at −20 °C until further analysis.

### LC–MS/MS analysis

Peptides were desalted on C18 StageTips and dried by vacuum centrifugation before liquid chromatography coupled with tandem MS (LC–MS/MS) injections^47^. The samples were resuspended in 2% acetonitrile (Biosolve), 0.1% formic acid and nano-flow separations were performed on the Dionex Ultimate 3000 RSLC nano UPLC system (Thermo Fischer Scientific) online connected with an Q Exactive Orbitrap Mass Spectrometer (Thermo Fischer Scientific). A capillary precolumn (Acclaim Pepmap C18, 3 μm, 100 Å, 2 cm × 75 μm inner diameter) was used for sample trapping and cleaning. A 50 cm long capillary column (75 μm inner diameter; in-house packed using ReproSil-Pur C18-AQ 1.9 μm silica beads) was then used for analytical separations at 250 nl min^−1^ over 90 min biphasic gradients. The mobile phases were as follows: A, 2% acetonitrile, 0.1% formic acid in water; and B, 90:10 (v/v) acetonitrile:water, 0.1% formic acid. The mass spectrometer was operated in positive data-dependent acquisition mode, and the full MS range was from 300 to 2,000 *m*/*z*. The 12 most intense ions were isolated in the quadrupole and fragmented under high-energy collisional dissociation with a normalized collision energy of 27% with a 30 s exclusion list. Precursor and fragment ions were measured at a resolution of 70,000 and 17,500 (at 200 m/z), respectively. Only ions with charge states of 2 and higher were fragmented with an isolation window of 1.2 *m*/*z*.

Raw data were processed using SEQUEST, MS Amanda^48^ and MS Fragger^49^ in Proteome Discoverer 2.5 against the provided sequences on a *Homo sapiens* proteome background. Enzyme specificity was set to trypsin and a minimum of six amino acids was required for peptide identification. Up to two missed cleavages were allowed and a 1% FDR cut-off was applied both at the peptide and protein identification levels. For the database search, carbamidomethylation was set as a fixed modification, whereas oxidation (M), acetylation (protein N-term), PyroGlu (N-term Q) and phosphorylation (S, T, Y) and K-GG were considered as variable modifications. Data were further processed and inspected in Scaffold v.5.1.0 (Proteome Software).

### Virus infection experiments

GFP-expressing virus strains were a gift from F. Schmidt and were described previously (VACV-GFP; ref. ^50^) or were generated as described previously^51^. HSV-1 (KOS) and VACV were obtained from ATCC. HeLa *CGAS*-KO cells were treated with doxycycline for 4 days to induce cGAS, cGAS(NN) or cGAS(KK) expression, or HeLa cells were treated with doxycycline for 2 days to trigger enforced expression of SPSB3. Infections were performed by incubating virus inoculum (multiplicity of infection, 0.1) with cells for 3 h, before the cells were washed and cultured in complete DMEM (10% FBS, 1% penicillin–streptomycin–glutamine solution). After overnight incubation, cells were fixed and GFP^+^ cells were analysed by flow cytometry. The flow cytometry gating strategy is shown in Supplementary Fig. 2. To assess mRNA expression levels of *IFNB1*, cells were infected with HSV-1 (KOS) (multiplicity of infection, 0.1) or VACV (multiplicity of infection, 1) for 18 h.

### Reporting summary

Further information on research design is available in the Nature Portfolio Reporting Summary linked to this article.

## Online content

Any methods, additional references, Nature Portfolio reporting summaries, source data, extended data, supplementary information, acknowledgements, peer review information; details of author contributions and competing interests; and statements of data and code availability are available at 10.1038/s41586-024-07112-w.

### Supplementary information

Supplementary Figures Supplementary Figs. 1 and 2.

Reporting Summary

Supplementary Note 1 Additional information about the purification of the SPSB3–ELOBC complex.

Supplementary Table 1 Raw data siRNA screen.

Supplementary Table 2 List of primer sequences, siRNA sequences and antibodies used in this study.

Supplementary Data Mass SPSB3 and KanR source data.

Supplementary Video 1 Live-cell imaging of cGAS–GFP in HeLa cells.

Supplementary Video 2 Nuclear cGAS degradation in distinct cell cycle phase.

Supplementary Video 3 Epoxomicin inhibits nuclear cGAS degradation.

Supplementary Video 4 Epoxomicin inhibits nuclear cGAS degradation.

Supplementary Video 5 Mutation of the N513/4A cGAS degron motif blocks intranuclear cGAS degradation.

### Source data

Source Data Fig. 1

Source Data Fig. 4

Source Data Extended Data Fig. 1

Source Data Extended Data Fig. 2

Source Data Extended Data Fig. 3

Source Data Extended Data Fig. 4

Source Data Extended Data Fig. 5

Source Data Extended Data Fig. 6

Source Data Extended Data Fig. 7

Source Data Extended Data Fig. 9

Source Data Extended Data Fig. 10

## Figures and Tables

**Extended Data Table 1:**
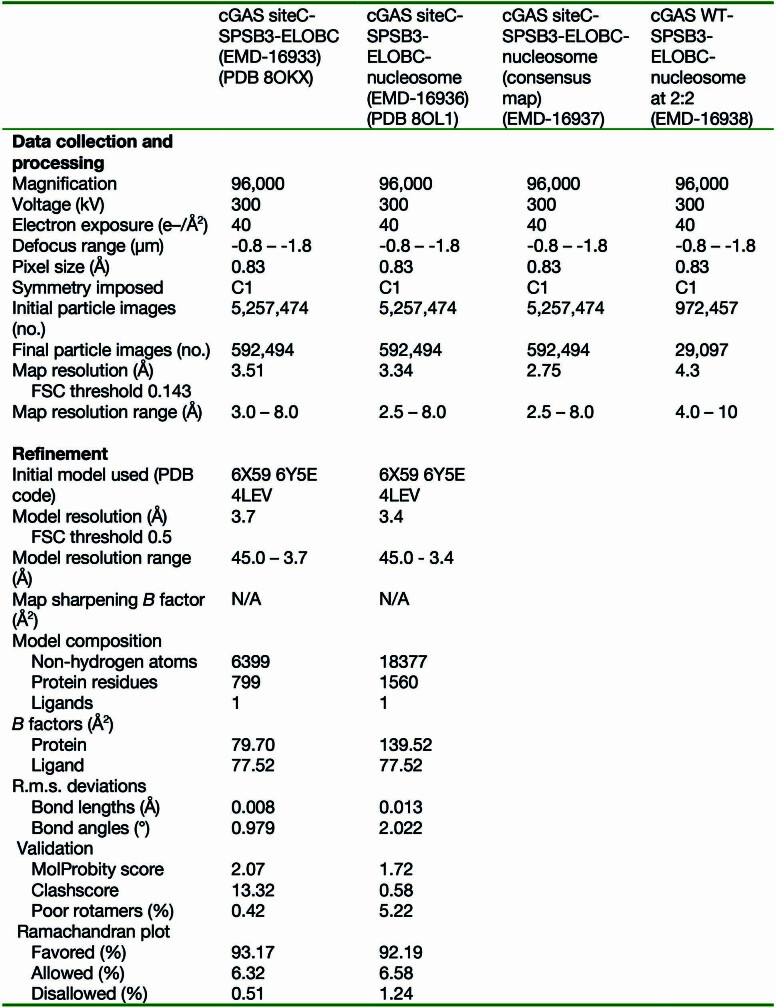
Cryo-EM data collection, refinement and statistics

## Data Availability

The 3D cryo-EM density maps have been deposited in the Electron Microscopy Data Bank under the accession numbers EMD-16933 (focused refinement with cGAS(site C)–SPSB3–ELOBC mask), EMD-16936 (composite map of cGAS(site C)–SPSB3–ELOBC–nucleosome complex), EMD-16937 (consensus refinement of cGAS(site C)–SPSB3–ELOBC–nucleosome complex), EMD-16938 (cGAS(WT)–SPSB3–ELOBC–nucleosome complex at a 2:2 ratio). The coordinates have been deposited at the PDB under accession numbers 8OKX (cGAS(site C)–SPSB3–ELOBC) and 8OL1 (cGAS(site C)–SPSB3–ELOBC–nucleosome). All data are presented in the Article and the Supplementary Information. Source data are provided with this paper.
