# Supplementary material for: The CRL5–SPSB3 ubiquitin ligase targets nuclear cGAS for degradation
Source: Nature. 2024 Feb 28;627(8005):873–9. doi: 10.1038/s41586-024-07112-w (PMC10972748; doi:10.1038/s41586-024-07112-w)
Supplement: Supplementary file 1 — Supplementary Figs. 1 and 2. [file 41586_2024_7112_MOESM1_ESM.pdf]

nature portfolio

https://doi.org/10.1038/s41586-024-07112-w

Supplementary information

The CRL5–SPSB3 ubiquitin ligase targets nuclear cGAS for degradation

In the format provided by the authors and unedited

The CRL5^{SPSB3} ubiquitin ligase targets nuclear cGAS for degradation

Pengbiao Xu[1,3], Ying Liu[1,3], Chong Liu[1,3], Baptiste Guey[1], Lingyun Li[1], Pauline Melenec[1], Jonathan Ricci[1] & Andrea Ablasser[1,2,*]

[1] Global Health Institute, Swiss Federal Institute of Technology Lausanne (EPFL), Lausanne, Switzerland

[2] Institute for Cancer Research (ISREC), Swiss Federal Institute of Technology Lausanne (EPFL), Lausanne, Switzerland

[3] These authors contributed equally: Pengbiao Xu, Ying Liu, Chong Liu

* Correspondence: andrea.ablasser@epfl.ch

Supplementary Figure 1 | Full scans for all immunoblots and Coomassie Blue stainings.

Supplementary Figure 2 | Flow cytometric gating strategy

Fig.1

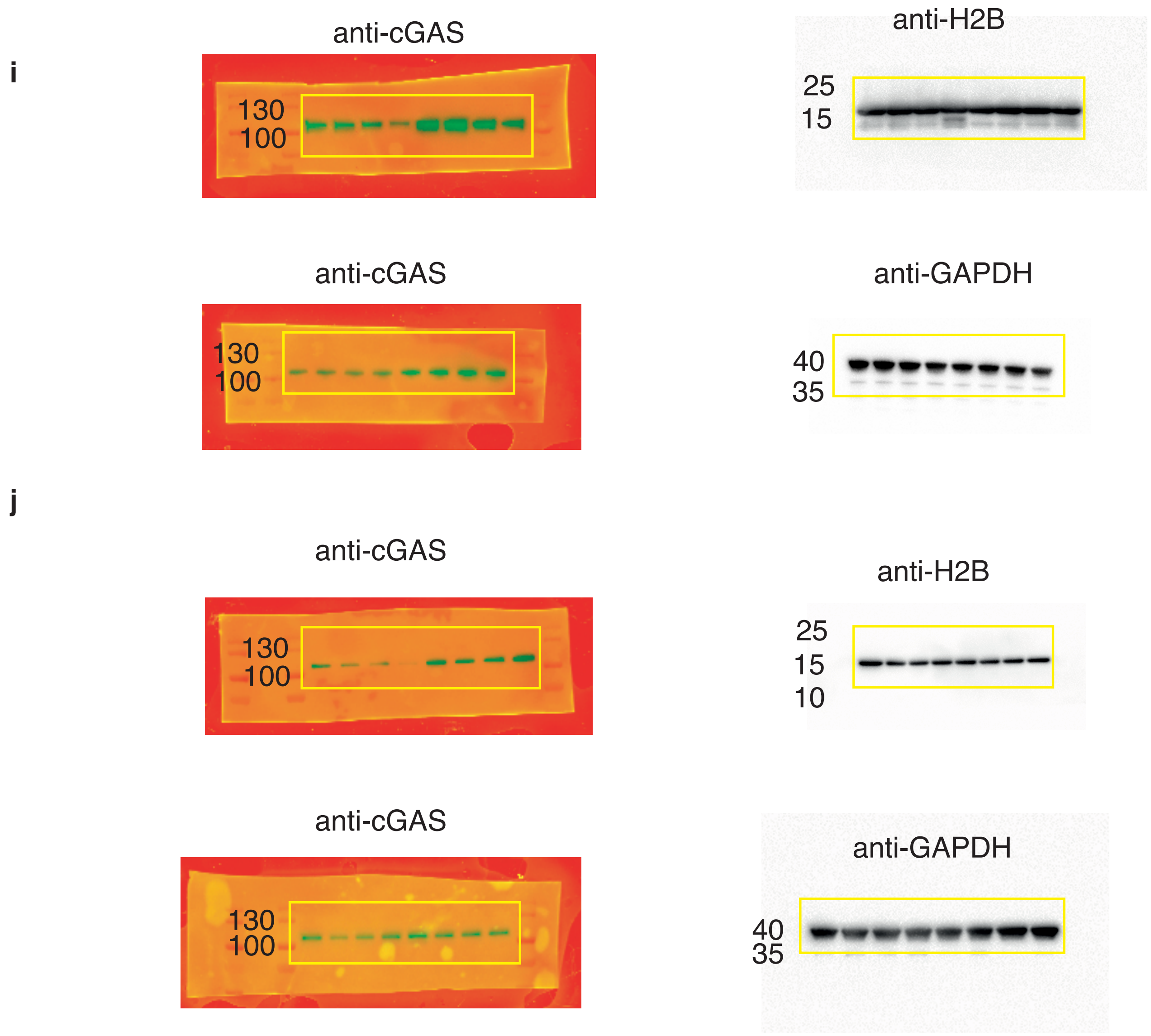

i
anti-cGAS
130
100
anti-H2B
25
15
anti-cGAS
130
100
anti-GAPDH
40
35
j
anti-cGAS
130
100
anti-H2B
25
15
10
anti-cGAS
130
100
anti-GAPDH
40
35

Fig.2

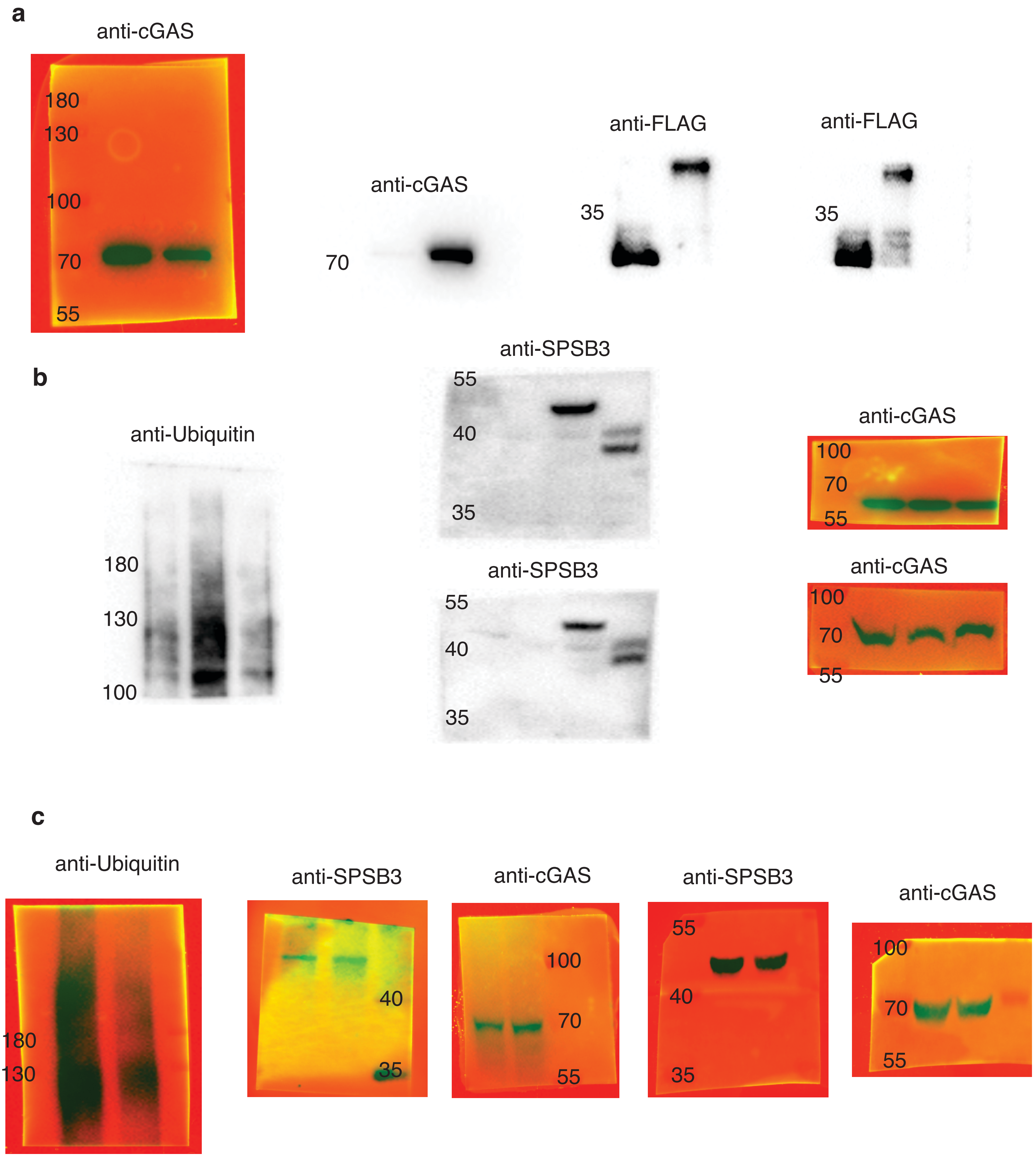
a
anti-cGAS
180
130
100
70
55
anti-cGAS
70
anti-FLAG
35
anti-FLAG
35
b
anti-Ubiquitin
180
130
100
anti-SPSB3
55
40
35
anti-SPSB3
55
40
35
anti-cGAS
100
70
55
anti-cGAS
100
70
55
c
anti-Ubiquitin
180
130
anti-SPSB3
40
35
anti-cGAS
100
70
55
anti-SPSB3
55
40
35
anti-cGAS
100
70
55

Fig.2

d

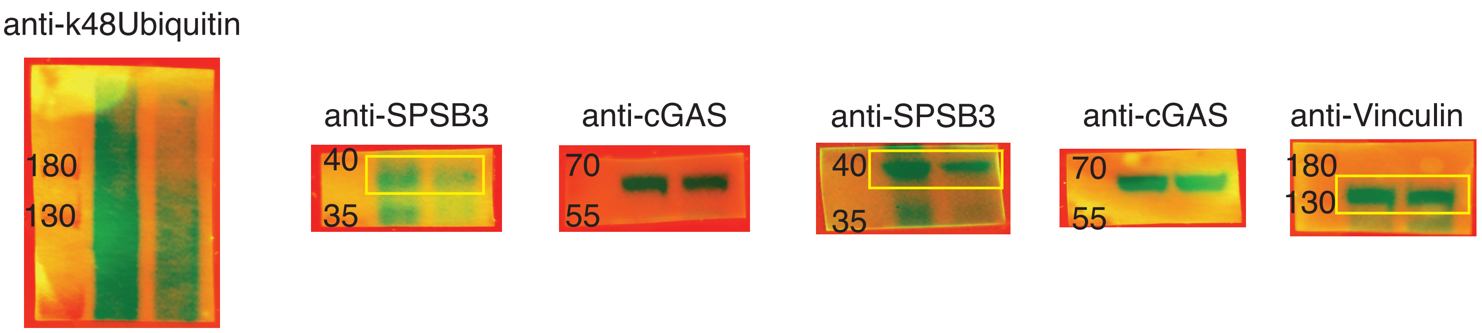
anti-k48Ubiquitin
180
130
anti-SPSB3
40
35
anti-cGAS
70
55
anti-SPSB3
40
35
anti-cGAS
70
55
anti-Vinculin
180
130

e

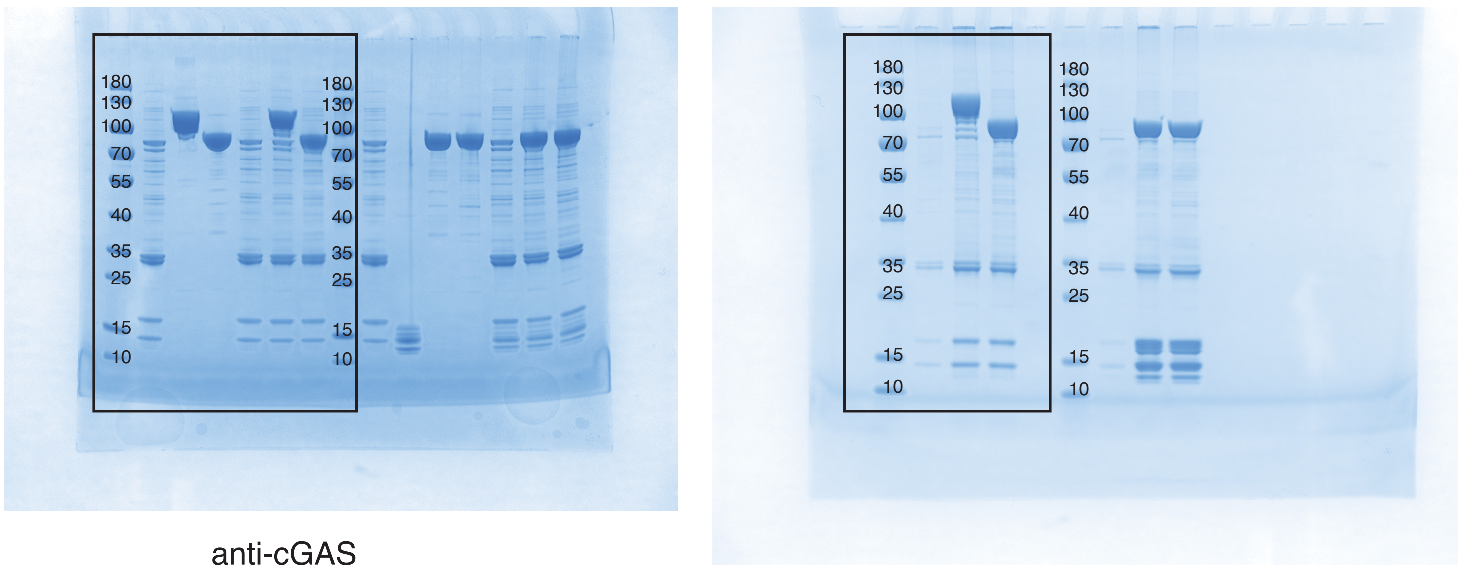
180
130
100
70
55
40
35
25
15
10

f

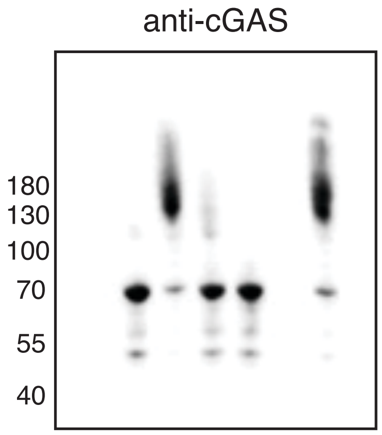
anti-cGAS
180
130
100
70
55
40

h

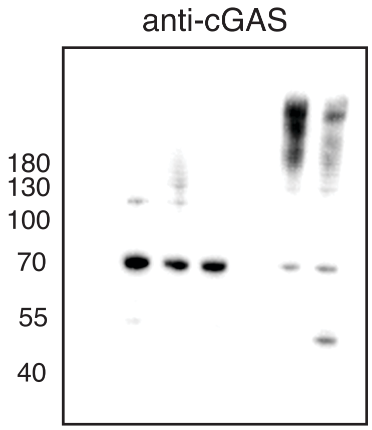
anti-cGAS
180
130
100
70
55
40

i

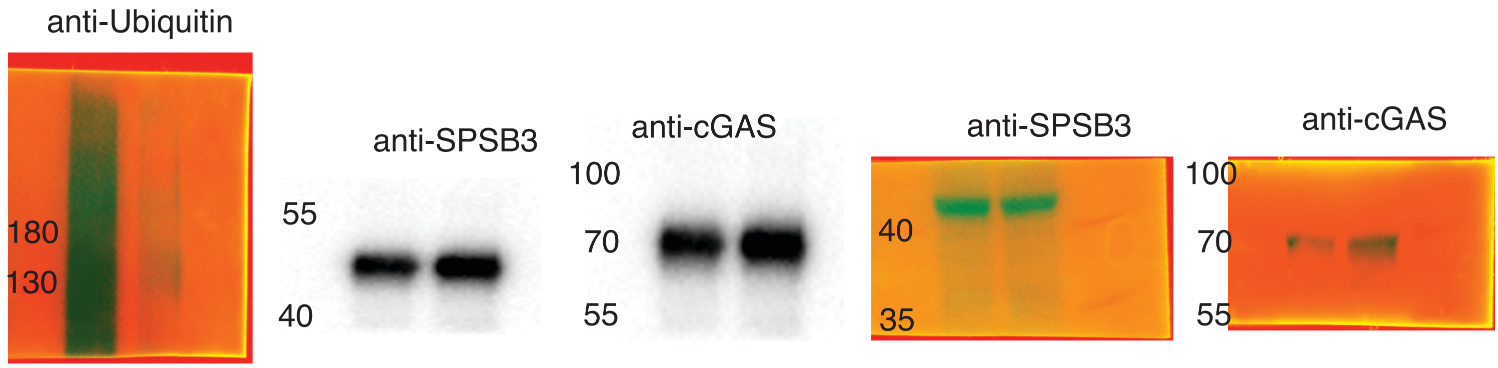
anti-Ubiquitin
180
130
anti-SPSB3
55
40
anti-cGAS
100
70
55
anti-SPSB3
40
35
anti-cGAS
100
70
55

Fig.4

b

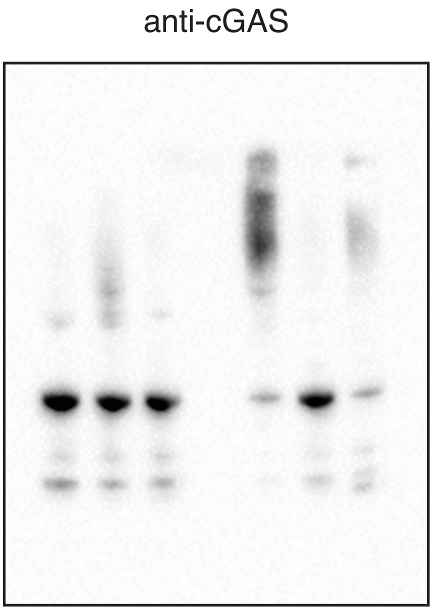
anti-cGAS

c

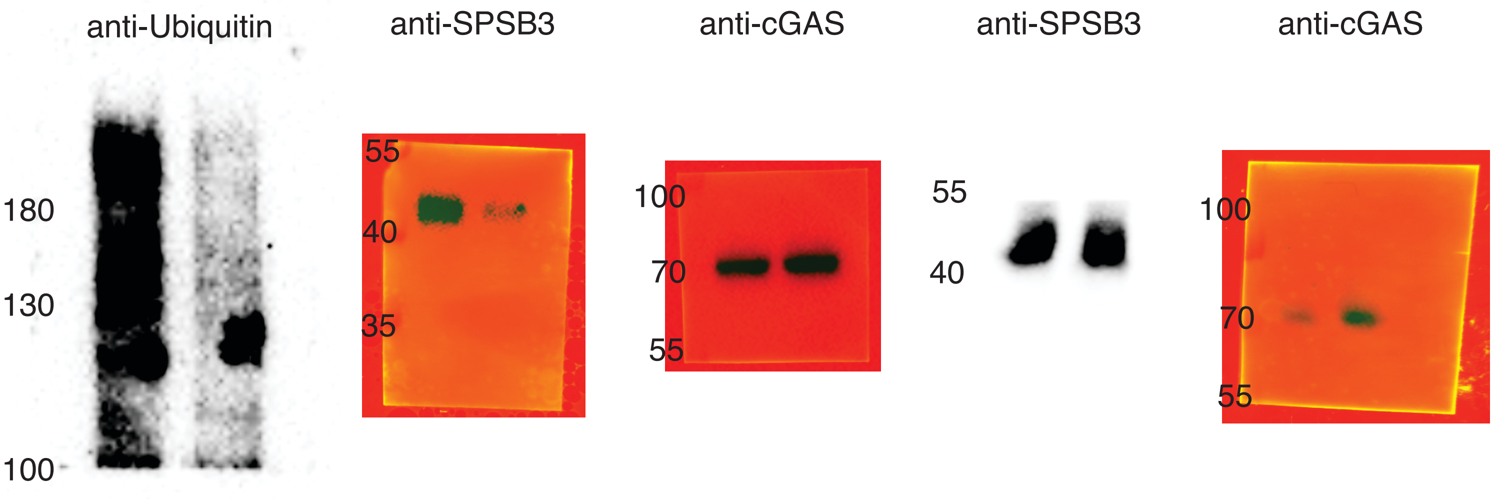
anti-Ubiquitin
180
130
100
anti-SPSB3
55
40
35
anti-cGAS
100
70
55
anti-SPSB3
55
40
anti-cGAS
100
70
55

d

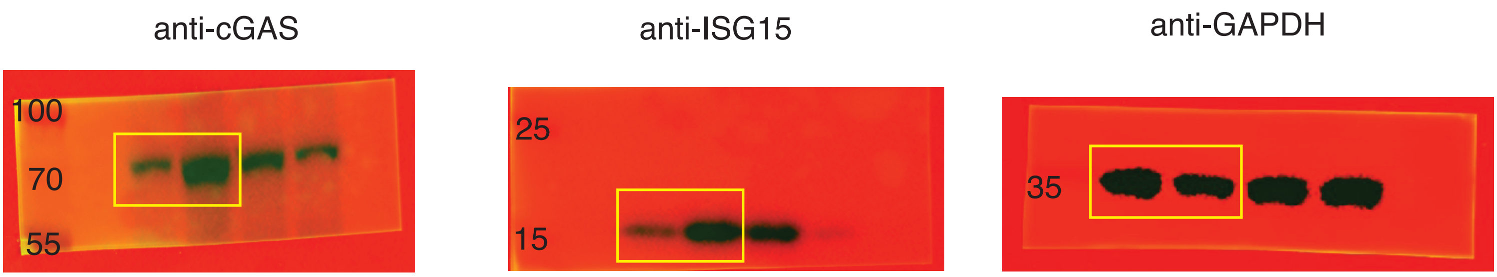
anti-cGAS
100
70
55
anti-ISG15
25
15
anti-GAPDH
35

Extended Fig.2

f

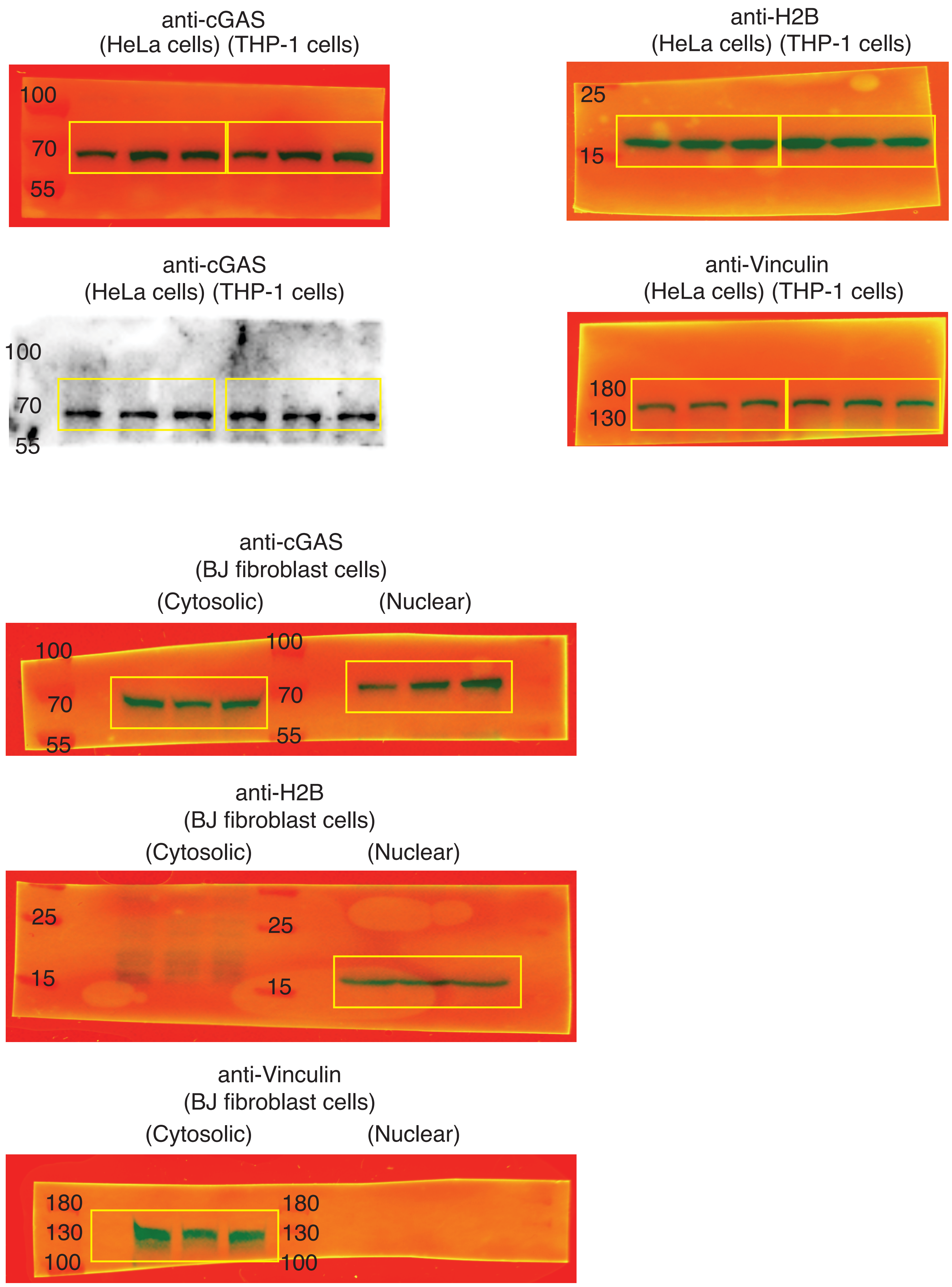

Extended Fig.3

e

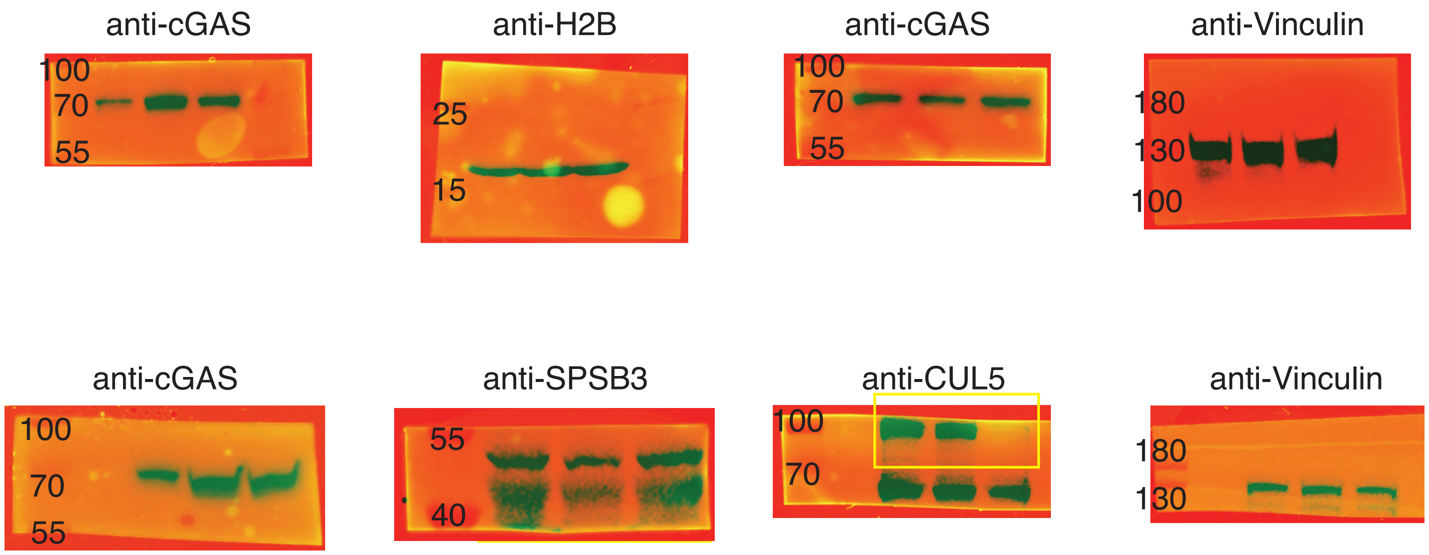

k

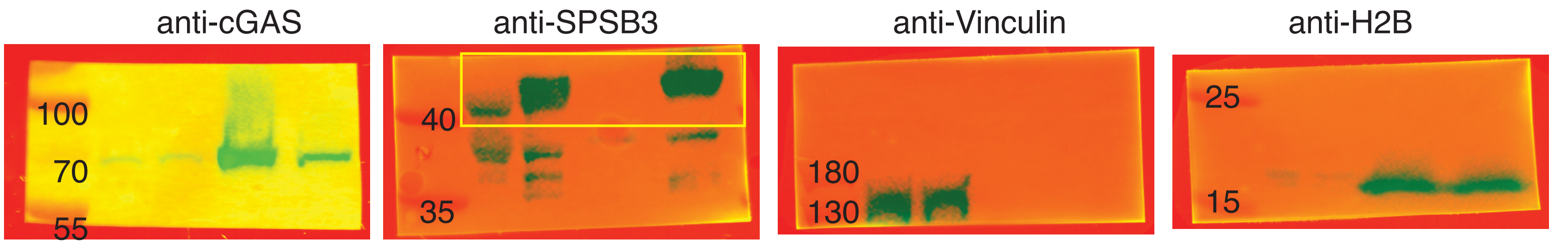

Extended Fig.3

i

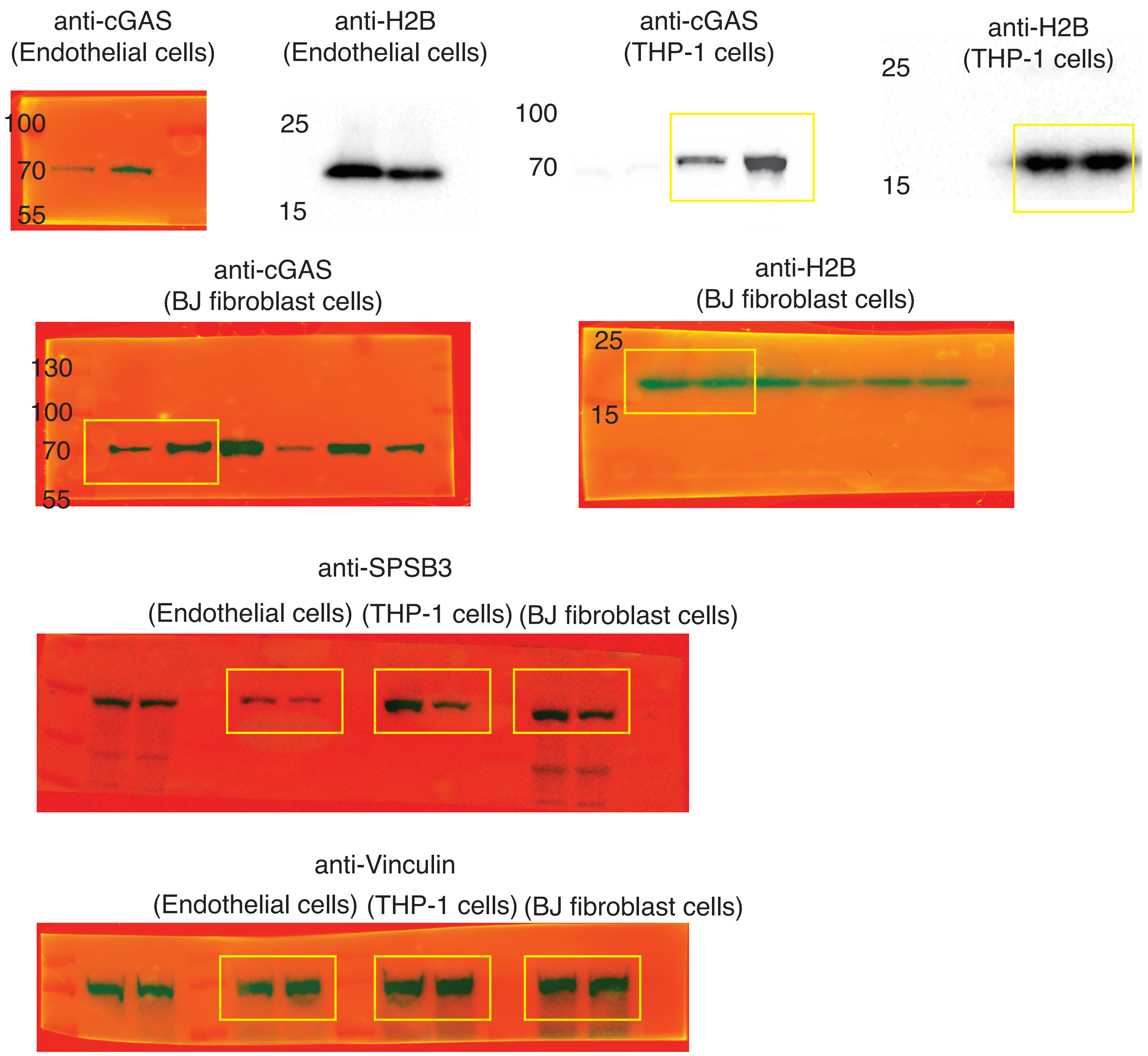

Extended Fig.4

a

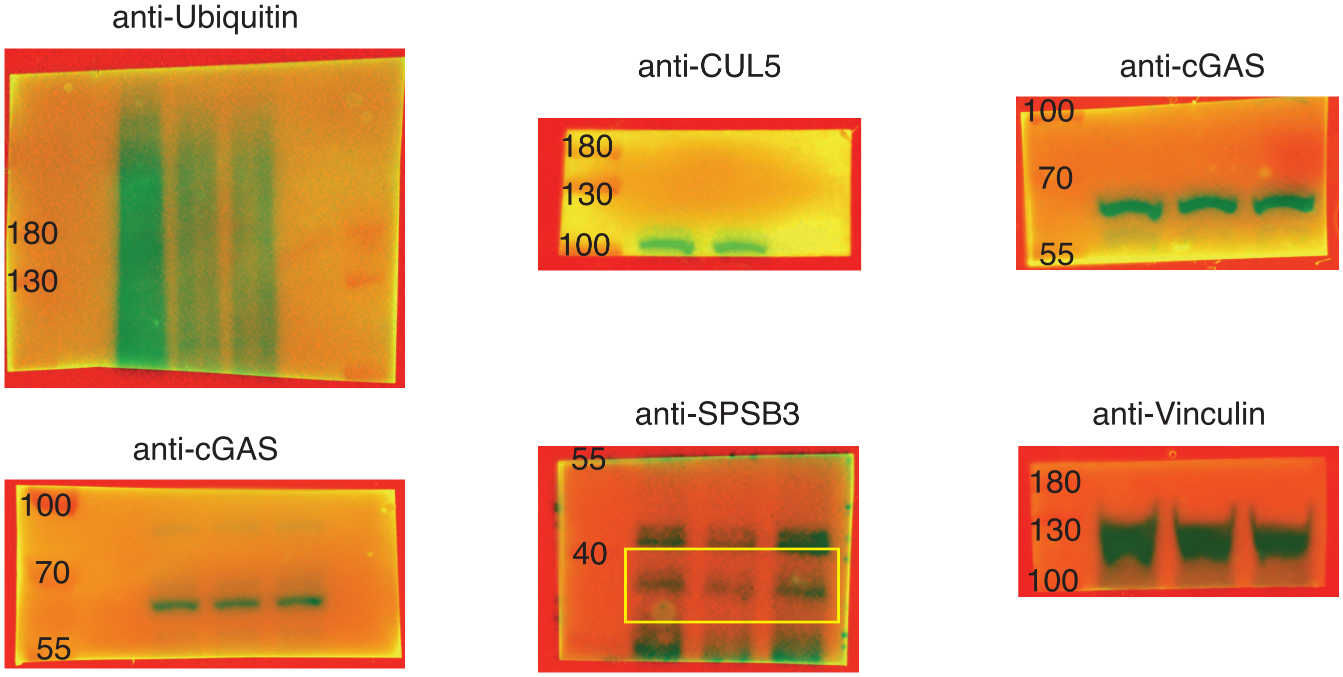

e

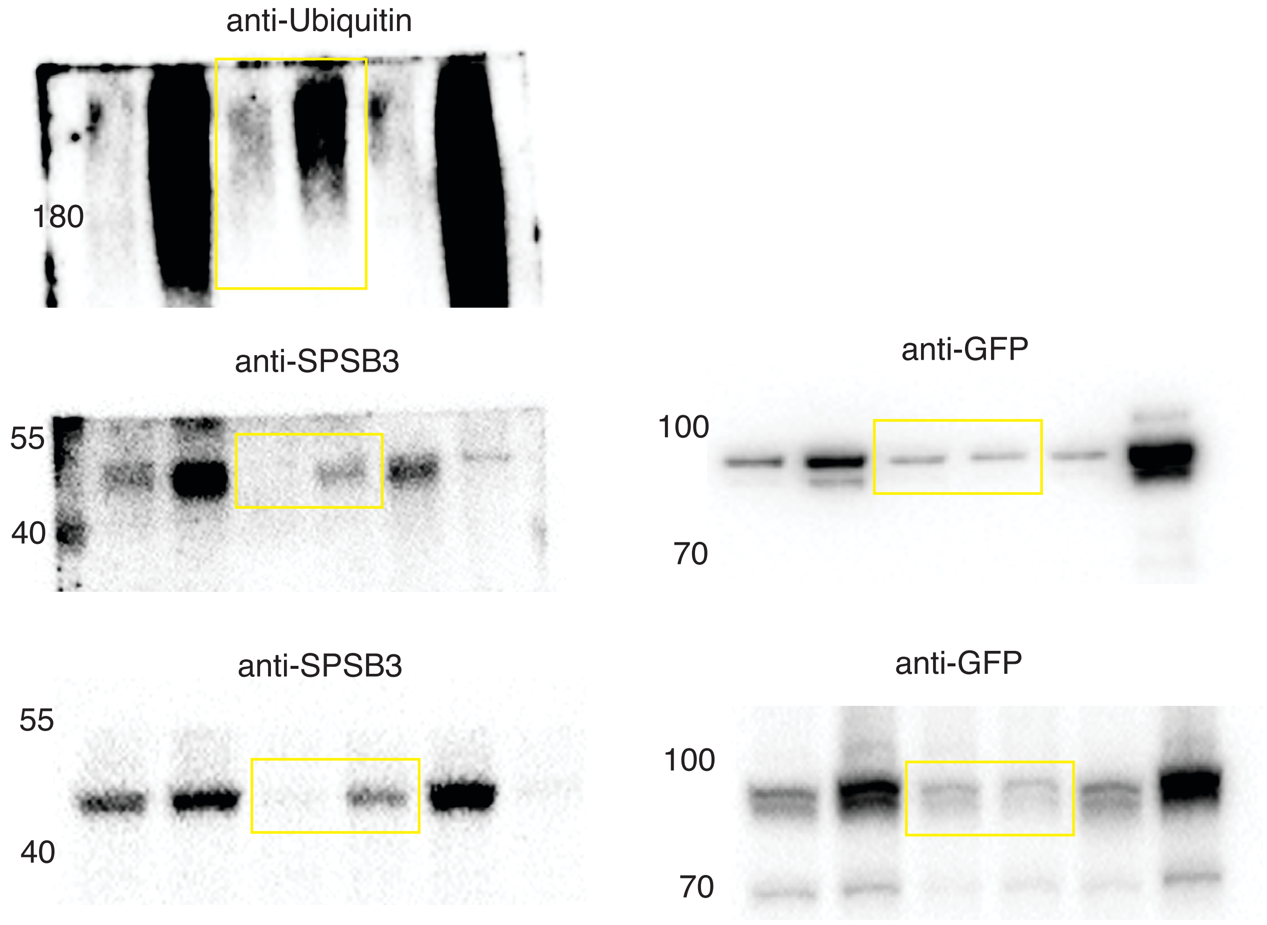

f

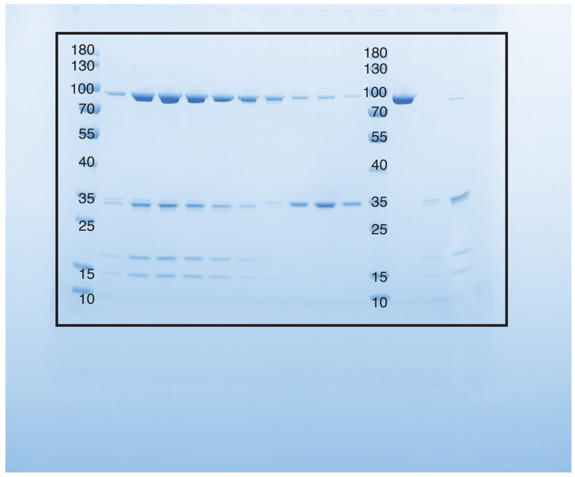

g

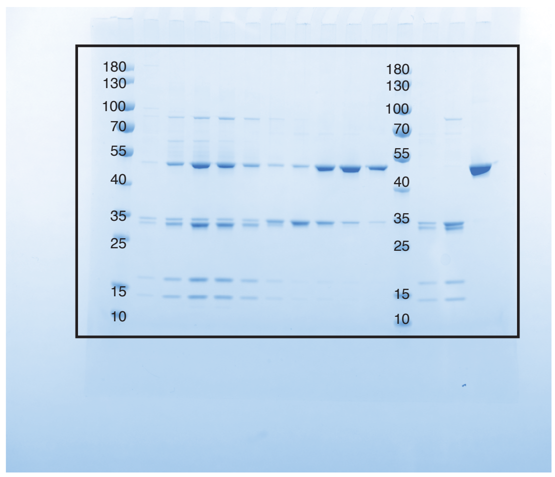

Extended Fig.5

a

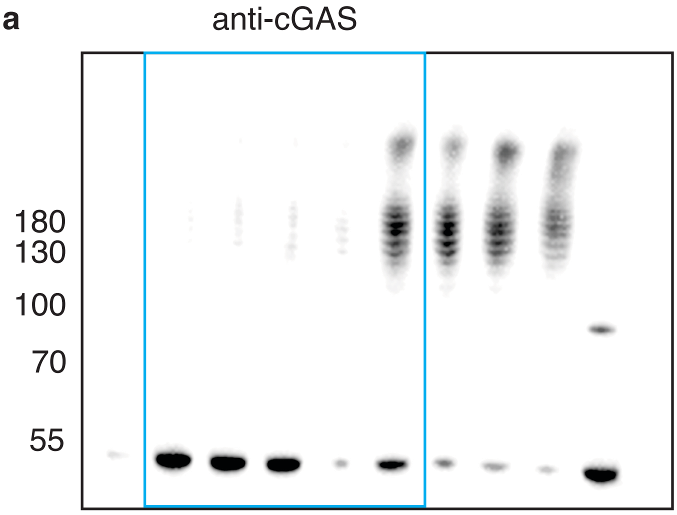

c

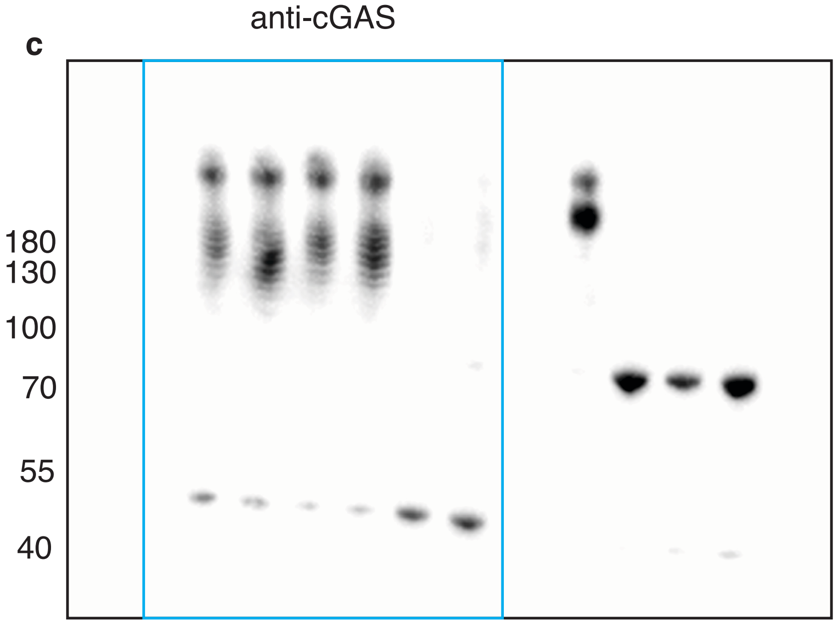

f

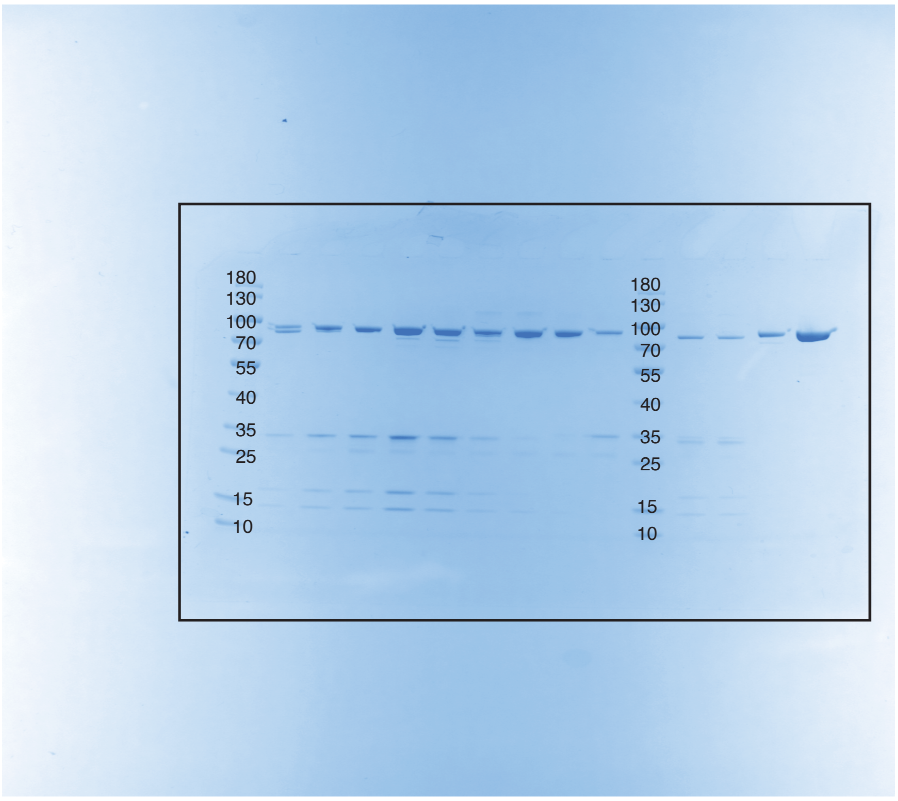

g

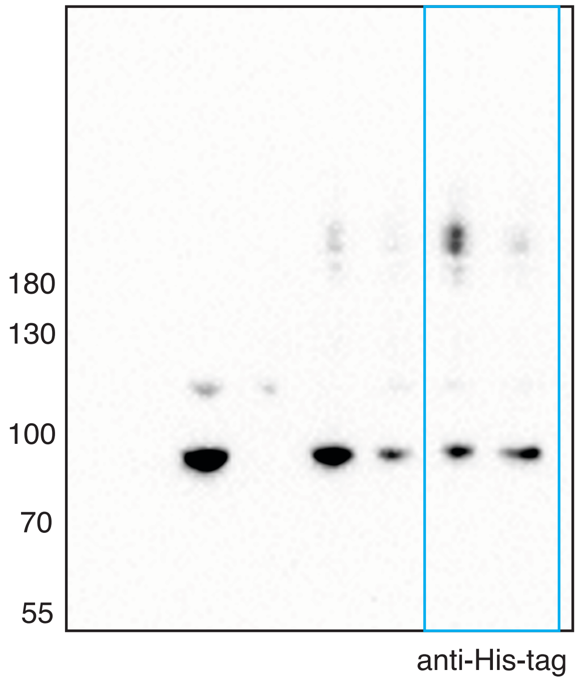

Extended Fig.6

a

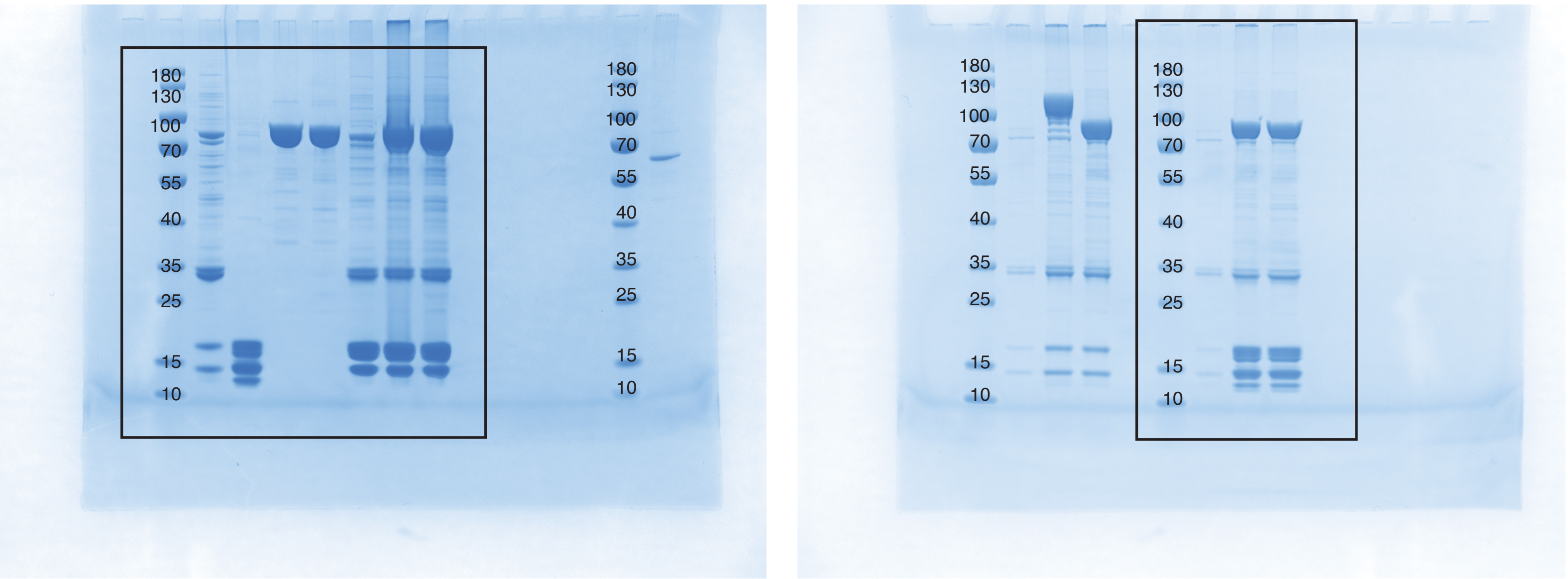

c

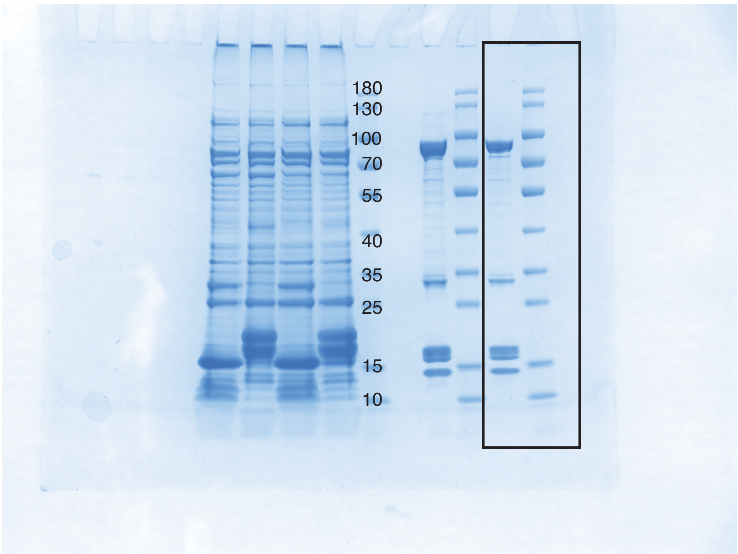

Extended Fig.7

a

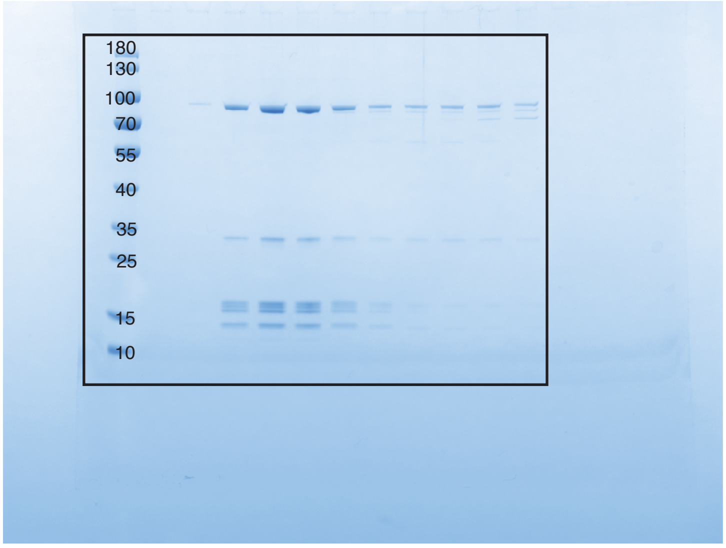

Extended Fig.9

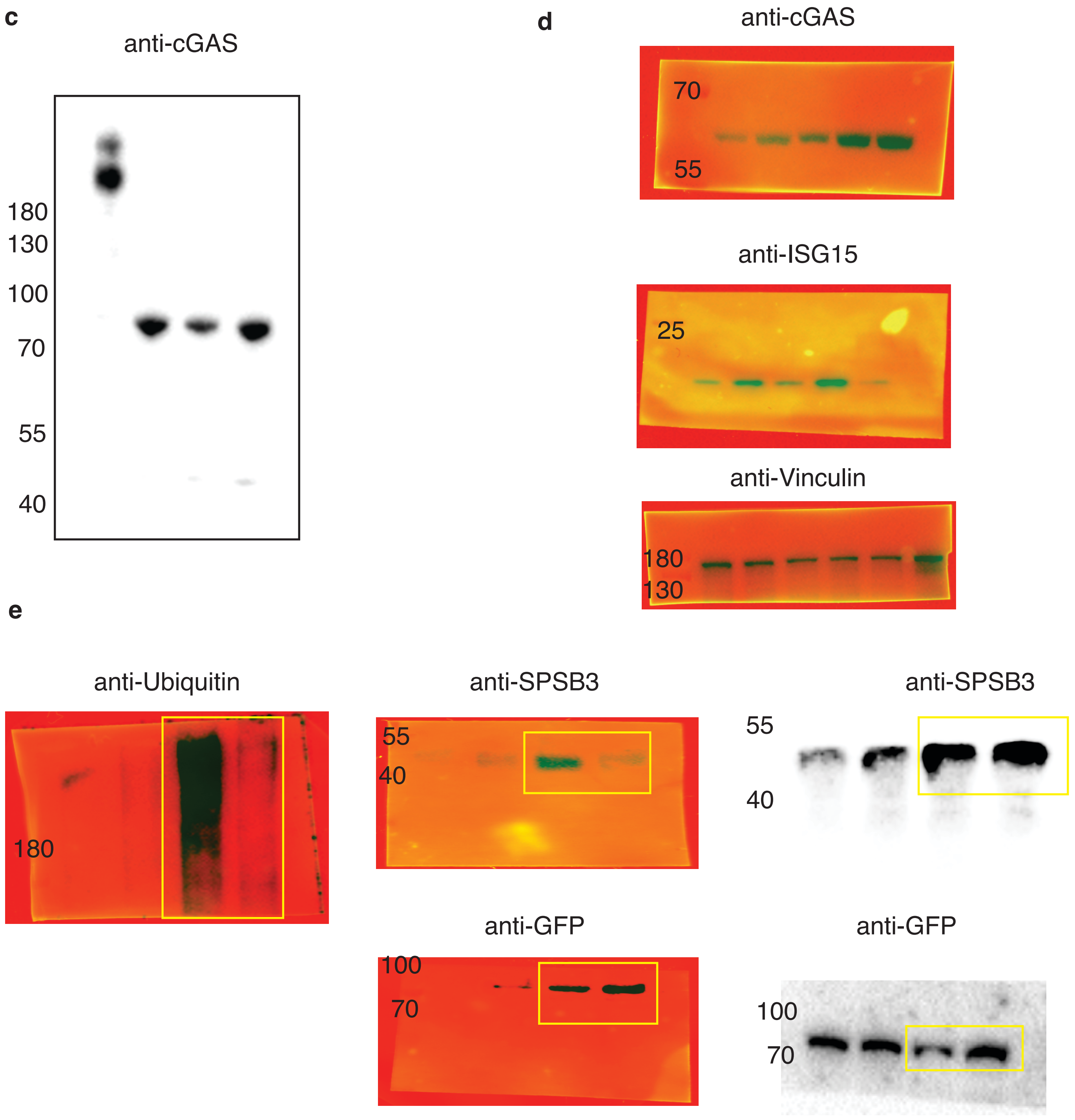

Extended Fig.10

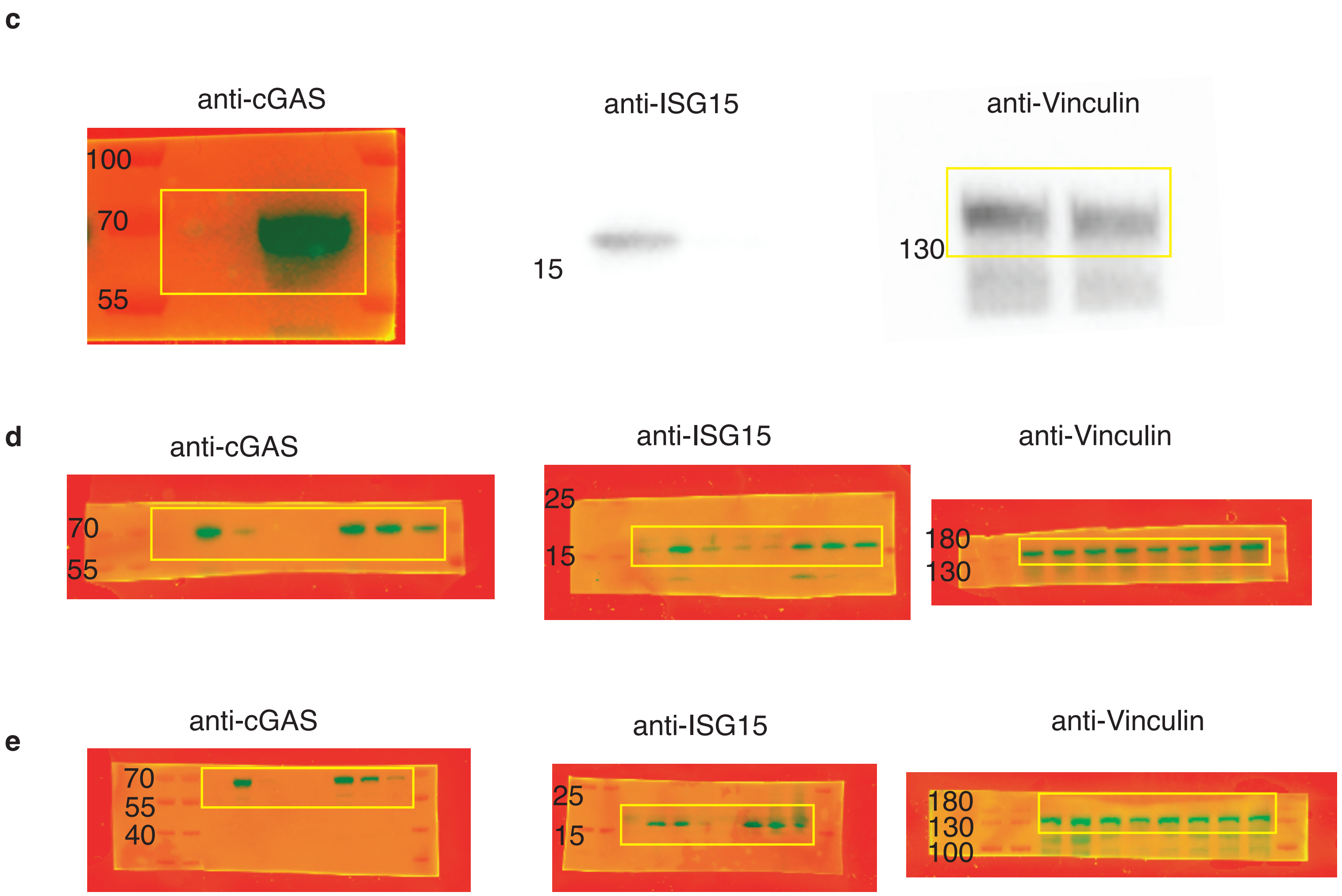

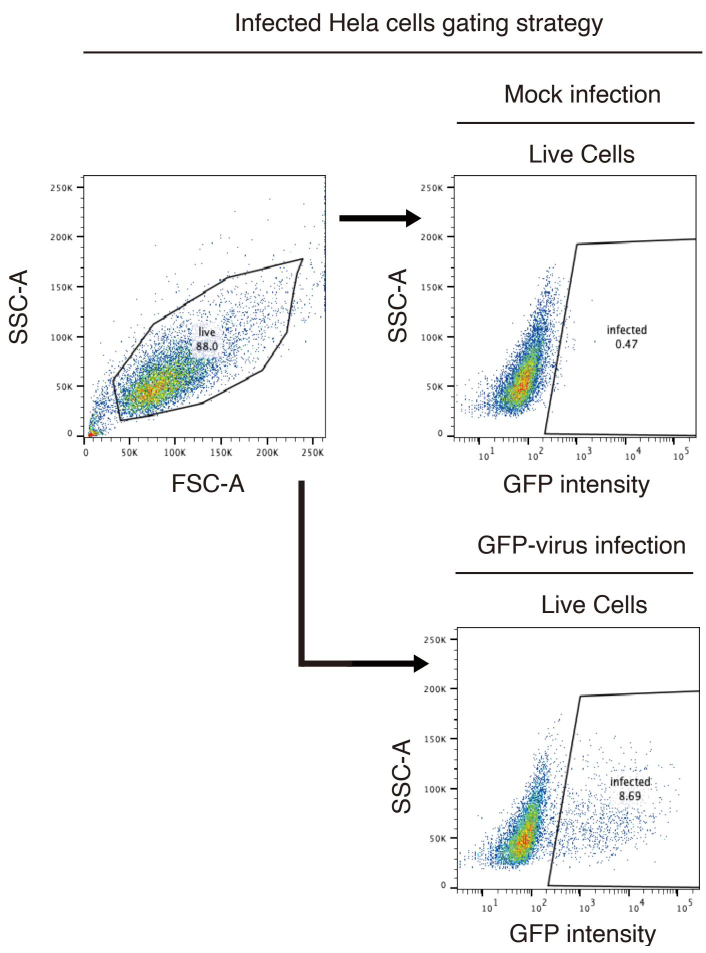

Supplementary Fig.2 | The flow cytometry gating strategy for quantification of GFP+ cells, related to Figure 4i, 4j and Extended Data Figure 11k, is shown.
