# Supplementary material for: The CRL5–SPSB3 ubiquitin ligase targets nuclear cGAS for degradation
Source: Nature. 2024 Feb 28;627(8005):873–9. doi: 10.1038/s41586-024-07112-w (PMC10972748; doi:10.1038/s41586-024-07112-w)
Supplement: Supplementary file 2 — Reporting Summary [file 41586_2024_7112_MOESM2_ESM.pdf]

nature portfolio

Corresponding author(s): Andrea Ablasser

Last updated by author(s): Nov 30, 2023

Reporting Summary

Nature Portfolio wishes to improve the reproducibility of the work that we publish. This form provides structure for consistency and transparency in reporting. For further information on Nature Portfolio policies, see our Editorial Policies and the Editorial Policy Checklist.

Statistics

For all statistical analyses, confirm that the following items are present in the figure legend, table legend, main text, or Methods section.

n/a	Confirmed	
☐	☒	The exact sample size (n) for each experimental group/condition, given as a discrete number and unit of measurement
☐	☒	A statement on whether measurements were taken from distinct samples or whether the same sample was measured repeatedly
☐	☒	The statistical test(s) used AND whether they are one- or two-sided<br>*Only common tests should be described solely by name; describe more complex techniques in the Methods section.*
☒	☐	A description of all covariates tested
☐	☒	A description of any assumptions or corrections, such as tests of normality and adjustment for multiple comparisons
☐	☒	A full description of the statistical parameters including central tendency (e.g. means) or other basic estimates (e.g. regression coefficient) AND variation (e.g. standard deviation) or associated estimates of uncertainty (e.g. confidence intervals)
☐	☒	For null hypothesis testing, the test statistic (e.g. F, t, r) with confidence intervals, effect sizes, degrees of freedom and P value noted<br>*Give P values as exact values whenever suitable.*
☒	☐	For Bayesian analysis, information on the choice of priors and Markov chain Monte Carlo settings
☒	☐	For hierarchical and complex designs, identification of the appropriate level for tests and full reporting of outcomes
☒	☐	Estimates of effect sizes (e.g. Cohen's d, Pearson's r), indicating how they were calculated

Our web collection on statistics for biologists contains articles on many of the points above.

Software and code

Policy information about availability of computer code

Data collection	Western blot and coomassie staining data were collected using CHemiDox XRS Biorad Imger and Image Lab Software (v6.1) or by JESS automated Western blotting system (ProteinSimple, San Jose, CA, USA).<br>RT-qPCR data were acquired using QuantStuido 7 Real-Time PCR system (Thermo Fisher).<br>Microscopy data were collected using LAS-X software (for the Leica SP8 confocal microscope), Zeiss ZEN software (for the Zeiss LSM980 microscope) and Harmony (PerkinElmer) software (for the Operetta CLS machine).<br>Cryo-EM data was collected using EPU2.<br>LC-MS was performed on Dionex Ultimate 3000 RSLC nano UPLC system (Thermo Fischer Scientific).<br>Flow cytometry data were collected from LSRFortessa(BD) flow cytometry.
Data analysis	Western blot and coomassie staining data were analysed using ImageLab (v6.1) using Compass for SW 6.1 software for JESS samples.<br>Statistical analysis were analysed with GraphPad PRISM 9 (v9.3.1).<br>Microscopy data on the confocal images were analyzed with Fiji (v2.3.0), using basic embedded tools.<br>Microscopy data on the brightfield images from the Operetta microscopy were analyzed with PerkinElmer Harmony (v4.9).<br>Data tables obtained from PerkinElmer Harmony (v4.9) were then processed with CellProfiler (v4.2.6) and KNIME (v4.7.4) for selecting results of interest and allowing batch calculations. Final results were then analysed and plotted using GraphPad PRISM 9 (v9.3.1).<br>Cryo-EM structure was reconstructed by Cryosparc (v.4.2), Phenix (v.1.20rc2), Coot (v. 0.8.9.3), UCSF Chimera (v.1.15), UCSF ChimeraX (v.1.5)<br>Data from LC-MS was processed using SEQUEST, MS Amanda and MS Fragger in Proteome Discoverer (2.5). Scaffold (5.1.0) was used for further inspection.<br>Flow Cytometry data were analyzed with FlowJo Version 10.8.2.

For manuscripts utilizing custom algorithms or software that are central to the research but not yet described in published literature, software must be made available to editors and reviewers. We strongly encourage code deposition in a community repository (e.g. GitHub). See the Nature Portfolio guidelines for submitting code & software for further information.

Data

Policy information about availability of data

All manuscripts must include a data availability statement. This statement should provide the following information, where applicable:

- Accession codes, unique identifiers, or web links for publicly available datasets
- A description of any restrictions on data availability
- For clinical datasets or third party data, please ensure that the statement adheres to our policy

The 3D cryo-EM density maps are deposited in the Electron Microscopy Data Bank under the accession numbers EMD-16933 (Focused refinement with cGAS site C-SPSB3-ELOBC mask), EMD-16936 (composite map of cGAS site C-SPSB3-ELOBC-nucleosome complex), EMD-16937 (Consensus refinement of cGAS site C-SPSB3-ELOBC-nucleosome complex), EMD-16938 (cGAS WT-SPSB3-ELOBC-nucleosome complex at 2:2 ratio). The coordinates are deposited in the PDB with accession numbers 8OKX (cGAS site C-SPSB3-ELOBC) and 8OL1 (cGAS site C-SPSB3-ELOBC-nucleosome). All data are presented in this manuscript and its supplementary information. Source data are provided with this paper.

Field-specific reporting

Please select the one below that is the best fit for your research. If you are not sure, read the appropriate sections before making your selection.

☒ Life sciences ☐ Behavioural & social sciences ☐ Ecological, evolutionary & environmental sciences

For a reference copy of the document with all sections, see nature.com/documents/nr-reporting-summary-flat.pdf

Life sciences study design

All studies must disclose on these points even when the disclosure is negative.

Sample size	No statistical method was used to predetermine sample size as this is not relevant for biochemical and structural experiments. For cellular assays, sample sizes were chosen based on prior experience and on common practices in the field.
Data exclusions	No data were excluded.
Replication	All experiments were independently repeated with replicates as stated in the figure legends. Panels displaying one representative experiment were validated by independent experiments showing similar results.
Randomization	There was no randomization as this is not relevant to the experiments of this study.
Blinding	Data analysis was not blinded, because no subjective analysis was performed.

Behavioural & social sciences study design

All studies must disclose on these points even when the disclosure is negative.

Study description	*Briefly describe the study type including whether data are quantitative, qualitative, or mixed-methods (e.g. qualitative cross-sectional, quantitative experimental, mixed-methods case study).*
Research sample	*State the research sample (e.g. Harvard university undergraduates, villagers in rural India) and provide relevant demographic information (e.g. age, sex) and indicate whether the sample is representative. Provide a rationale for the study sample chosen. For studies involving existing datasets, please describe the dataset and source.*
Sampling strategy	*Describe the sampling procedure (e.g. random, snowball, stratified, convenience). Describe the statistical methods that were used to predetermine sample size OR if no sample-size calculation was performed, describe how sample sizes were chosen and provide a rationale for why these sample sizes are sufficient. For qualitative data, please indicate whether data saturation was considered, and what criteria were used to decide that no further sampling was needed.*
Data collection	*Provide details about the data collection procedure, including the instruments or devices used to record the data (e.g. pen and paper, computer, eye tracker, video or audio equipment) whether anyone was present besides the participant(s) and the researcher, and whether the researcher was blind to experimental condition and/or the study hypothesis during data collection.*
Timing	*Indicate the start and stop dates of data collection. If there is a gap between collection periods, state the dates for each sample cohort.*
Data exclusions	*If no data were excluded from the analyses, state so OR if data were excluded, provide the exact number of exclusions and the rationale behind them, indicating whether exclusion criteria were pre-established.*

Non-participation	*State how many participants dropped out/declined participation and the reason(s) given OR provide response rate OR state that no participants dropped out/declined participation.*
Randomization	*If participants were not allocated into experimental groups, state so OR describe how participants were allocated to groups, and if allocation was not random, describe how covariates were controlled.*

Ecological, evolutionary & environmental sciences study design

All studies must disclose on these points even when the disclosure is negative.

Study description	*Briefly describe the study. For quantitative data include treatment factors and interactions, design structure (e.g. factorial, nested, hierarchical), nature and number of experimental units and replicates.*
Research sample	*Describe the research sample (e.g. a group of tagged Passer domesticus, all Stenocereus thurberi within Organ Pipe Cactus National Monument), and provide a rationale for the sample choice. When relevant, describe the organism taxa, source, sex, age range and any manipulations. State what population the sample is meant to represent when applicable. For studies involving existing datasets, describe the data and its source.*
Sampling strategy	*Note the sampling procedure. Describe the statistical methods that were used to predetermine sample size OR if no sample-size calculation was performed, describe how sample sizes were chosen and provide a rationale for why these sample sizes are sufficient.*
Data collection	*Describe the data collection procedure, including who recorded the data and how.*
Timing and spatial scale	*Indicate the start and stop dates of data collection, noting the frequency and periodicity of sampling and providing a rationale for these choices. If there is a gap between collection periods, state the dates for each sample cohort. Specify the spatial scale from which the data are taken*
Data exclusions	*If no data were excluded from the analyses, state so OR if data were excluded, describe the exclusions and the rationale behind them, indicating whether exclusion criteria were pre-established.*
Reproducibility	*Describe the measures taken to verify the reproducibility of experimental findings. For each experiment, note whether any attempts to repeat the experiment failed OR state that all attempts to repeat the experiment were successful.*
Randomization	*Describe how samples/organisms/participants were allocated into groups. If allocation was not random, describe how covariates were controlled. If this is not relevant to your study, explain why.*
Blinding	*Describe the extent of blinding used during data acquisition and analysis. If blinding was not possible, describe why OR explain why blinding was not relevant to your study.*

Did the study involve field work? ☐ Yes ☐ No

Field work, collection and transport

Field conditions	*Describe the study conditions for field work, providing relevant parameters (e.g. temperature, rainfall).*
Location	*State the location of the sampling or experiment, providing relevant parameters (e.g. latitude and longitude, elevation, water depth).*
Access & import/export	*Describe the efforts you have made to access habitats and to collect and import/export your samples in a responsible manner and in compliance with local, national and international laws, noting any permits that were obtained (give the name of the issuing authority, the date of issue, and any identifying information).*
Disturbance	*Describe any disturbance caused by the study and how it was minimized.*

Reporting for specific materials, systems and methods

We require information from authors about some types of materials, experimental systems and methods used in many studies. Here, indicate whether each material, system or method listed is relevant to your study. If you are not sure if a list item applies to your research, read the appropriate section before selecting a response.

Materials & experimental systems

n/a	Involved in the study
☐	☒ Antibodies
☐	☒ Eukaryotic cell lines
☒	☐ Palaeontology and archaeology
☒	☐ Animals and other organisms
☒	☐ Human research participants
☒	☐ Clinical data
☒	☐ Dual use research of concern

Methods

n/a	Involved in the study
☒	☐ ChIP-seq
☐	☒ Flow cytometry
☒	☐ MRI-based neuroimaging

Antibodies

Antibodies used

Primary antibodies used: mouse monoclonal anti-Vinculin (hVIN-1) (Sigma-Aldrich, V9264), rabbit monoclonal anti-GAPDH (14C10) (Cell Signaling Technology, 2118), mouse monoclonal anti-FLAG (M2) (Sigma-Aldrich, F1804), rabbit monoclonal anti-ISG15 (EPR24482-49) (abcam, ab285367), rabbit polyconal anti-ISG15 (Cell Signaling Technology, 2743), rabbit polyclonal anti-cGAS (Novus, NBP3-16666), rabbit monoclonal anti-cGAS (D3O8O) (Cell Signaling Technology, 31659), rabbit monoclonal anti-cGAS (D1D3G) (Cell Signaling Technology, 15102), rabbit monoclonal anti-cGAS (E5V3W) (Cell Signaling Technology, 79978), mouse monoclonal anti-Ubiquitin (P4D1) (Santa Cruz, sc-8017), rabbit monoclonal anti-Ubiquitin (linage-specific K48) (EP8589) (Abcam ab140601), rabbit polyclonal anti-SPSB3 (Novus Biologicals, NBP2-20480), rabbit polyclonal anti-SPSB3(Aviva, ARP71676_P050), rabbit polyclonal anti-H3pS10 (Sigma-Aldrich, 06-570), rabbit polyclonal anti-CUL5 (Abcam, ab264284), mouse monoclonal anti-His-Tag (27E8) (Cell Signaling Technology, 2366),rabbit monoclonal anti-H2B (mAbcam-52484) (Abcam, 52484). Secondary antibodies used: HRP-conjugated secondary antibodies used: Donkey anti-rabbit IgG (H+L)-HRP (Jackson ImmunoResearch, 711-036-152), Donkey anti-mouse IgG (H+L)-HRP (Jackson ImmunoResearch, 715-036-151). Fluorescence-conjugated secondary antibodies used: Goat anti-Rabbit IgG (H+L) Cross-Adsorbed Secondary Antibody, Alexa Fluor 568-conjugated (Invitrogen, A-11011), Goat anti-Rabbit IgG (H+L) Cross-Adsorbed Secondary Antibody, Alexa Fluor 488-conjugated (Invitrogen, A-11008), Click-iT EdU Imaging Kit with Alexa Fluor 647 (Thermo Fisher, C10086).

Validation

We did not validate the antibodies used in this study, but we ascertained by using appropriate controls that conclusions of experiments using these antibodies are correct. The commercial source of each antibody is listed below and the corresponding validation can be found on the website of each supplier.

Primary antibodies used: mouse monoclonal anti-Vinculin (hVIN-1) (Sigma-Aldrich, V9264, immunoblot 1:5000), rabbit monoclonal anti-GAPDH (14C10) (Cell Signaling Technology, 2118, immunoblot 1:3000), mouse monoclonal anti-FLAG (M2) (Sigma-Aldrich, F1804, immunoblot 1:5000), rabbit monoclonal anti-ISG15 (EPR24482-49) (abcam, ab285367, immunoblot 1:1000), rabbit polyconal anti-ISG15 (Cell Signaling Technology, 2743, immunoblot 1:1000), rabbit polyclonal anti-cGAS (Novus, NBP3-16666, IP 1:50), rabbit monoclonal anti-cGAS (D3O8O) (Cell Signaling Technology, 31659 , immunoblot 1:1000), rabbit monoclonal anti-cGAS (D1D3G) (Cell Signaling Technology, 15102, immunoblot 1:1000), rabbit monoclonal anti-cGAS (E5V3W) (Cell Signaling Technology, 79978, immunoblot 1:1000; immunofluorescence 1:200), mouse monoclonal anti-Ubiquitin (P4D1) (Santa Cruz, sc-8017, 1:500), rabbit monoclonal anti-Ubiquitin (linage-specific K48) (EP8589) (Abcam ab140601, immunoblot 1:200), -rabbit polyclonal anti-SPSB3 (Novus Biologicals, NBP2-20480, immunoblot 1:1000), rabbit polyclonal anti-SPSB3(Aviva, ARP71676_P050, immunofluorescence 1:100, immunoblot 1:1000), rabbit polyclonal anti-H3pS10 (Sigma-Aldrich, 06-570, immunofluorescence 1:200), rabbit polyclonal anti-CUL5 (Abcam, ab264284, immunofluorescence 1:100), mouse monoclonal anti-His-Tag (27E8) (Cell Signaling Technology, 2366, immunoblot 1:1000), rabbit monoclonal anti-H2B (Abcam, 52484, immunoblot 1:5000). HRP-conjugated secondary antibodies used: Donkey anti-rabbit IgG (H+L)-HRP (Jackson ImmunoResearch, 711-036-152, immunoblot: 1:5000) and Donkey anti-mouse IgG (H+L)-HRP (Jackson ImmunoResearch, 715-036-151, immunoblot: 1:5000). JESS-specific anti-rabbit secondary HRP Antibody (Bio-Techne, 042-206, JESS 1:1), JESS-specific anti-mouse secondary NIR Antibody (Bio-Techne, 043-821, JESS 1:20). Fluorescence-conjugated secondary antibodies used: Goat anti-Rabbit IgG (H+L) Cross-Adsorbed Secondary Antibody, Alexa Fluor 568-conjugated (Invitrogen, A-11011, immunofluorescence 1:800), Goat anti-Rabbit IgG (H+L) Cross-Adsorbed Secondary Antibody, Alexa Fluor 488-conjugated (Invitrogen, A-11008, immunofluorescence 1:800), Click-iT EdU Imaging Kit with Alexa Fluor 647 (Thermo Fisher, C10086, following manufacturer's instruction).

Eukaryotic cell lines

Policy information about cell lines

Cell line source(s)

HeLa (CCL-2) cells were obtained from Sigma-Aldrich. HEK 293T cells were a gift from D. Trono (EPFL), originally purchased from ATCC. U2OS cells, THP-1 cells and BJ-5ta cells were obtained from ATCC. CT26 mouse colorectal carcinoma cells were a gift from D. Hanahan lab (EPFL). Primary human endothelial cells were obtained from a commercial supplier (Cell Biologics).

Authentication

Cells were not additionally authenticated.

Mycoplasma contamination

All cell lines were tested to be mycoplasma-negative by PCR repeatly.

Commonly misidentified lines
(See ICLAC register)

No commonly misidentified cell lines are used in this study.

Palaeontology and Archaeology

Specimen provenance	*Provide provenance information for specimens and describe permits that were obtained for the work (including the name of the issuing authority, the date of issue, and any identifying information). Permits should encompass collection and, where applicable, export.*
Specimen deposition	*Indicate where the specimens have been deposited to permit free access by other researchers.*
Dating methods	*If new dates are provided, describe how they were obtained (e.g. collection, storage, sample pretreatment and measurement), where they were obtained (i.e. lab name), the calibration program and the protocol for quality assurance OR state that no new dates are provided.*

☐ Tick this box to confirm that the raw and calibrated dates are available in the paper or in Supplementary Information.

Ethics oversight	*Identify the organization(s) that approved or provided guidance on the study protocol, OR state that no ethical approval or guidance was required and explain why not.*

Note that full information on the approval of the study protocol must also be provided in the manuscript.

Animals and other organisms

Policy information about studies involving animals; ARRIVE guidelines recommended for reporting animal research

Laboratory animals	*For laboratory animals, report species, strain, sex and age OR state that the study did not involve laboratory animals.*
Wild animals	*Provide details on animals observed in or captured in the field; report species, sex and age where possible. Describe how animals were caught and transported and what happened to captive animals after the study (if killed, explain why and describe method; if released, say where and when) OR state that the study did not involve wild animals.*
Field-collected samples	*For laboratory work with field-collected samples, describe all relevant parameters such as housing, maintenance, temperature, photoperiod and end-of-experiment protocol OR state that the study did not involve samples collected from the field.*
Ethics oversight	*Identify the organization(s) that approved or provided guidance on the study protocol, OR state that no ethical approval or guidance was required and explain why not.*

Note that full information on the approval of the study protocol must also be provided in the manuscript.

Human research participants

Policy information about studies involving human research participants

Population characteristics	*Describe the covariate-relevant population characteristics of the human research participants (e.g. age, gender, genotypic information, past and current diagnosis and treatment categories). If you filled out the behavioural & social sciences study design questions and have nothing to add here, write "See above."*
Recruitment	*Describe how participants were recruited. Outline any potential self-selection bias or other biases that may be present and how these are likely to impact results.*
Ethics oversight	*Identify the organization(s) that approved the study protocol.*

Note that full information on the approval of the study protocol must also be provided in the manuscript.

Clinical data

Policy information about clinical studies

All manuscripts should comply with the ICMJE guidelines for publication of clinical research and a completed CONSORT checklist must be included with all submissions.

Clinical trial registration	*Provide the trial registration number from ClinicalTrials.gov or an equivalent agency.*
Study protocol	*Note where the full trial protocol can be accessed OR if not available, explain why.*
Data collection	*Describe the settings and locales of data collection, noting the time periods of recruitment and data collection.*
Outcomes	*Describe how you pre-defined primary and secondary outcome measures and how you assessed these measures.*

Dual use research of concern

Policy information about dual use research of concern

Hazards

Could the accidental, deliberate or reckless misuse of agents or technologies generated in the work, or the application of information presented in the manuscript, pose a threat to:

No	Yes	
☐	☐	Public health
☐	☐	National security
☐	☐	Crops and/or livestock
☐	☐	Ecosystems
☐	☐	Any other significant area

Experiments of concern

Does the work involve any of these experiments of concern:

No	Yes	
☐	☐	Demonstrate how to render a vaccine ineffective
☐	☐	Confer resistance to therapeutically useful antibiotics or antiviral agents
☐	☐	Enhance the virulence of a pathogen or render a nonpathogen virulent
☐	☐	Increase transmissibility of a pathogen
☐	☐	Alter the host range of a pathogen
☐	☐	Enable evasion of diagnostic/detection modalities
☐	☐	Enable the weaponization of a biological agent or toxin
☐	☐	Any other potentially harmful combination of experiments and agents

ChIP-seq

Data deposition

☐ Confirm that both raw and final processed data have been deposited in a public database such as GEO.

☐ Confirm that you have deposited or provided access to graph files (e.g. BED files) for the called peaks.

Data access links<br>*May remain private before publication.*	*For "Initial submission" or "Revised version" documents, provide reviewer access links. For your "Final submission" document, provide a link to the deposited data.*
Files in database submission	*Provide a list of all files available in the database submission.*
Genome browser session<br>(e.g. UCSC)	*Provide a link to an anonymized genome browser session for "Initial submission" and "Revised version" documents only, to enable peer review. Write "no longer applicable" for "Final submission" documents.*

Methodology

Replicates	*Describe the experimental replicates, specifying number, type and replicate agreement.*
Sequencing depth	*Describe the sequencing depth for each experiment, providing the total number of reads, uniquely mapped reads, length of reads and whether they were paired- or single-end.*
Antibodies	*Describe the antibodies used for the ChIP-seq experiments; as applicable, provide supplier name, catalog number, clone name, and lot number.*
Peak calling parameters	*Specify the command line program and parameters used for read mapping and peak calling, including the ChIP, control and index files used.*
Data quality	*Describe the methods used to ensure data quality in full detail, including how many peaks are at FDR 5% and above 5-fold enrichment.*
Software	*Describe the software used to collect and analyze the ChIP-seq data. For custom code that has been deposited into a community repository, provide accession details.*

Flow Cytometry

Plots

Confirm that:

☒ The axis labels state the marker and fluorochrome used (e.g. CD4-FITC).

☒ The axis scales are clearly visible. Include numbers along axes only for bottom left plot of group (a 'group' is an analysis of identical markers).

☒ All plots are contour plots with outliers or pseudocolor plots.

☒ A numerical value for number of cells or percentage (with statistics) is provided.

Methodology

Sample preparation	After viral infection cells were washed twice with PBS and fixed with 4% paraformaldehyde for 1 hour at room temperature. After washing with PBS, cells were suspended in Flow Cytometry Staining Buffe and analyzed by Flow Cytometry.
Instrument	LSR Fortessa
Software	FlowJo
Cell population abundance	Cell population abundance was performed when quantifying GFP positive cells. A customised gate was drawn based on the negative control (uninfected cells) and then applied to all samples of the experiment.
Gating strategy	Cells were first gated on FSC-A vs SSC-A to exclude cellular debris. Then, single cells were gated on GFP vs SSC-A. The gating strategy is shown in Supplementary Fig. 2.

☒ Tick this box to confirm that a figure exemplifying the gating strategy is provided in the Supplementary Information.

Magnetic resonance imaging

Experimental design

Design type	*Indicate task or resting state; event-related or block design.*
Design specifications	*Specify the number of blocks, trials or experimental units per session and/or subject, and specify the length of each trial or block (if trials are blocked) and interval between trials.*
Behavioral performance measures	*State number and/or type of variables recorded (e.g. correct button press, response time) and what statistics were used to establish that the subjects were performing the task as expected (e.g. mean, range, and/or standard deviation across subjects).*

Acquisition

Imaging type(s)	*Specify: functional, structural, diffusion, perfusion.*
Field strength	*Specify in Tesla*
Sequence & imaging parameters	*Specify the pulse sequence type (gradient echo, spin echo, etc.), imaging type (EPI, spiral, etc.), field of view, matrix size, slice thickness, orientation and TE/TR/flip angle.*
Area of acquisition	*State whether a whole brain scan was used OR define the area of acquisition, describing how the region was determined.*

Diffusion MRI ☐ Used ☐ Not used

Preprocessing

Preprocessing software	*Provide detail on software version and revision number and on specific parameters (model/functions, brain extraction, segmentation, smoothing kernel size, etc.).*
Normalization	*If data were normalized/standardized, describe the approach(es): specify linear or non-linear and define image types used for transformation OR indicate that data were not normalized and explain rationale for lack of normalization.*
Normalization template	*Describe the template used for normalization/transformation, specifying subject space or group standardized space (e.g. original Talairach, MNI305, ICBM152) OR indicate that the data were not normalized.*
Noise and artifact removal	*Describe your procedure(s) for artifact and structured noise removal, specifying motion parameters, tissue signals and physiological signals (heart rate, respiration).*

Volume censoring	*Define your software and/or method and criteria for volume censoring, and state the extent of such censoring.*

Statistical modeling & inference

Model type and settings	*Specify type (mass univariate, multivariate, RSA, predictive, etc.) and describe essential details of the model at the first and second levels (e.g. fixed, random or mixed effects; drift or auto-correlation).*
Effect(s) tested	*Define precise effect in terms of the task or stimulus conditions instead of psychological concepts and indicate whether ANOVA or factorial designs were used.*

Specify type of analysis: ☐ Whole brain ☐ ROI-based ☐ Both

Statistic type for inference (See Eklund et al. 2016)	*Specify voxel-wise or cluster-wise and report all relevant parameters for cluster-wise methods.*
Correction	*Describe the type of correction and how it is obtained for multiple comparisons (e.g. FWE, FDR, permutation or Monte Carlo).*

Models & analysis

n/a	Involved in the study
☐	☐ Functional and/or effective connectivity
☐	☐ Graph analysis
☐	☐ Multivariate modeling or predictive analysis

Functional and/or effective connectivity	*Report the measures of dependence used and the model details (e.g. Pearson correlation, partial correlation, mutual information).*
Graph analysis	*Report the dependent variable and connectivity measure, specifying weighted graph or binarized graph, subject- or group-level, and the global and/or node summaries used (e.g. clustering coefficient, efficiency, etc.).*
Multivariate modeling and predictive analysis	*Specify independent variables, features extraction and dimension reduction, model, training and evaluation metrics.*
