# Supplementary material for: The CRL5–SPSB3 ubiquitin ligase targets nuclear cGAS for degradation
Source: Nature. 2024 Feb 28;627(8005):873–9. doi: 10.1038/s41586-024-07112-w (PMC10972748; doi:10.1038/s41586-024-07112-w)
Supplement: Supplementary file 3 — Additional information about the purification of the SPSB3–ELOBC complex. [file 41586_2024_7112_MOESM3_ESM.pdf]

Supplementary Note 1

Assessing the purified SPSB3-ELOBC complex by Coomassie blue staining, we noted that a protein of ~ 35 kDa co-eluted with SPSB3, which was competed off by cGAS (See asterisk below and figure panels in Fig. 2d, Extended Data Fig. 4a, b, Extended Data Fig. 5c, and Extended Data Fig. 6a). Mass spectrometry identified the protein as the kanamycin-resistance protein (KanR (see below)). When using an alternative antibiotic resistance gene to express SPSB3 in bacteria, our attempts to purify the SPSB3-ELOBC complex failed. Probably, through engaging with SPSB3, KanR stabilises SPSB3 and prevents protein aggregation. Critically, since the binding of KanR to SPSB3 can be potently outcompeted by cGAS, the presence of KanR does not confound the usage of the SPSB3-ELOB/C complex in further downstream experiments.

a

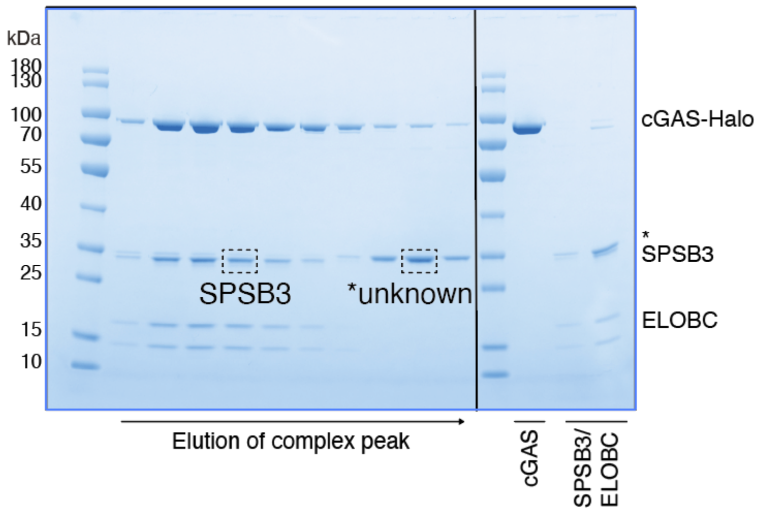
kDa
180
130
100
70
55
40
35
25
15
10
cGAS-Halo
*
SPSB3
ELOBC
SPSB3
*unknown
Elution of complex peak
cGAS
SPSB3/
ELOBC

b

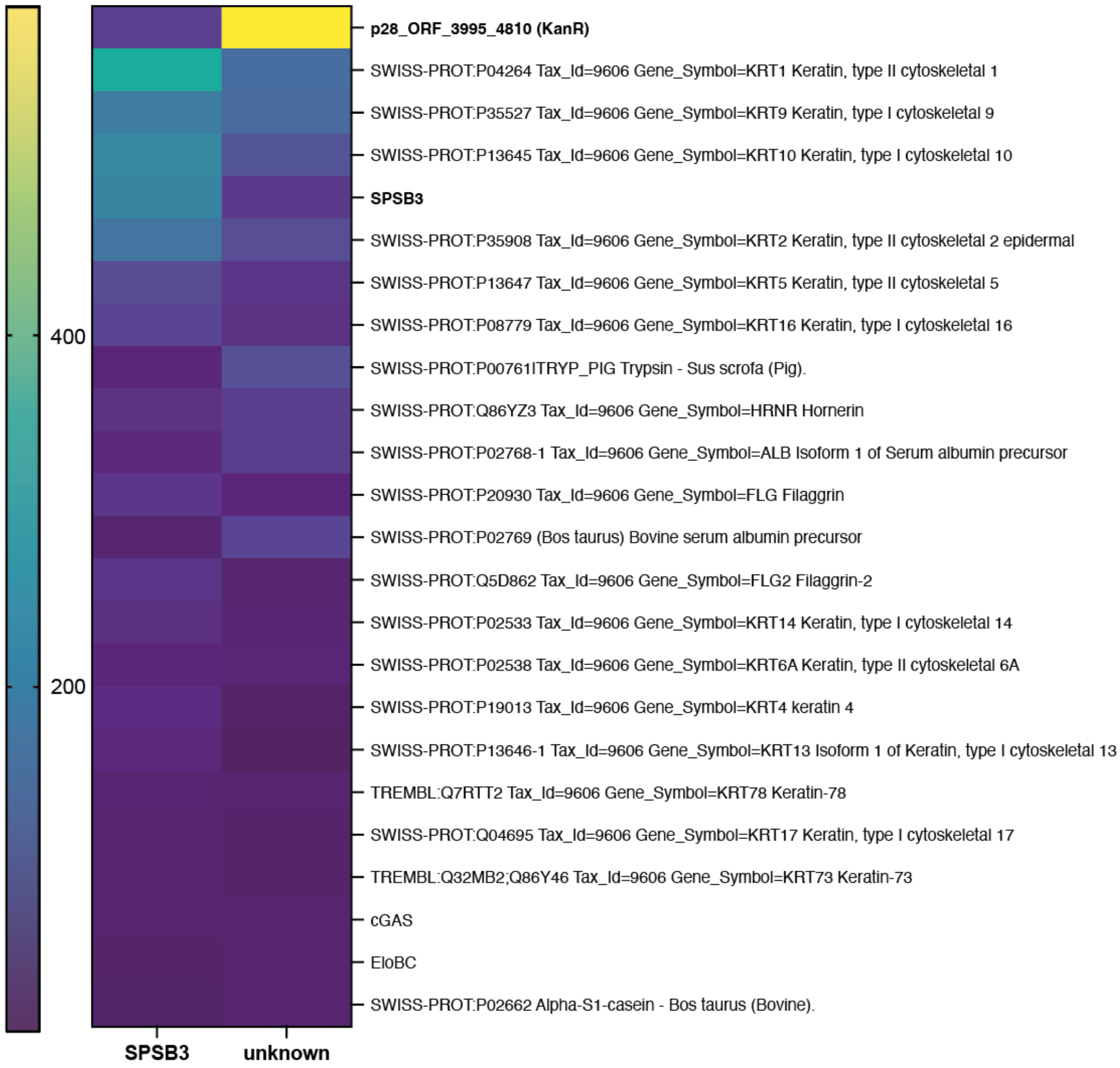
400
200
p28_ORF_3995_4810 (KanR)
SWISS-PROT:P04264 Tax_Id=9606 Gene_Symbol=KRT1 Keratin, type II cytoskeletal 1
SWISS-PROT:P35527 Tax_Id=9606 Gene_Symbol=KRT9 Keratin, type I cytoskeletal 9
SWISS-PROT:P13645 Tax_Id=9606 Gene_Symbol=KRT10 Keratin, type I cytoskeletal 10
SPSB3
SWISS-PROT:P35908 Tax_Id=9606 Gene_Symbol=KRT2 Keratin, type II cytoskeletal 2 epidermal
SWISS-PROT:P13647 Tax_Id=9606 Gene_Symbol=KRT5 Keratin, type II cytoskeletal 5
SWISS-PROT:P08779 Tax_Id=9606 Gene_Symbol=KRT16 Keratin, type I cytoskeletal 16
SWISS-PROT:P00761ITRYP_PIG Trypsin - Sus scrofa (Pig).
SWISS-PROT:Q86YZ3 Tax_Id=9606 Gene_Symbol=HRNR Hornerin
SWISS-PROT:P02768-1 Tax_Id=9606 Gene_Symbol=ALB Isoform 1 of Serum albumin precursor
SWISS-PROT:P20930 Tax_Id=9606 Gene_Symbol=FLG Filaggrin
SWISS-PROT:P02769 (Bos taurus) Bovine serum albumin precursor
SWISS-PROT:Q5D862 Tax_Id=9606 Gene_Symbol=FLG2 Filaggrin-2
SWISS-PROT:P02533 Tax_Id=9606 Gene_Symbol=KRT14 Keratin, type I cytoskeletal 14
SWISS-PROT:P02538 Tax_Id=9606 Gene_Symbol=KRT6A Keratin, type II cytoskeletal 6A
SWISS-PROT:P19013 Tax_Id=9606 Gene_Symbol=KRT4 keratin 4
SWISS-PROT:P13646-1 Tax_Id=9606 Gene_Symbol=KRT13 Isoform 1 of Keratin, type I cytoskeletal 13
TREMBL:Q7RTT2 Tax_Id=9606 Gene_Symbol=KRT78 Keratin-78
SWISS-PROT:Q04695 Tax_Id=9606 Gene_Symbol=KRT17 Keratin, type I cytoskeletal 17
TREMBL:Q32MB2;Q86Y46 Tax_Id=9606 Gene_Symbol=KRT73 Keratin-73
cGAS
EloBC
SWISS-PROT:P02662 Alpha-S1-casein - Bos taurus (Bovine).
SPSB3
unknown
