# Supplementary material for: The CRL5–SPSB3 ubiquitin ligase targets nuclear cGAS for degradation
Source: Nature. 2024 Feb 28;627(8005):873–9. doi: 10.1038/s41586-024-07112-w (PMC10972748; doi:10.1038/s41586-024-07112-w)
Supplement: Supplementary file 4 — Raw data siRNA screen. [file 41586_2024_7112_MOESM4_ESM.pdf]

Supplementary Table 1

Gene symbol	Integrated intensity	SD Integrated intensity	Mean intensity replicate #1	Mean intensity replicate #2	Mean Intensity replicate #3
ABI2	-0.1353486	0.07333365	0.23634284	0.17762523	0.20060125
ACTB	0.29674608	0.14051126	0.60273157	0.3147081	0.46524515
ACTG1	-0.1197244	0.03339002	0.28677521	0.04369244	0.09203009
ACVR1B	-0.1232597	0.12778742	0.28371179	0.06029295	0.03813195
AGL	0.0770728	0.14425308	0.54466943	0.11142112	0.72913728
AICDA	-0.1805802	0.37782721	-0.0996562	0.83751547	-0.1365064
AIRE	0.23092172	0.86250328	0.33265368	-0.358365	2.73216143
AKT1	0.0350233	0.07566417	0.32037481	0.1945998	0.60334332
AKTIP	-0.1011097	0.1766488	-0.1494485	0.48019359	0.0919608
AMBRA1	-0.1388043	0.16569565	0.06405397	0.14060182	0.33475497
AMFR	0.00329455	0.04884928	0.28584414	0.14911603	0.5592733
ANAPC1	-0.0156833	0.21587624	0.23087584	0.00590024	0.38836506
ANAPC10	0.12201365	0.08754367	0.63000034	0.32865129	0.33965955
ANAPC11	0.05020331	0.22850155	0.51918836	0.26710473	0.42405987
ANAPC13	0.117685	0.3314263	0.98013707	0.07364872	0.50601401
ANAPC15	0.20071807	0.29552725	0.94392165	0.15165175	0.56519015
ANAPC16	-0.1862446	0.07433164	0.04472513	-0.0206337	0.47555631
ANAPC2	0.19509695	0.46752103	1.22361157	0.18890516	0.2797315
ANAPC4	0.12062941	0.35879221	0.55474929	0.4034399	-0.1441268
ANAPC5	0.38692096	0.06151379	0.45189926	0.53274255	0.38791843
ANAPC7	-0.2593761	0.34716102	0.26291945	-0.3097664	0.47073115
ANKIB1	-0.0637646	0.22541613	-0.0220923	0.53685741	0.23174237
APC2	-0.1072772	0.24612619	0.41004813	0.07421082	0.12839133
AREL1	0.00032171	0.14865209	0.16262813	0.18212994	0.60355225
ARIH1	-0.1132477	0.07006814	0.11278617	0.17874434	0.28317403
ARIH2	-0.0172727	0.25142473	0.05422913	0.66301079	0.47217216
ARRB1	0.02723005	0.26751293	0.18336942	-0.0297878	0.75564693
ARRB2	0.01525062	0.03558122	0.42962601	0.29538221	0.18867091
ARRDC3	-0.261985	0.01989072	0.10238868	-0.230759	0.05138907
ARRDC4	0.15838309	0.5716852	-0.0312118	-0.0430398	0.37089891
ASB1	-0.3700937	0.05439684	0.05401291	-0.3177554	-0.0785426
ASB10	-0.1193979	0.20148452	0.04243394	0.2038722	-0.2227137
ASB11	0.06270773	0.24282235	0.46383124	0.01738498	0.73929191
ASB12	0.00788568	0.1605601	-0.1346616	0.16461126	0.72342727
ASB13	-0.266561	0.17310439	-0.4128828	-0.0519155	0.51870575
ASB14	-0.1825089	0.17158816	0.08669989	-0.0181709	0.22902414
ASB15	-0.116603	0.09767408	-0.0533528	0.2702801	0.52726177
ASB16	0.213063	0.59164954	1.08287029	0.14700637	0.07715946
ASB17	-0.294264	0.20391949	-0.2665768	-0.2437867	0.5670913
ASB18	-0.1508423	0.19720887	-0.030658	-0.0325208	0.3773499
ASB2	-0.1228122	0.15316065	0.17236198	0.71425893	0.08396564
ASB3	-0.1906241	0.15720359	0.22291796	0.00937434	0.25143243
ASB4	-0.2859605	0.19972499	0.41093715	-0.1049189	0.06686876
ASB5	-0.0774331	0.28285706	0.51843944	0.02437047	0.33629034
ASB6	-0.0848903	0.09705854	-0.0202191	0.05221727	0.64742321
ASB7	-0.1308836	0.11604185	-0.0608425	0.22617214	0.51679776
ASB8	-0.0860142	0.03991779	0.0643334	0.14102977	0.52916623
ASB9	-0.1619383	0.0972599	-0.0390909	0.21675694	0.31996073
ATG3	0.18332458	0.31102194	-0.038254	1.10613271	0.86334757
ATG7	-0.0899735	0.34381096	0.28657745	-0.0517254	0.29572756
ATP6V0C	-0.28635	0.19096462	0.32914923	-0.0702884	-0.2829134
ATRIP	0.08299124	0.46569842	0.24384605	1.29353377	0.051371
ATRX	-0.0059926	0.13205782	0.15872766	0.3857884	0.83484526
AURKA	0.31317606	0.43294643	0.47761129	0.48905317	0.05159224
AXIN1	-0.3168267	0.21412866	-0.4310407	0.02624318	0.22680533
AXIN2	-0.2947809	0.21808011	-0.3345336	0.06273381	0.18016929
B2M	-0.1284159	0.2656376	0.83208756	-0.3770481	0.20683061
BAG5	-0.2851631	0.05270052	-0.0627857	-0.1411149	0.26570468
BARD1	0.18116244	0.39004869	0.31739539	-0.0486656	0.76549038
BAT3	-0.1135619	0.14325775	-0.0343225	0.22562311	0.2607026
BCL10	-0.2342269	0.01096327	0.09914265	-0.0088993	0.20183604
BCL2	0.054124	0.05639969	0.40941977	0.37657093	0.49565186
BCOR	0.08567507	0.2909113	0.45287371	1.27413347	0.38235688
BFAR	-0.1207576	0.44467314	0.41720457	-0.113995	0.28118916
BID	-0.1456558	0.15923699	0.48330304	-0.0901983	0.14815267
BIRC2	-0.2107876	0.43499383	-0.0415874	0.69042532	-0.2274451
BIRC3	-0.1061303	0.16445425	0.04149309	0.33809528	0.2675067
BIRC5	0.58323903	0.71917924	0.1522718	0.13386894	1.00369581
BIRC6	0.12063765	0.19359724	0.22903296	0.75159947	0.61719592
BIRC7	-0.1430645	0.22128803	0.20751281	-0.0055992	0.12458321
BIRC8	-0.0767404	0.22979637	-0.1699498	0.05605367	0.66618085
BLMH	-0.3219891	0.21456626	-0.2192349	-0.3612644	0.52957007
BLZF1	-0.1717851	0.1829923	0.49508249	0.07010653	0.19915593
BMI1	0.07205649	0.40917669	0.29020545	0.08272429	0.7617163
BRAP	-0.4619829	0.29272415	-0.4707083	0.11066263	-0.6461106
BRCA1	0.05235492	0.43758422	0.7907664	-0.2005844	0.523736
BRCA2	-0.011139	0.0938762	0.41348706	0.24463102	0.28449738
BRE	-0.1556519	0.07919454	0.06607354	0.08600996	0.2930063
BTBD1	-0.0312019	0.1428037	0.42842104	0.36980028	0.48882541
BTBD2	-0.0422735	0.29188405	0.00428596	0.32501139	0.07506967

Gene symbol	Integrated intensity	SD Integrated intensity	Mean intensity replicate #1	Mean intensity replicate #2	Mean Intensity replicate #3
BTRC	-0.1435981	0.20577451	-0.119872	0.11497097	0.63546572
BUB1B	-0.0294401	0.13674152	0.233451	0.40348998	0.3933606
BUB3	-0.2893069	0.08261323	-0.238504	-0.3026922	0.25091034
C12orf51	-0.1860801	0.18105332	0.07448243	0.00134367	0.31560795
C14orf100	-0.287276	0.23133483	0.41439663	-0.3044385	-0.1093691
C1orf124	-0.0391985	0.42645456	-0.1874205	1.03212594	0.10414313
C1orf86	-0.039534	0.28051138	0.35067159	0.03673535	0.41408385
C2orf37	-0.3301209	0.35838756	0.13650561	-0.6234317	0.08395738
C6orf106	-0.201829	0.19181118	-0.044921	-0.1238406	0.4509735
CACUL1	-0.0364276	0.33810578	0.58937856	-0.0862622	0.42131462
CALR	-0.006117	0.0910995	0.57401412	0.07415572	0.44195932
CAND1	0.27840217	0.21023347	0.33763642	0.84555913	0.47535558
CAND2	-0.182705	0.20641395	-0.2669765	0.1350956	0.3876793
CASC3	-0.0254657	0.11744539	0.14463053	0.05683881	0.32312006
CASP10	0.18305266	0.22591576	0.20154412	0.71046088	0.38520091
CASP8	0.22311338	0.74617188	1.95853241	-0.1508282	0.6076171
CBFB	0.03051709	0.24866045	0.5737579	0.03282079	0.73355901
CBL	-0.0518984	0.09113573	0.32947266	0.15784425	0.38541939
CBLB	-0.0622785	0.17498236	0.3735436	0.14368484	0.95035568
CBLC	-0.2348601	0.0950654	-0.2452328	-0.0584964	0.01486559
CBLL1	-0.6297274	0.12292699	-0.2708084	-0.422638	-0.5147548
CBS	-0.2343863	0.11279995	0.18559978	-0.1068452	0.19794331
CBX8	-0.3294115	0.23247617	0.11819408	-0.1914639	0.09756316
CCDC22	-0.0139027	0.16911939	0.46586845	0.20502776	0.30014452
CCDC23	-0.1483941	0.34482373	0.47421992	-0.1741789	-0.0867157
CCDC50	-0.0674549	0.13972432	0.2503563	0.28677629	0.12865447
CCNB1	0.07940546	0.06516012	0.44236272	0.10365698	0.14836235
CCNB1IP1	-0.1746499	0.15627601	0.09989566	0.30741743	0.03121531
CCNC	-0.5611243	0.1113204	-0.2063106	-0.6285113	-0.7168044
CCNF	0.04368757	0.48068849	-0.0008557	0.21457562	1.27912763
CCT2	0.17208121	0.30040513	0.62406506	0.17592949	1.00020107
CD40	-0.235882	0.25818759	0.51394563	-0.365231	0.28703655
CDC14B	-0.1946394	0.11995256	0.13637214	0.14959838	0.08147948
CDC16	0.24930062	0.40662122	0.14349493	0.29757338	0.82760156
CDC2	0.50764657	0.29662855	0.37088902	0.3761167	1.30860723
CDC20	0.48836513	0.85007173	0.52969536	0.31344944	2.36287855
CDC23	-0.0970553	0.12332192	0.21913801	-0.0056065	0.26330517
CDC26	0.08557256	0.20078384	0.36830448	0.23379834	0.542149
CDC27	0.20005105	0.58806068	0.98689101	0.03902345	0.39101021
CDC34	-0.0837581	0.15944358	0.52731799	-0.0382966	-0.2667794
CDC73	-0.1582065	0.23950565	0.27720395	-0.1457898	0.50168428
CDCA3	0.07069107	0.15540712	0.44541329	0.95942412	0.36035336
CDH1	-0.1418764	0.15399176	0.30327119	-0.0121202	0.73406132
CDK2	-0.0487597	0.28248406	0.33056754	0.54490486	-0.091778
CDK5RAP3	-0.1422168	0.10634684	0.20671897	0.34931584	0.41470215
CDK8	-0.5964314	0.22558529	-0.606791	-0.3084378	-0.9423975
CDKN1A	-0.2387217	0.14020755	0.27757795	-0.2174136	0.05811868
CDKN1B	-0.0495433	0.24517611	0.34712792	-0.1258538	0.87604384
CDKN2A	-0.2739686	0.11471467	0.05737582	-0.3102259	0.03316785
CGRRF1	-0.1746627	0.31959817	0.2652972	-0.1372322	0.0488786
CHD5	-0.1512525	0.06693284	0.1498714	0.18165861	0.27655561
CHEK2	-0.0183851	0.07379531	0.13755582	0.20025499	0.52740984
CHFR	0.03115225	0.27653197	0.02488317	0.59638876	-0.0353634
CISH	-0.1063216	0.13928095	0.3203328	0.00166638	0.25272441
CLU	-0.1397878	0.10125799	0.3510986	0.00651871	0.17647642
CNOT4	-0.1585	0.159104	0.061851	0.099402	0.15986
COMMD1	-0.0579851	0.29289389	0.26220769	-0.1305157	0.94334478
CRBN	-0.2723543	0.20665622	0.06636338	-0.2710973	0.32283046
CRY1	-0.1908756	0.22911017	0.32849119	0.11109461	-0.0794516
CRY2	0.12705197	0.28506819	0.11946186	0.21728658	0.87652273
CTR9	-0.3072299	0.062922	-0.0729143	0.01670689	-0.081653
CUL1	0.24448734	0.57468906	-0.2676737	0.39204467	1.07845249
CUL2	-0.1788935	0.10225681	-0.169028	0.11815303	0.33727989
CUL3	-0.0446773	0.09036462	0.02312586	0.22611985	-0.0179897
CUL4A	-0.0611429	0.057015	0.15198223	0.256022	0.45988981
CUL4B	-0.2482546	0.10664865	0.01241522	0.10338297	-0.0746762
CUL5	0.77244603	0.58720867	0.95509239	0.66774739	1.50967652
CUL7	-0.1357378	0.18036896	0.04630264	-0.2309489	0.37377752
CUL9	-0.0074002	0.09935418	0.38392451	0.08797743	0.58908769
CXCR4	-0.0765054	0.08554586	-0.0106763	0.0657938	0.43511523
DAXX	-0.2374717	0.19684726	0.16293916	0.06220772	0.01657555
DCAF10	0.10125966	0.43972957	0.07859026	0.0965922	1.59086679
DCAF15	-0.1604958	0.14318592	-0.2223378	0.17473477	0.34840021
DCAF16	-0.340878	0.22480049	0.25736851	-0.3697637	0.04079689
DCAF6	-0.158977	0.22447977	0.16564324	0.23867077	-0.0620793
DCAF7	-0.234312	0.29148326	0.08349038	0.30043564	-0.2103779
DCST1	-0.1184679	0.40037445	0.41532047	-0.1177678	0.11965234
DCUN1D1	0.09123432	0.03210878	0.3029669	0.38568306	0.61059528
DDA1	-0.0189803	0.14350474	0.38633892	0.21442043	0.21507674
DDB1	0.56908406	0.4330935	0.47022751	0.26862178	0.99917666
DDB2	-0.1901679	0.10893998	0.23554415	-0.1349796	0.49988835
DERL1	-0.3755558	0.0757653	-0.2454175	-0.1162562	0.10887054

Gene symbol	Integrated intensity	SD Integrated intensity	Mean intensity replicate #1	Mean intensity replicate #2	Mean Intensity replicate #3
DET1	-0.1665632	0.07639588	0.13818186	0.05897805	0.29028179
DIABLO	-0.0506855	0.22382772	0.47265447	0.47870617	0.20385309
DIO2	-0.0279756	0.15857472	0.1718298	0.04493977	0.02637479
DLG3	-0.2889941	0.19810017	-0.2506588	0.04927916	0.32545838
DNAJA1	-0.1013304	0.07132087	0.35630137	0.03874329	0.28596352
DNAJA3	-0.0126974	0.17166172	0.22270976	0.15740471	0.75144541
DNAJC2	-0.2155923	0.28545988	0.06400731	0.00129973	-0.3864058
DNM1L	-0.1593227	0.14487662	0.31422949	0.19083258	0.2068829
DPF1	-0.0937877	0.17858005	0.03612887	-0.0638882	0.12492131
DPF2	-0.2111414	0.21051853	0.12718149	-0.42838	0.46717017
DTL	-0.0546053	0.26059507	-0.1836533	0.00647677	-0.1726954
DTX1	0.02855405	0.27341367	0.4588643	-0.1165611	0.27541735
DTX2	-0.1834671	0.24090228	-0.0758144	-0.0495141	0.8116785
DTX3	-0.0168671	0.13579167	0.20637093	0.21246726	0.56734805
DTX3L	-0.2902988	0.24125889	0.14374381	-0.1970529	0.16026177
DTX4	0.21497886	0.43301102	1.23871977	0.12886078	0.51715146
DYRK2	0.0349522	0.15669588	0.3768146	-0.0879926	0.75471255
DZIP3	-0.1924259	0.19055054	-0.2856	0.16801283	0.04689029
E4F1	-0.0679404	0.19742933	0.08133993	-0.0942204	0.53394448
EGFR	-0.0300243	0.32245072	0.99045847	0.04010289	0.18987026
EGR2	0.00508277	0.07846584	0.39646604	0.17853077	0.66265448
EIF4E2	-0.3690296	0.19867844	0.10453792	-0.2110916	-0.298641
ENC1	-0.1554419	0.09167875	0.07777677	-0.0231791	0.0754744
EPS15	-0.051689	0.13482708	0.37653562	0.02637814	0.58208707
ERCC8	-0.2960235	0.21254639	0.05880112	-0.0770225	-0.0790521
FAF1	0.29331791	0.63820947	0.07233598	0.34106124	1.94038881
FAF2	-0.1076063	0.12098986	0.0887462	-0.0096863	0.61113878
FAM175A	-0.2697129	0.11259675	-0.0371474	-0.0592748	0.0730626
FAM175B	-0.3390343	0.17652494	-0.0743783	-0.0019693	-0.4465292
FANCF	0.12577852	0.1164447	0.45922832	0.33359109	0.75592657
FANCL	0.08514852	0.64631416	0.18167916	1.60742932	-0.1629893
FBXL11	-0.2385006	0.09256046	0.06548193	-0.0278004	-0.1409937
FBXL12	-0.2824928	0.30008356	0.43106443	-0.2659719	0.07118738
FBXL13	-0.2133096	0.06556593	-0.0912192	0.25285757	0.2829428
FBXL14	-0.0549179	0.24770967	0.35083013	0.22148915	0.17664824
FBXL15	-0.189852	0.15247174	0.40889018	0.20680159	-0.0382467
FBXL16	0.04036061	0.46973728	0.07506024	0.4931415	0.11063934
FBXL17	-0.2802454	0.19782784	-0.0972562	0.35028041	-0.091945
FBXL18	-0.34592	0.14562678	-0.0666708	0.2378124	-0.1822083
FBXL19	-0.2231782	0.23918908	0.15757763	0.20539186	-0.0851567
FBXL2	-0.2464447	0.1935198	0.26344924	-0.0801247	-0.2682382
FBXL20	-0.0400435	0.40214198	0.18755271	0.90805684	-0.1061475
FBXL21	-0.2465079	0.1545184	0.0562923	0.2334822	0.05251383
FBXL22	-0.0565333	0.06708651	0.23398643	0.23049564	0.2394595
FBXL3	-0.1728124	0.26926715	0.62505774	-0.1654332	0.36955835
FBXL4	-0.1920416	0.23593075	0.43455899	-0.0449601	0.15618924
FBXL5	0.18397758	0.45467603	0.25736825	0.06128822	1.20956863
FBXL6	-0.1332868	0.13634014	0.10996682	0.02148445	0.55828262
FBXL7	-0.1319447	0.0490935	0.32861955	0.02092942	0.12328357
FBXL8	-0.189445	0.25450928	0.08456254	-0.1634937	0.37016721
FBXO10	-0.1469151	0.18413201	0.21515965	-0.0752897	0.68476995
FBXO11	-0.001698	0.21528752	0.07496052	0.296618	0.70245007
FBXO15	-0.2847771	0.14416621	-0.0252582	-0.0968768	0.25200278
FBXO16	-0.0704807	0.17336356	0.41220566	0.05178029	0.39242328
FBXO17	-0.1112761	0.17296848	0.20646224	0.11777827	0.41269503
FBXO18	0.01123024	0.39198335	0.57836293	0.27974081	0.34156548
FBXO2	-0.1782366	0.05965869	0.22663111	0.07815246	0.2373893
FBXO21	-0.1958365	0.06234576	-0.100351	0.11179378	0.21525463
FBXO22	-0.3019766	0.14480722	-0.1411278	0.09156101	0.06253323
FBXO24	-0.1337914	0.19985837	0.15388587	0.29630136	-0.0203517
FBXO25	0.04894771	0.2345186	0.50319756	0.0291786	0.71856545
FBXO27	-0.2117894	0.0722906	-0.0937963	0.19449191	0.05920946
FBXO28	-0.1925664	0.2364008	0.43104485	0.06898177	-0.109186
FBXO3	-0.2325668	0.07985904	-0.0225282	-0.0295093	0.06046006
FBXO30	-0.0787657	0.19883034	0.1123942	0.63152781	0.04077834
FBXO31	0.00876487	0.24932187	0.11665117	0.26124906	0.3255829
FBXO32	-0.0131328	0.14177323	0.06799467	0.24864244	0.48670972
FBXO33	-0.1364513	0.22972509	0.18936537	-0.1262801	0.70357185
FBXO34	-0.1412382	0.0866293	0.12905301	0.16293399	0.27590421
FBXO36	-0.2137892	0.34903141	-0.1125502	-0.277836	0.63642996
FBXO38	-0.0852978	0.40927173	0.06321087	1.1155137	-0.2590396
FBXO39	0.13753902	0.43772476	0.80490698	0.58887788	0.24427418
FBXO4	-0.0640639	0.41999869	-0.169316	0.03695453	1.12004442
FBXO40	-0.1537118	0.12362104	0.49771655	0.43814952	0.15221213
FBXO41	-0.1546817	0.19594271	-0.1705889	-0.04458	0.30990871
FBXO42	0.00668264	0.22406129	0.64689096	0.49434687	0.13758729
FBXO43	-0.1426185	0.09868111	0.12104789	0.10959345	0.58792687
FBXO44	0.23846576	0.34106949	0.36288791	0.167054	0.68593478
FBXO45	-0.1983245	0.19363615	0.17045997	-0.0168565	0.54139952
FBXO46	-0.1300761	0.11336268	0.39723942	-0.0891534	0.29437273
FBXO47	0.00611177	0.34645055	0.01313693	0.65326788	0.17359794
FBXO48	-0.0504527	0.08813265	-0.1396372	0.52415746	0.30673264

Gene symbol	Integrated intensity	SD Integrated intensity	Mean intensity replicate #1	Mean intensity replicate #2	Mean Intensity replicate #3
FBXO5	0.33590277	0.47296985	-0.1844901	-0.0251598	-0.236194
FBXO6	-0.1782797	0.23708577	0.24754969	-0.0092011	-0.1056607
FBXO7	-0.2156897	0.15374673	0.27885578	-0.0244203	-0.0198133
FBXO8	-0.1426707	0.05062429	0.0922238	-0.0092954	0.28752469
FBXO9	-0.1951726	0.15600007	-0.116061	0.16366249	0.34004588
FBXW10	-0.1451053	0.05927314	0.19335731	0.08281832	0.21505471
FBXW11	-0.0418429	0.23628157	0.029194	0.4345995	0.58617888
FBXW12	-0.1338667	0.09114076	-0.0358587	0.34972892	0.53910067
FBXW2	-0.0358931	0.32689411	0.04646504	0.08546149	0.64404479
FBXW4	-0.0901705	0.26672785	0.32812418	0.13399812	0.0238477
FBXW5	0.14460825	0.63870593	1.4212588	0.06356108	0.417296
FBXW7	-0.2417657	0.07118022	0.20507537	0.11502289	-0.172916
FBXW8	-0.1339941	0.14181706	0.26081565	0.20978797	0.31235351
FBXW9	-0.1373919	0.14408297	0.16406233	0.14607131	-0.0667399
FEM1A	-0.3429155	0.22752997	-0.4193389	-0.0258409	-0.1816206
FEM1B	-0.0982097	0.06774405	0.08290569	0.25963004	0.30464269
FEM1C	-0.2967025	0.04937783	0.08877592	0.02829182	0.08934052
FHIT	-0.1791385	0.10352939	0.4099042	0.02295339	0.21870099
FOXL2	0.03294957	0.20286116	0.01257262	0.36742556	0.34626079
FOXO1	-0.3114337	0.1267475	-0.03198	-0.1051889	0.2754303
FZD4	-0.3400051	0.27116611	-0.0445126	0.16901896	-0.34718
FZD5	0.10884732	0.36695846	-0.0142244	0.09067612	0.93919365
FZD6	-0.3612759	0.33958548	0.01034328	0.08276699	-0.6228996
FZD8	-0.0882459	0.1097637	0.16536792	0.16034825	0.05840558
FZR1	-0.051202	0.07420401	0.29793077	0.47038185	0.2193472
GAN	-0.0678053	0.01691325	0.12555445	0.11834634	0.06193041
GAPDH	-0.0915283	0.22849631	0.27442773	-0.0611832	0.23692007
GGN	-0.0828637	0.35773838	0.03114268	0.07455638	0.95152145
GLMN	0.06216446	0.33212172	0.60673012	0.04552541	0.50487826
GPR37	-0.1524236	0.2408425	0.46511995	-0.2752131	0.05798597
GRIK2	-0.0194196	0.14950003	0.20061025	0.00857974	0.75488379
GSK3B	0.14965416	0.44262783	0.4141381	0.15247898	1.29652964
GTPBP4	-0.2161764	0.12695584	0.22189415	0.19661503	0.1199254
HACE1	0.13003076	0.51133969	-0.166526	1.40043256	0.81436805
HAMP	-0.2034088	0.06785461	0.09011915	0.30319952	0.06106312
HDAC6	0.03681941	0.40397828	0.16882334	0.10671777	1.08832806
HECTD1	-0.0576734	0.14459391	0.48689641	0.13187528	0.29876765
HECTD2	-0.1049021	0.32772477	-0.0489697	-0.0601429	0.710662
HECTD3	0.03543614	0.25526817	0.60757555	0.25418201	0.14103895
HECW1	-0.0279423	0.04923303	0.24733669	0.12213537	0.5247407
HECW2	-0.1466551	0.04704299	0.11560971	0.25874858	0.36759402
HERC1	-0.073544	0.33987333	0.0431987	0.37511345	-0.0075343
HERC2	0.13791511	0.35798672	0.65757356	0.08189556	0.54478042
HERC3	-0.3722343	0.13591273	-0.1146053	-0.0833982	-0.1025734
HERC4	-0.2087767	0.03010706	0.13721317	0.02332506	0.28735101
HERC5	-0.2266378	0.25367126	0.39159939	-0.2230588	0.34812406
HERC6	-0.075238	0.61172312	0.72806006	-0.0798228	-0.0821415
HFE	-0.0813798	0.20265377	0.50128033	-0.0646625	0.34889916
HIF1A	-0.191981	0.19389276	0.3945367	-0.0330343	-0.1433236
HLTF	-0.2630945	0.02451375	0.02866256	-0.0507819	0.0376067
HM13	-0.1849374	0.07160897	0.05768188	0.15359051	0.21158851
HPRT1	-0.3578736	0.08923489	-0.1334662	-0.1951615	0.01020377
HSPA1A	-0.0980212	0.27523422	0.2334384	0.34549686	0.15399308
HSPA1B	-0.2234675	0.16784125	0.26407399	-0.0434414	-0.0533595
HSPA5	-0.2353182	0.18261128	-0.1045835	-0.284241	0.30686453
HSPA8	-0.2390147	0.32664292	0.00927679	-0.1408053	-0.6509758
HSPA9	-0.2748257	0.25377328	-0.0468994	-0.3099879	-0.4199004
HSPB1	0.28204426	0.71081974	1.14036157	0.24767673	0.18332183
HSPBP1	-0.1360178	0.1887801	0.27718864	-0.0695436	0.52222842
HSPD1	-0.029888	0.29317164	0.32494473	-0.103749	-0.0314997
HUWE1	-0.1905237	0.12780364	0.01353386	0.16768843	0.23533816
IDE	-0.2213513	0.15985585	0.1858336	-0.2340368	-0.2610242
IKBKG	-0.0858044	0.13412942	0.12186776	0.19502224	0.27826037
IRAK1	-0.3972893	0.06772776	-0.2081391	-0.322898	0.04108309
IRF2BP1	-0.0697855	0.08069936	0.13755428	0.15885666	0.38870041
IRF2BPL	-0.237545	0.06614576	0.10217723	0.21057379	0.25742379
ITCH	-0.3782798	0.17866489	-0.5294555	0.10740678	0.07656683
KBTBD5	-0.0941875	0.49142021	-0.2719804	0.30076988	0.06096232
KBTBD7	0.36135636	0.8522789	0.47136491	2.17214297	0.03971815
KCNH2	-0.304347	0.22394098	0.15868286	-0.5204901	0.06691732
KCNQ3	0.12063419	0.3088855	0.35882855	-0.0204632	0.7921513
KCTD10	0.1384847	0.38713262	0.67683712	0.61023436	0.10172719
KCTD11	-0.2347153	0.076057	-0.0975125	-0.0036147	0.06596111
KCTD13	-0.179635	0.19996833	-0.0904163	0.22007431	0.58231256
KDM2B	-0.0298321	0.39990853	0.84782818	0.01367771	0.00705094
KDM4A	-0.0662527	0.38621944	0.39065036	-0.1287359	0.11704864
KEAP1	-0.2401153	0.18170246	-0.2482464	0.07435518	-0.0743288
KIAA0776	-0.1286279	0.16193934	0.38561156	0.09453144	0.13889882
KIAA1333	-0.1421585	0.22508233	-0.0604272	-0.1089987	0.615535
KIAA1530	-0.1137523	0.16517856	-0.0342131	0.43232339	-0.0465239
KIF18A	0.15171798	0.38435121	0.50247472	0.4507259	0.07871398
KLHL10	0.00647974	0.42809256	0.21371165	0.03629784	1.26769379

Gene symbol	Integrated intensity	SD Integrated intensity	Mean intensity replicate #1	Mean intensity replicate #2	Mean Intensity replicate #3
KLHL12	-0.0144102	0.41714867	-0.3264582	0.67050871	0.46004835
KLHL13	-0.0589574	0.11928812	0.15151673	0.34535552	0.21383867
KLHL15	-0.1907362	0.24996514	-0.3361095	0.74704781	0.10301221
KLHL17	-0.2254232	0.20114238	0.18058574	-0.2211704	0.17431222
KLHL2	-0.0056457	0.10748309	0.51901271	0.10817396	0.3227433
KLHL20	-0.230161	0.14493587	-0.2226554	0.03406209	0.00961077
KLHL21	0.3650807	0.26361931	0.94197437	0.32774991	0.70658842
KLHL22	-0.1781878	0.17712133	0.12186958	-0.1132704	0.1255468
KLHL24	-0.3706944	0.08293339	-0.2661723	-0.1604558	0.13110486
KLHL25	0.02534137	0.30553547	-0.276104	0.77121663	0.44831199
KLHL3	-0.1294507	0.12055506	0.19625361	0.00938297	0.51251114
KLHL36	-0.2222669	0.2063425	0.23639736	-0.3721496	0.18341375
KLHL41	-0.031712	0.0142218	0.38496189	0.20125208	0.58797286
KLHL42	-0.4150511	0.00997117	-0.1704447	-0.0905083	-0.1594119
KLHL7	-0.3134348	0.1737087	0.13169937	-0.4135912	0.14769595
KLHL8	-0.3100601	0.11322337	-0.1759344	0.22281096	0.04804189
KLHL9	-0.3771612	0.26196096	-0.311489	0.42852332	-0.1382824
KMT2C	-0.1806906	0.15173588	0.26793131	0.37272882	-0.1540204
LEO1	-0.2888221	0.12536616	-0.0169459	0.06079254	0.0421751
LMO7	-0.0560937	0.0432995	0.28573213	0.02769757	-0.0502401
LNPEP	-0.25792	0.20268	-0.3048	0.333538	0.197873
LNX1	-0.1340801	0.30171157	0.07416563	0.61539147	-0.292078
LNX2	-0.1624916	0.15961458	0.21824395	0.30228495	0.15058431
LOC283116	-0.077287	0.11391179	0.33550233	0.11717836	0.3606027
LONRF1	-0.1212875	0.1507182	0.21429362	0.1040079	-0.0381508
LONRF2	-0.20547	0.24175073	-0.2188518	0.08948092	0.54091221
LONRF3	-0.0593535	0.1047196	-0.1417842	0.36750173	0.02880367
LRR1	-0.1014559	0.18876564	0.01474621	-0.1022616	0.24757955
LRRC41	-0.0614414	0.16434477	0.18508995	0.07958777	-0.0624422
LRRK2	-0.1944541	0.05224665	-0.102925	0.20784635	0.34750252
LRSAM1	0.19780621	0.37100708	0.40192405	0.12640841	1.00805575
LTBR	0.006963	0.121253	0.508432	0.396697	0.450395
LYN	-0.13905	0.332212	-0.02949	0.255834	-0.30081
MAD1L1	-0.2562332	0.10511706	0.11724248	-0.0949864	0.03221376
MAD2L1	0.179678	0.260276	0.606017	0.312243	0.266174
MAD2L2	-0.1141062	0.10198741	-0.189057	0.36488998	0.24518753
MAGEA2	0.352366	0.85047	1.806179	0.211079	0.136623
MAGEA2B	-0.1561076	0.08632147	0.21274704	0.18596694	0.14509507
MAGEC2	0.00075433	0.19932408	0.59836955	0.65217124	0.21621369
MALT1	0.04390659	0.21520122	0.62906674	0.09409884	0.47489111
MAP3K1	-0.05658	0.202513	0.148514	0.226	0.34024
MAP3K7IP2	-0.1595113	0.09544864	0.40000987	0.06514522	0.1152483
MARCH1	-0.0185753	0.34533512	0.21461128	0.10526458	1.14184848
MARCH10	0.09905681	0.24477459	0.68390807	-0.0372033	0.45536676
MARCH11	-0.2262029	0.21662171	-0.0526597	-0.0435694	0.30229451
MARCH2	-0.3760198	0.07768417	0.0550681	0.02754452	0.00969158
MARCH3	0.04841663	0.47479757	-0.2205838	0.7655218	0.35884052
MARCH4	-0.2606582	0.04357116	0.08226746	-0.1341101	0.17187304
MARCH5	-0.177205	0.1354749	0.21242129	0.24380413	0.42232011
MARCH6	-0.0034524	0.4589948	-0.0103662	0.10645257	1.53766532
MARCH7	-0.0274609	0.22473287	0.2205122	0.14999857	0.61032541
MARCH8	-0.0470058	0.21391712	0.22414478	0.37712968	0.24354387
MARCH9	-0.1633662	0.29409347	0.05928825	-0.299784	0.41149579
MARK4	0.06662189	0.174551	0.34064407	0.36495136	0.10964527
MC1R	-0.19056	0.200792	0.272	-0.35245	0.02163
MC4R	-0.03532	0.312693	0.03521	0.225339	0.228578
MDM2	-0.11756	0.297448	-0.13811	0.537483	0.421126
MDM4	-0.24996	0.084556	-0.14378	0.126133	0.213345
MED1	-0.2662	0.107495	-0.06303	-0.01461	0.138945
MED10	-0.1264885	0.1253459	0.09559531	0.46200864	0.10578546
MED11	-0.1739716	0.11618033	-0.0100728	-0.2646175	0.49991153
MED12	-0.4676765	0.17118378	-0.4658823	-0.5010203	-0.6529832
MED17	-0.0935011	0.13380412	-0.074968	0.09732031	0.46319795
MED20	-0.139155	0.06848161	-0.0633856	0.13573259	0.30370552
MED21	-0.0468551	0.11345837	0.03908198	0.24473081	0.22678329
MED23	0.06728343	0.32082867	0.15104879	0.19013958	0.9969531
MED24	-0.4196461	0.1989235	-0.3074495	-0.03775	-0.3973668
MED27	-0.1212566	0.09810914	0.30037658	0.04421585	0.27869495
MED31	0.07715824	0.36138702	0.75212503	0.012426	0.0938953
MED6	-0.482319	0.14638988	-0.4361437	-0.4506616	-0.4761138
MED7	-0.2158397	0.06646741	0.00240361	0.08041111	0.31919436
MED8	-0.2182916	0.14129105	-0.3918757	-0.3437573	0.31679812
MEX3A	-0.2782543	0.23482337	-0.5132837	0.15932594	0.50443333
MEX3B	-0.1494724	0.26123534	-0.1437865	0.72013387	0.18581449
MEX3C	-0.1664921	0.08736862	0.26263496	0.24086785	0.16593872
MEX3D	-0.1488606	0.15886215	0.10748175	0.09080845	0.29043836
MFN2	-0.1364186	0.06270874	0.09312897	0.01540188	0.44667891
MGRN1	-0.0399642	0.20934597	0.36320987	0.41848566	0.02474817
MIB1	-0.1194456	0.14420028	0.43246442	0.32213723	0.05358077
MIB2	-0.0835372	0.19704524	-0.1311635	0.22078068	0.41807778
MID1	-0.05994	0.072253	0.218708	0.197493	0.455407
MID2	-0.0698562	0.24588101	0.52218756	0.07102624	0.16420098

Gene symbol	Integrated intensity	SD Integrated intensity	Mean intensity replicate #1	Mean intensity replicate #2	Mean Intensity replicate #3
MKRN1	-0.3524898	0.01592209	-0.16334	-0.1504911	-0.0811678
MKRN2	-0.1714008	0.16091397	0.23640742	-0.0135369	0.18394698
MKRN3	-0.1912255	0.07632711	-0.1524903	0.07946785	0.14694147
MLL2	-0.0169736	0.15048098	0.32389061	-0.0396289	0.15645456
MNAT1	-0.12514	0.071519	-0.04047	0.277397	0.406334
MOAP1	-0.2424978	0.06777011	0.12612217	0.20357353	0.00410563
MOCS3	-0.2585599	0.27248121	-0.0081405	-0.0036136	-0.3152813
MSL2	-0.166714	0.14055233	0.12697804	0.19782745	0.39493488
MTMR15	-0.1267508	0.08974194	-0.0158617	0.04591098	-0.1628567
MUL1	-0.2624687	0.11294538	-0.0097308	0.10251284	-0.1464558
MVB12A	0.041615	0.24487985	0.4948942	0.08290492	0.61270529
MYCBP2	-0.1459081	0.12029467	0.28574159	0.05389246	-0.0772112
MYLIP	0.18034865	0.42113836	0.7018653	0.52552368	0.14226131
MYOD1	-0.21074	0.089855	-0.05871	0.148854	0.107803
N4BP1	-0.2447075	0.03718293	-0.004757	-0.0556368	0.10932705
NAE1	-0.0059572	0.19372051	0.63180069	-0.0783016	0.18480322
NAIP	-0.02453	0.313504	-0.14883	0.370511	0.522062
NBR1	-0.34803	0.184	-0.42528	0.08671	-0.12113
NDFIP1	-0.0018739	0.32598949	0.17276287	0.58523533	0.09276155
NDFIP2	-0.2635675	0.44736516	0.44315669	-0.1490974	-0.2443766
NEDD4	-0.29245	0.140753	0.031444	0.165261	0.005473
NEDD4L	-0.0535132	0.118967	-0.0477801	0.15996509	0.51786503
NEDD8	0.679765	0.35029	0.786729	1.233098	0.828195
NEURL	-0.1958698	0.09130546	0.21559334	-0.0091243	0.09325718
NEURL1B	-0.1042445	0.19348506	0.45032688	-0.0280368	0.30105712
NEURL2	0.03453454	0.29796726	-0.2655701	0.70785536	0.6223563
NEUROD2	-0.0133	0.288368	0.254259	0.616577	0.301897
NFE2L2	-0.1143	0.113052	0.383381	-0.04422	0.43661
NFKBIA	-0.16902	0.221124	-0.04385	0.12879	0.39186
NFX1	-0.21953	0.172225	0.199423	0.310186	0.144184
NFXL1	-0.2565689	0.19305549	-0.0054815	-0.0986729	0.3214452
NGFR	-0.16777	0.161144	-0.09274	0.323341	0.125088
NHLRC1	-0.1505152	0.24523587	-0.1422612	0.10781597	0.76802146
NKD2	0.04964496	0.23259623	0.64118099	0.20516178	0.30350727
NLK	-0.0831361	0.23401649	0.57636123	-0.0554922	0.3693513
NLRC4	-0.1544633	0.1103988	0.22143847	0.36851711	0.01347089
NOSIP	0.06862161	0.19467563	0.04456446	0.10856966	1.08066557
NPEPPS	-0.0320964	0.11273643	0.15053429	0.20972751	0.56658444
NSD1	0.00672497	0.15029888	0.32968714	0.04913628	0.30569788
NSFL1C	-0.0360871	0.21747149	0.53357521	0.28243292	0.2086008
NSMCE1	-0.3104162	0.23087376	-0.1855133	-0.0744794	0.59238318
NSMCE2	-0.1101773	0.20894798	0.13530038	0.18441725	0.6300042
NUB1	-0.257314	0.05676297	0.084132	0.36982318	0.14059252
NUP62	0.33716214	0.29298478	0.08482998	0.17346106	1.29584919
NXN	-0.2857333	0.27523044	0.21421173	-0.1697089	-0.0532932
OPTN	-0.1459334	0.18997156	0.29531103	0.19177615	-0.0294237
OSTM1	-0.1497841	0.28555396	0.27917855	0.39886337	-0.4251151
PA2G4	-0.35596	0.201141	-0.04015	-0.0916	-0.24004
PACRG	-0.3979888	0.05035352	-0.1843071	-0.1491441	0.02229801
PAF1	-0.2359333	0.22172295	0.41956258	-0.0870926	0.03847078
PARK2	-0.20697	0.044496	-0.01247	0.111029	0.096362
PARK7	-0.0951715	0.1498853	0.45585617	0.07949704	0.02668162
PARP10	-0.1385464	0.39611131	0.13076407	-0.4205911	0.5329195
PATZ1	-0.2089843	0.24578164	0.26086467	0.08283657	-0.1071692
PAX6	-0.2512	0.224636	-0.17082	0.202979	0.032443
PAXIP1	-0.1526373	0.05339881	-0.0542245	0.05362964	0.00399747
PCBP2	-0.27427	0.037435	-0.13535	0.043408	0.034279
PCGF1	-0.0845081	0.0752435	-0.0453819	0.16319113	0.37402907
PCGF2	-0.2627821	0.08335385	0.08601988	0.00366502	0.24121228
PCGF3	-0.0859819	0.34267277	-0.0586165	0.19101464	1.19607019
PCGF5	-0.0815501	0.0698231	0.14718942	0.09807574	0.3536264
PCGF6	-0.1665847	0.01929386	-0.0563077	0.30969098	0.34375801
PCNP	-0.2112114	0.16686732	-0.0344437	0.50699851	0.00285943
PDCL3	0.03382568	0.17373499	0.21172367	0.50384043	0.39732143
PDE4D	-0.10944	0.338405	0.516476	-0.23662	0.87638
PDZD4	0.10647846	0.18281475	0.36472963	0.33745671	0.70687215
PDZRN3	-0.0177594	0.21840283	0.41500685	0.03122358	0.38676458
PDZRN4	-0.2405344	0.17531172	0.33337456	0.0094203	0.00594937
PELI1	-0.1618615	0.0712519	0.12050787	0.23764501	0.2429688
PELI2	-0.3021219	0.24490452	0.03204328	-0.0603417	0.22865242
PELI3	0.03785665	0.27178701	0.48489372	0.0918902	0.24370243
PER1	-0.13077	0.215571	0.127342	0.437947	0.173822
PER2	-0.2334988	0.11998607	-0.136489	0.1952856	-0.0472857
PER3	-0.1276548	0.05895785	0.08840408	0.20234383	0.22252789
PEX10	0.071173	0.15559	0.197715	0.219876	0.950166
PEX12	0.073141	0.14907	0.387983	-0.02559	0.570809
PEX2	-0.16561	0.229313	0.362315	0.058908	-0.36399
PHC1	-0.0398011	0.26262989	0.50578025	-0.1839716	0.46639985
PHF14	-0.1760231	0.03484003	0.10520404	0.0349122	0.24870967
PHF21A	-0.3127798	0.14387198	-0.2067537	0.0455422	0.19144393
PHF7	0.05019946	0.32081174	0.94330593	0.55773394	0.21104688
PHIP	-0.2294895	0.04720756	-0.1181596	-0.2763278	0.10485219

Gene symbol	Integrated intensity	SD Integrated intensity	Mean intensity replicate #1	Mean intensity replicate #2	Mean Intensity replicate #3
PHRF1	-0.0346533	0.08034804	-0.0077939	0.50611642	0.36037843
PIAS1	-0.1202094	0.0597583	0.13129319	0.24469421	-0.0929
PIAS2	0.10691581	0.22684099	0.26689205	0.33394366	0.76145836
PIAS3	-0.1625508	0.09487286	0.20448502	0.08193617	0.22399885
PIAS4	0.08611266	0.07301239	0.44087299	0.55658404	0.71810694
PINK1	0.23664302	0.2017747	0.45825426	0.83915021	0.61463925
PJA1	-0.2572993	0.2335613	-0.270507	0.02823389	0.52644832
PJA2	-0.2179894	0.24119105	0.37804891	0.19145613	-0.1498985
PLK1	1.274836	0.393583	1.574699	1.64081	1.243434
PML	0.05394	0.138287	0.283215	-0.06951	0.74425
POLR2A	-0.42185	0.259079	-0.46786	-0.41289	0.371471
POU5F1	0.003519	0.154905	0.254026	0.339511	0.542774
PPARA	-0.12134	0.167473	-0.16667	0.341383	0.137556
PPARGC1A	-0.0297676	0.11016246	0.4764355	0.19063562	0.07974857
PPIL2	0.24009473	0.31459216	0.60099544	0.13267204	1.10681432
PRICKLE1	-0.042597	0.27196797	0.33693103	0.20583165	0.27760796
PRKACA	-0.09822	0.554315	-0.283	0.056838	1.372864
PRKACB	-0.25061	0.044767	0.069223	-0.03018	0.217085
PRKAR1A	-0.10867	0.345391	-0.38192	0.536176	0.351543
PRKAR2A	-0.20347	0.295877	-0.41506	0.154347	0.385106
PRKAR2B	-0.30973	0.314065	-0.30259	0.168579	-0.25026
PRKCQ	-0.23762	0.157267	-0.19211	0.203374	0.335949
PRMT3	-0.1996981	0.07903485	0.10780119	0.11517952	0.04455127
PRPF19	0.27094501	0.02433482	0.56263024	0.21269165	0.22090701
PSMA1	0.107568	0.316068	0.338429	0.429548	0.311964
PSMA2	0.098242	0.188711	0.294897	0.36488	0.210772
PSMA3	0.458228	0.298049	0.612971	0.192772	1.130059
PSMA4	-0.00314	0.347777	-0.24191	0.190785	0.698813
PSMA5	0.394178	0.447481	0.603101	0.069131	1.024982
PSMA6	0.461976	0.604614	0.409284	0.111667	2.074862
PSMA7	0.049574	0.292492	0.489722	0.107052	0.220349
PSMA8	-0.2000171	0.14117287	-0.1047359	0.04581808	0.47992322
PSMB1	0.239438	0.292527	0.328597	0.281014	0.766596
PSMB10	0.093767	0.388077	0.095279	0.540404	0.405997
PSMB11	-0.1709146	0.13764434	0.14547128	0.29162628	0.03519738
PSMB2	0.082734	0.079304	0.458825	0.354861	0.597143
PSMB3	0.105073	0.227349	0.177611	0.361735	0.440675
PSMB4	0.241668	0.374622	0.383511	0.134248	0.807783
PSMB5	0.24595	0.057327	0.497916	0.460083	0.922509
PSMB6	0.427902	0.9412	0.227807	0.037447	2.234337
PSMB7	0.09232	0.193387	0.441075	0.12602	0.281923
PSMB8	-0.18501	0.050178	0.305019	0.098501	0.358756
PSMB9	-0.09106	0.0944	0.095354	0.079852	0.714389
PSMC1	1.597721	1.076616	3.509806	1.020804	1.968912
PSMC2	1.055021	0.553661	0.63181	1.549906	2.129981
PSMC3	1.177703	0.372407	1.859045	1.094045	1.821123
PSMC4	1.266393	0.489084	1.576626	0.86871	2.387118
PSMC5	1.546048	0.610048	1.897168	0.871911	2.683385
PSMC6	1.242154	0.884064	1.828373	0.402154	2.908569
PSMD1	2.17775	0.238019	2.175038	1.816037	3.008871
PSMD10	-0.02779	0.098252	0.244424	0.221731	0.464923
PSMD11	1.276351	0.547117	1.150305	0.85809	2.131872
PSMD12	1.221809	0.616611	0.814699	0.564291	2.630497
PSMD13	0.334372	0.324961	0.436817	0.226327	0.353087
PSMD2	0.929245	0.943423	0.156846	1.478136	2.398565
PSMD3	1.600412	0.456159	1.184995	2.299582	1.889189
PSMD4	0.317669	0.258594	0.418659	0.17067	0.951105
PSMD5	-0.10857	0.103821	0.16888	0.363131	0.285213
PSMD6	1.55223503	0.3762499	1.35504204	1.06101809	1.90644998
PSMD8	1.882899	1.475375	3.020047	1.184859	0.850128
PSMD9	-0.10527	0.066395	0.072988	0.214474	0.410935
PSME1	-0.23038	0.270007	0.214272	-0.28453	0.212392
PSME2	-0.07291	0.240604	-0.35244	0.379445	0.295881
PSME3	-0.1955146	0.16062338	-0.0087063	0.15185241	-0.2716568
PSME4	-0.0097915	0.28757529	0.2089758	0.05192656	0.84239678
PSMF1	-0.2200394	0.14636191	-0.0891777	-0.2060476	0.38529161
PTTG1IP	-0.1807065	0.3743264	-0.3050877	0.13922906	0.64124693
RAB40A	-0.0812872	0.13149102	0.04495339	0.23477357	0.32308257
RAB40AL	-0.1211773	0.1044954	0.01072946	0.44280152	0.4269805
RAB40B	-0.1739407	0.15790936	0.01138301	-0.0208004	0.51914851
RAB40C	-0.2095815	0.17679896	0.08879867	0.31993081	-0.1814172
RABGEF1	-0.0699652	0.31629632	0.58050901	-0.0487598	0.52597558
RAD18	-0.1348013	0.29568378	0.04339759	0.67883026	-0.2236379
RAD23A	0.41348525	0.95562361	1.54454725	-0.1570473	0.08499686
RAD23B	0.00769406	0.18805933	0.14301758	0.1892528	0.79690296
RAD51	-0.1386196	0.07167964	0.07673236	0.0022949	0.26953374
RAG1	-0.0122251	0.07583968	0.14051366	0.24789457	0.38684789
RAG2	-0.0339642	0.34400104	-0.2386124	0.08249772	1.15083695
RALA	-0.0939431	0.05352387	0.12379601	0.18296534	0.65284576
RALB	0.02968886	0.1313351	0.20150054	0.23539119	0.54639527
RANGAP1	-0.1226516	0.24045841	0.35970967	-0.1414512	0.3359599
RAPSN	0.09500136	0.08486284	0.23284574	0.20173973	0.71909735

Gene symbol	Integrated intensity	SD Integrated intensity	Mean intensity replicate #1	Mean intensity replicate #2	Mean Intensity replicate #3
RASD2	-0.1178752	0.13830088	0.43201646	0.08385074	-0.0886446
RASSF1	-0.0673257	0.02379431	0.29438571	0.19844872	0.29194001
RB1	-0.0442015	0.23205132	0.10099208	0.55454345	0.04385785
RBBP6	-0.0953639	0.27197011	0.39830436	-0.2874501	0.51359538
RBCK1	0.01757941	0.35991668	0.31697998	0.00404349	-0.0420172
RBX1	-0.0951493	0.07456786	0.0228282	-0.1195309	-0.0697974
RC3H1	0.11781823	0.35367309	0.41000289	0.54059406	0.23737449
RC3H2	-0.0919339	0.18410287	0.51891007	0.44564325	0.16016101
RCHY1	-0.1655969	0.03145535	0.13574318	0.1016888	0.31753093
RELA	0.0051059	0.03116645	0.39197063	0.25349611	0.49306079
RFFL	-0.2904998	0.10909307	0.01261111	0.01790495	0.0676067
RFPL1	-0.1278472	0.11224914	-0.1476752	0.23009006	0.285389
RFPL2	-0.241564	0.11036837	-0.0923085	-0.0245702	-0.004115
RFPL3	-0.156124	0.15668662	0.26463053	0.1036744	0.15600691
RFPL4A	-0.1545829	0.09383553	0.04380652	0.18753117	0.45820587
RFPL4B	-0.1354686	0.28218871	0.29941669	-0.2446703	0.27955616
RFWD2	-0.4012775	0.07268265	-0.1438872	-0.098876	0.05717098
RFWD3	-0.074038	0.2935363	-0.1049132	0.57351356	0.03278849
RHOBTB1	-0.3100623	0.13865661	0.1547078	-0.0480891	-0.2901408
RHOBTB3	-0.1446084	0.29346667	0.31648536	-0.1235118	-0.3363753
RING1	0.1145938	0.20576254	0.18089698	0.45365353	0.94306296
RIPK1	-0.0902704	0.11148416	0.05370045	0.09962171	0.38341399
RNF10	-0.1108783	0.17132465	-0.2242204	0.35333979	0.4238899
RNF103	0.14501537	0.39726414	0.1125972	0.18681533	1.17487401
RNF11	-0.0977544	0.03988117	0.1967054	0.10776055	0.40113176
RNF111	-0.3324352	0.25015773	0.25487023	-0.2300133	-0.070513
RNF112	0.04682568	0.15059185	0.49468763	-0.0252237	0.62627595
RNF113A	-0.0883501	0.22707372	0.41504258	0.03580572	0.14929216
RNF113B	-0.2241405	0.30108141	-0.0810043	-0.4064666	0.45046125
RNF114	-0.3551881	0.1762323	-0.1349106	-0.0999548	0.28506087
RNF115	0.06483974	0.51104249	-0.0291948	-0.0803411	0.9970788
RNF12	-0.3756021	0.15618169	-0.0386752	-0.2226828	-0.0366317
RNF121	-0.1783908	0.09507021	0.01022769	0.30302501	0.17242346
RNF122	-0.0577119	0.22243438	0.25863426	0.14603246	0.45451993
RNF123	-0.2393302	0.14864565	0.23879861	0.06738024	0.06827823
RNF125	0.07238138	0.50359805	0.86471318	-0.3637081	0.78571775
RNF126	0.05139491	0.08255523	0.19763956	0.49047631	0.51826571
RNF128	-0.2795406	0.07881389	0.10799618	0.09581421	-0.0525737
RNF13	-0.1842426	0.07879464	0.02901012	-0.0881562	0.13254632
RNF130	-0.3514021	0.06729794	0.08959511	0.11228894	-0.2421116
RNF133	-0.1526768	0.13347905	-0.0124632	0.48445737	0.21252981
RNF135	-0.0249692	0.06015074	0.12871728	0.178683	0.44642734
RNF138	0.19991553	0.08728507	0.60130056	0.96190221	0.96466573
RNF139	-0.152964	0.02799818	0.14313318	0.03796171	0.29267146
RNF14	0.01800552	0.11051513	0.17959307	0.1739949	0.63231592
RNF141	-0.1606646	0.01877165	0.23230933	0.51170826	0.29803014
RNF144A	-0.1738068	0.04521492	-0.0160563	-0.0475604	0.43178479
RNF144B	0.10455715	0.28869092	0.32923463	0.16929581	0.94983092
RNF145	0.04883405	0.17527707	0.19622813	0.68359667	0.34410667
RNF146	-0.0824216	0.25305372	0.45210122	0.18105521	0.11360865
RNF148	-0.0852168	0.42450623	0.40285721	-0.2331086	0.44579202
RNF149	-0.1731807	0.17741613	0.40123252	0.101204	0.24181035
RNF150	-0.1384548	0.08611391	0.0730184	0.65456603	0.48336669
RNF151	-0.079521	0.04291349	0.10226515	0.27479785	0.44988754
RNF152	-0.0345308	0.19665678	0.21810827	0.09921761	0.88627043
RNF157	-0.2486144	0.12451853	0.11332957	-0.0296917	0.39321694
RNF160	-0.2313916	0.24618838	0.45135164	-0.2802959	0.02803808
RNF165	0.01690118	0.19393486	0.46324091	0.08379969	0.27528194
RNF166	-0.2976411	0.06171447	-0.3945034	-0.3307544	0.23742445
RNF167	-0.0834407	0.28673317	0.52394442	0.26813992	0.03752593
RNF168	-0.2227094	0.09210899	0.06534103	-0.0386283	0.1068191
RNF169	-0.1994776	0.1809746	0.01315935	0.04104716	0.07005075
RNF17	-0.371422	0.19284106	-0.0730536	0.07431946	-0.4498217
RNF170	-0.1923899	0.13948424	0.17455414	0.08429325	0.24798866
RNF175	-0.1340014	0.07113389	0.20355448	0.2319612	0.31382062
RNF180	-0.0948555	0.49896106	0.86033395	-0.0289601	0.10823122
RNF181	-0.3630454	0.33781487	-0.4675959	0.19617395	0.58282846
RNF182	-0.098803	0.10275203	0.37681963	0.17369423	0.46327471
RNF183	-0.3537235	0.21797401	-0.4532051	-0.1943855	0.49242059
RNF185	-0.1561537	0.22138696	0.08503529	0.02275427	0.14333975
RNF186	-0.0102298	0.09576096	0.39385928	0.44700846	0.59368932
RNF187	-0.3345729	0.14368503	-0.1704886	-0.0683885	-0.2754812
RNF19A	-0.1911888	0.14259108	0.30918974	-0.1277759	0.15758613
RNF19B	-0.1180977	0.14157038	0.20423726	0.17326534	0.17456267
RNF2	-0.0765789	0.09937163	0.02851724	0.2366421	0.52916297
RNF20	-0.3941527	0.11258728	-0.0154322	-0.0472111	-0.0811475
RNF207	-0.1544772	0.07911034	0.1908553	0.06940544	0.30125637
RNF208	-0.1756593	0.04564243	-0.1382383	0.03869468	0.25048346
RNF212	0.05863117	0.03686471	0.49317137	0.65317627	0.5206346
RNF213	-0.2428924	0.12555323	0.06685753	0.18339351	-0.113024
RNF214	-0.2079034	0.1243646	0.11360225	-0.0684837	0.098513
RNF215	0.40980603	0.95639843	2.14383553	0.48300344	0.04101022

Gene symbol	Integrated intensity	SD Integrated intensity	Mean intensity replicate #1	Mean intensity replicate #2	Mean Intensity replicate #3
RNF216	-0.2113162	0.17153135	0.17652981	0.22482511	0.55652753
RNF217	-0.177792	0.11198436	0.04659272	0.41521147	0.10385561
RNF219	-0.2661552	0.2520173	0.30662472	-0.4670225	-0.042221
RNF220	-0.2068007	0.06965817	0.08799043	0.22245879	0.0952888
RNF222	0.0765893	0.46318474	0.40247691	0.09582258	0.1569524
RNF24	-0.2054406	0.1700075	0.16481147	0.12794298	0.02091528
RNF25	-0.2227955	0.17522706	-0.2246197	0.38058966	-0.0731518
RNF26	-0.1974062	0.23548259	0.3481786	-0.1574178	0.17523634
RNF31	-0.2923284	0.16010623	-0.1495995	-0.3498293	0.12767179
RNF32	-0.2265753	0.08602133	-0.1501647	0.01085293	0.44461299
RNF34	0.00685626	0.30266102	-0.0591225	0.89727875	0.51770391
RNF38	-0.2797856	0.21733956	0.16723244	-0.2137074	0.04763129
RNF39	0.0113494	0.14599526	0.10256517	0.96025354	0.47157763
RNF4	-0.0015878	0.28702509	0.36899576	-0.0135695	1.11517974
RNF40	-0.2065344	0.03440887	-0.0652977	-0.0366644	0.28480088
RNF41	-0.2121907	0.02867329	0.04599323	0.06478878	0.26644458
RNF43	0.05298766	0.34369625	0.60255938	-0.1938018	0.70266042
RNF44	-0.0248113	0.27524265	-0.1175616	0.17696846	0.4595975
RNF5	-0.142447	0.2654785	0.0550458	-0.3266757	0.80515984
RNF6	-0.0264941	0.11551373	0.11993024	0.05352639	0.87513609
RNF7	0.33957279	0.42075158	1.13109262	0.3198789	0.42588172
RNF8	-0.1625199	0.21875382	0.18513959	0.0427257	0.08771452
RNFT1	-0.0276952	0.15540419	0.37151192	0.40365864	0.43531956
RNFT2	-0.0766568	0.42055449	0.59053105	-0.278747	0.18005612
RPA2	-0.0888894	0.01110479	0.06199715	0.07240279	0.27584551
RPL13A	-0.0777124	0.17106435	-0.002392	0.1703694	0.45989168
RPLP0	1.63818124	0.46634545	2.46642764	1.25414135	2.70281137
RPS27A	-0.089955	0.15048687	0.12210543	0.38245682	0.60823354
RSPRY1	-0.0201015	0.1268958	0.08009183	0.50012658	0.3384868
RUSC1	0.14433016	0.41358234	0.70166722	0.3196497	0.07673296
RYBP	-0.1405979	0.35946896	0.44680087	0.37788816	-0.2617185
SAE1	-0.077564	0.12775232	0.05503551	0.27994765	0.71147259
SASH1	-0.1173888	0.14232124	-0.1320032	0.04598479	0.47423309
SCAMP3	-0.2114163	0.21402103	0.06849711	0.30398611	-0.0930743
SCN5A	-0.1246003	0.06609993	0.07860297	-0.0302755	0.35102498
SEPT4	-0.03662	0.343636	0.619768	0.089992	0.137388
SH3RF1	-0.1094209	0.18174533	0.38771998	0.68513105	0.03904216
SH3RF2	0.07390833	0.24630319	0.3562922	0.16468315	0.45167862
SH3RF3	-0.138367	0.14715163	0.11759756	0.1012961	0.63049572
SHARPIN	0.00021135	0.02626154	0.29447569	0.52119322	0.45052889
SHPRH	-0.0864314	0.15553551	0.32257155	0.48902269	0.11982506
SIAH1	0.11024875	0.12870396	0.52096596	0.60297963	0.81303292
SIAH2	0.2604934	0.19790578	0.44157513	0.45893578	1.27123042
SIRT1	-0.212556	0.18730049	-0.2576078	0.00659419	0.13700798
SIRT2	-0.2486877	0.12949564	-0.1405188	0.02716202	-0.2052684
SKI	-0.0644923	0.03026957	0.33113391	0.06796745	0.52000968
SKP1	0.05951172	0.40552789	0.09840381	0.82567885	0.51640832
SKP2	0.09307347	0.07873174	0.26946685	0.35971195	0.56022959
SLC22A18	0.086912	0.267447	0.356163	0.184872	1.10698
SMAD2	0.176615	0.176039	0.20688	0.357782	1.09447
SMAD3	0.003465	0.072341	0.137309	0.220335	0.481053
SMAD5	-0.17823	0.1754	-0.18077	0.213904	0.52753
SMAD6	-0.05536	0.127516	0.257099	-0.09137	0.504536
SMAD7	-0.00326	0.181098	0.402119	0.252225	0.175438
SMURF1	0.04310002	0.1699526	0.02898267	0.70264288	0.30399898
SMURF2	0.08288841	0.36831547	0.19397721	1.00616186	0.08503424
SNCAIP	-0.1693716	0.09414989	-0.0858109	0.19922057	0.24847841
SNX9	0.09746471	0.22917161	0.34604171	0.39623389	0.72849379
SOCS1	-0.099486	0.08335925	0.11355188	0.1328131	0.27005954
SOCS2	-0.0814123	0.13984662	0.47115371	0.10284855	0.21708668
SOCS3	-0.0580834	0.15908132	-0.0207293	0.11808241	0.41865344
SOCS4	-0.217344	0.07844099	-0.0254506	-0.0144859	0.23987595
SOCS5	-0.0817849	0.07386844	0.16552138	0.2077196	0.43539372
SOCS6	-0.0835914	0.13844349	0.51573915	-0.1127589	0.14624626
SOCS7	-0.203287	0.0445795	0.1260155	0.24073676	0.30945717
SPG20	-0.1148204	0.09407814	0.10386795	0.04336876	0.21370342
SPOP	-0.1609126	0.071266	0.04545887	0.15377534	0.13403145
SPOPL	-0.23122	0.18188166	-0.1362748	-0.0325137	0.42976852
SPRYD5	-0.1731243	0.2041415	0.2848852	0.02775511	0.09339605
SPSB1	0.13084234	0.14800783	0.08601347	0.65237754	0.52069847
SPSB2	-0.068854	0.18462462	0.48952139	0.09555086	0.40232092
SPSB3	1.22536009	0.23396401	1.6579091	1.69887436	1.72729329
SPSB4	-0.0613112	0.06113798	-0.1512092	0.45089105	0.85957207
SQSTM1	-0.1773269	0.17533446	-0.0043084	0.33881948	0.05879786
STUB1	-0.2031472	0.04716125	0.21335654	-0.0647894	-0.1113017
STX8	-0.2276809	0.13744094	-0.0560437	0.18474148	-0.1239883
SUGT1	-0.2068594	0.13453686	0.16677912	0.20310116	-0.0552677
SUMO1	-0.09526	0.18254575	0.52186191	0.21717271	0.06840828
SUMO2	-0.1344518	0.13707819	0.05519409	0.31950875	0.78328467
SUZ12	-0.1461887	0.0550644	0.17443425	0.09078136	0.41981449
SYTL4	-0.5133112	0.16949633	-0.3859351	-0.3439145	-0.0699707
SYVN1	-0.1349862	0.24104399	0.09583172	-0.0681917	-0.0242149

Gene symbol	Integrated intensity	SD Integrated intensity	Mean intensity replicate #1	Mean intensity replicate #2	Mean Intensity replicate #3
TANK	-0.2377573	0.1064623	-0.0481688	0.21744577	0.06066882
TBC1D7	-0.1253855	0.12988725	0.42975491	0.228969	0.13491016
TCEB1	0.72047487	0.04405397	0.96610756	1.23696367	1.64337885
TCEB2	0.739004	0.35460035	0.86567328	0.96367565	2.54255792
TCEB3B	-0.047998	0.05451772	0.27184778	0.37081476	0.52304311
TCEB3C	0.03756603	0.15618026	0.22009934	0.25096655	0.58886611
TCP1	0.4695794	0.11201262	0.53349611	0.88077848	1.11426991
TEX14	-0.1704708	0.06679963	0.04131491	0.10332255	0.00939629
TMBIM6	0.11728714	0.17945248	0.30463068	0.36810979	0.91753708
TMEM129	0.0901471	0.33860225	0.14064083	1.17358287	0.32051767
TMEM173	-0.1315049	0.14138906	-0.1547986	0.2500617	0.56487816
TMEM189	0.04617152	0.5499174	0.11119532	0.08007921	1.63511551
TNFAIP1	0.03598941	0.05533914	0.22313918	0.26404305	0.33820991
TNFAIP3	-0.0746396	0.261674	0.4041864	0.10989432	0.01354534
TNFRSF14	-0.2992377	0.12233323	0.00351867	0.05140684	-0.2023414
TNFRSF1B	-0.088647	0.10156243	0.26069626	0.22185101	0.61300137
TNIP1	-0.2450096	0.17372635	0.26633873	0.11645043	-0.1191671
TNIP2	-0.1213025	0.19814116	-0.009214	0.03777142	0.13198805
TNIP3	-0.2283933	0.18229192	0.04389947	0.52160465	-0.0140059
TNKS	-0.127589	0.0936367	0.20914894	0.23748142	0.22955855
TNKS2	-0.1802148	0.43470325	-0.2864881	0.55097239	-0.0475042
TOLLIP	-0.1923792	0.22228241	0.40879452	0.0385592	0.21236099
TOM1L1	0.07321954	0.34666559	0.70331851	0.19368569	0.02542617
TOP2A	0.08874573	0.33477369	0.03982677	0.13510092	0.79472222
TOPORS	-0.0675922	0.2038658	0.18096763	0.0848083	0.62731377
TPP2	0.19302106	0.42092024	0.79950038	0.32122571	0.65375149
TRAF1	-0.169182	0.123747	0.10518513	0.06487983	0.37236535
TRAF2	-0.3704612	0.30517968	-0.5516487	0.17085701	0.03630913
TRAF3	-0.0656589	0.22191791	0.21420407	-0.019189	-0.1270557
TRAF4	-0.0943778	0.2815339	-0.1561277	0.13000211	0.7284167
TRAF5	-0.0412152	0.31505688	0.15861785	0.17771296	0.03475636
TRAF6	-0.2002234	0.04776396	0.07584465	0.01504368	0.34097819
TRAF7	-0.1577921	0.17959871	0.06568393	0.44242528	0.08268982
TRAIP	-0.1461988	0.05327848	-0.0761749	0.18289175	0.25543349
TREX1	-0.2685975	0.16609141	0.0755318	0.05547417	-0.2735285
TRIB1	-0.1111177	0.16578119	0.44923401	0.19277267	0.02300185
TRIB2	0.2653508	0.16195492	0.57767382	0.54639547	0.68402263
TRIB3	0.13931999	0.3419064	0.17192168	1.35893877	0.20392345
TRIM10	-0.2525158	0.19677946	0.09279746	0.18814974	-0.08495
TRIM11	-0.1256907	0.11131163	-0.1024438	0.09547896	0.30118854
TRIM13	-0.2538926	0.12785019	-0.0163703	0.11087929	0.03105937
TRIM15	-0.1381113	0.32788941	0.10687627	0.31309446	-0.4492114
TRIM17	-0.1483291	0.56513789	0.65476276	-0.3959565	0.20025943
TRIM2	-0.1159352	0.08414028	0.21141019	0.04070225	0.22853362
TRIM21	-0.0207301	0.1593124	0.10540181	0.16817996	1.05844982
TRIM22	0.00220698	0.35514244	0.61471121	0.23103617	-0.1287439
TRIM23	-0.0563274	0.17680044	0.25777634	0.3672587	-0.012017
TRIM24	-0.1065297	0.32104	0.56501438	0.1911668	-0.1433627
TRIM25	0.1437916	0.2814339	0.30511769	0.05382571	0.83883632
TRIM26	0.0438176	0.28399224	0.32227982	-0.1020312	0.55788243
TRIM27	-0.1441664	0.16189237	-0.1470892	0.29948513	0.36962145
TRIM28	-0.2213065	0.04460922	0.03085481	-0.0305289	0.07996228
TRIM3	-0.2659931	0.07841906	-0.0982723	-0.0251541	-0.1282485
TRIM31	-0.1351689	0.50523734	0.49022299	0.45866172	-0.4243792
TRIM32	-0.2475377	0.31838449	0.26350028	0.07952378	-0.4524258
TRIM33	-0.2127006	0.05730927	0.26665212	-0.0939017	0.19152869
TRIM35	-0.2070228	0.20236156	0.11631632	0.24298341	-0.2293971
TRIM36	-0.2290559	0.16145854	0.15417766	0.19702352	-0.0609206
TRIM37	-0.17193	0.104598	0.046956	-0.06506	0.596533
TRIM38	-0.2672593	0.20822282	-0.1529031	-0.0865382	0.39000717
TRIM39	-0.1376507	0.30235421	0.21737646	0.77384235	-0.0578155
TRIM4	-0.0457194	0.04662189	0.18102462	0.00749669	0.30670896
TRIM40	-0.1742609	0.04944998	0.02224483	0.23358021	0.35871165
TRIM41	-0.1527733	0.25052841	0.23788706	-0.1880103	0.35575721
TRIM42	-0.1669484	0.15355881	0.24236646	0.13410544	0.19645073
TRIM43	0.10034556	0.23574377	0.39341264	0.80257445	0.31722404
TRIM44	-0.0776609	0.26584242	0.35784233	-0.0321919	0.55508806
TRIM45	-0.2057066	0.07158116	0.05014691	0.33954626	-0.0795354
TRIM46	-0.167117	0.34641444	-0.2068982	0.82606931	-0.0851087
TRIM47	-0.0735512	0.14651203	0.17074064	0.54404161	0.21003228
TRIM48	0.060106	0.6362428	-0.3913674	1.55125431	0.35678234
TRIM49	-0.3722199	0.10582778	-0.1042664	0.01421694	-0.1550343
TRIM5	0.0913684	0.34236154	0.17523571	0.98117424	-0.1033456
TRIM50	-0.197955	0.18145979	0.20254822	0.29826073	-0.0482923
TRIM52	-0.1848239	0.19702732	-0.2505953	0.28162545	0.21522187
TRIM54	-0.1247205	0.24267843	0.26922215	0.72023935	-0.1366426
TRIM55	-0.049242	0.09474929	0.35585024	0.25221839	0.42281898
TRIM56	-0.045471	0.12693934	0.17917136	0.25462677	0.42011308
TRIM58	-0.2159531	0.10255017	0.09527811	-0.1471038	0.28150699
TRIM59	-0.0391813	0.03812265	0.2047458	0.49142013	0.3801214
TRIM6	-0.1874752	0.38494414	0.15517844	-0.4713793	0.25234543
TRIM6-TRIM34	-0.1627504	0.29810092	-0.239337	0.0769116	0.73938675

Gene symbol	Integrated intensity	SD Integrated intensity	Mean intensity replicate #1	Mean intensity replicate #2	Mean Intensity replicate #3
TRIM60	-0.2328472	0.14992466	-0.0249433	-0.0412196	0.47423922
TRIM61	0.15806085	0.56947849	0.14087631	0.01279486	1.23913636
TRIM62	-0.0255092	0.33432165	0.37471444	-0.2265807	0.69619616
TRIM63	0.04547589	0.31734935	0.2538299	0.22464908	1.10743474
TRIM65	-0.1823318	0.10016682	-0.0225771	0.28710265	0.22370372
TRIM67	-0.2216426	0.19307906	0.22284735	-0.0847336	-0.0679002
TRIM68	0.12253974	0.42946705	1.06260771	0.26312065	-0.0695777
TRIM69	-0.0402458	0.19283901	-0.2362152	0.19888322	0.78587341
TRIM7	-0.1176843	0.27137978	-0.3588444	0.30433036	0.74644469
TRIM71	0.00834568	0.18886749	-0.0532259	0.18204208	0.59576175
TRIM72	0.27032094	0.37059756	0.17844613	1.36162495	0.47418711
TRIM73	0.0853172	0.53458487	1.15507843	0.10299628	0.51640487
TRIM74	-0.1800957	0.13015616	0.28236163	0.06212429	0.28358462
TRIM8	0.18608945	0.6425995	0.64468743	-0.1364845	0.68180887
TRIM9	0.07040745	0.22541532	0.27714353	0.1222079	0.58782562
TRIML1	-0.1873742	0.26589868	-0.3452519	0.54773689	0.40330296
TRIML2	0.0446496	0.22300251	0.40350224	0.25640108	0.32007199
TRIOBP	0.12573764	0.60726785	-0.0893602	0.08104945	1.09447755
TRIP12	0.26450929	0.11296638	0.5605126	0.54611454	0.67809841
TRPC4AP	-0.1728027	0.04551161	0.13863608	0.11349852	0.15192398
TSG101	-0.0946246	0.05601519	0.122983	0.12354114	0.54331749
TSPAN17	-0.2364473	0.12962878	0.0837384	0.22013828	-0.0982014
TSPYL5	-0.2493473	0.19299317	-0.0462644	-0.0989564	-0.01882
TTC3	-0.1296427	0.17617599	-0.115589	0.16002402	0.46316177
TTK	0.0125232	0.05262269	0.24439933	0.22742005	0.48128787
TUBA1B	-0.0547182	0.05828385	0.20200083	0.10655828	0.41589955
TUBB	0.15447074	0.22966242	0.33394857	0.34350039	0.55052058
TULP4	-0.0486431	0.33980843	0.06310259	0.79609098	-0.1213556
TXNIP	-0.015276	0.14526426	0.08264993	0.38195205	0.30970154
UBA1	-0.1922696	0.10257608	0.14859249	0.04896819	0.33050063
UBA2	0.06651225	0.18536015	0.72058032	0.13539587	0.58804919
UBA3	0.20723616	0.47267853	0.2580321	0.0832031	1.2801455
UBA5	-0.1118429	0.17425138	0.50139883	0.45192907	0.05527378
UBA52	0.1162799	0.2608738	0.6182278	0.14286805	0.79029057
UBA6	-0.2076182	0.24687076	-0.2836963	0.66069337	0.04947707
UBA7	-0.1553128	0.33281662	0.16769623	0.33225165	-0.2661995
UBAC1	-0.3068032	0.16421499	-0.1524336	0.21707346	-0.1636308
UBAP1	-0.2112112	0.16880673	0.18625523	0.12562583	-0.0475367
UBB	-0.1589872	0.11567232	0.35107985	0.09060243	0.0312942
UBC	0.91131933	0.99380665	0.17630769	1.23716596	2.50529487
UBD	0.14659976	0.21485475	0.51433387	0.20535099	0.98746636
UBE2A	-0.2091105	0.06751907	-0.0004284	0.05310404	0.25331411
UBE2B	0.13753827	0.41345672	0.78724413	0.33860173	0.10492216
UBE2C	-0.2240304	0.20374073	-0.4063582	0.09417855	0.50213088
UBE2D1	-0.1070144	0.27350482	0.11353288	-0.0221008	0.95612666
UBE2D2	-0.1116549	0.05625528	0.23400765	0.15177824	0.48551906
UBE2D3	-0.0918489	0.3538929	-0.011046	-0.2443617	0.93333578
UBE2D4	0.00200273	0.48366212	0.17620673	-0.1413907	0.93652252
UBE2E1	-0.2013295	0.06259916	-0.0684059	0.01210935	0.30098999
UBE2E2	0.1245791	0.26018195	0.79514055	0.06087896	0.58425662
UBE2E3	-0.2228893	0.05344564	-0.1986715	0.07068854	0.18474845
UBE2F	0.91889909	0.81050831	2.36612274	0.64416511	1.73668924
UBE2G1	0.03022199	0.29663028	0.86520083	-0.014464	0.5948692
UBE2G2	0.03053837	0.37612481	0.80702798	0.12285632	0.16562293
UBE2H	-0.1384963	0.08589677	0.04110318	-0.003445	0.43754737
UBE2HP	-0.0155334	0.23702571	0.2936559	0.11829547	0.50565007
UBE2I	-0.1296815	0.2026657	-0.2578504	0.45654959	0.63682552
UBE2J1	-0.1014304	0.10776145	0.06938343	0.16994216	0.30514835
UBE2J2	-0.3072025	0.07647425	-0.3136413	0.18887631	-0.375881
UBE2K	-0.2099214	0.05365549	0.25940745	-0.0267328	0.51206196
UBE2L3	-0.0843478	0.14453571	0.11081961	0.30369773	0.5582067
UBE2L6	-0.1635555	0.13839554	0.03997155	-0.1451469	0.19622306
UBE2M	-0.2064639	0.18651156	0.01916308	0.14033125	-0.2466518
UBE2N	-0.2271866	0.17624022	0.19688092	0.29419854	0.13518264
UBE2NL	-0.0407124	0.3380545	0.53243093	-0.1295076	0.79907026
UBE2O	-0.2569576	0.17311943	-0.1847839	0.52043602	0.18490941
UBE2Q1	-0.2195678	0.15153984	-0.235226	0.11514976	-0.0343018
UBE2Q2	-0.1325134	0.06974725	0.09670613	0.35837058	0.06352161
UBE2R2	-0.0419341	0.15863074	0.40810414	0.15709947	0.15991871
UBE2S	0.01440213	0.26475339	0.51124894	0.1980003	0.06434754
UBE2T	-0.0803354	0.26873655	0.44509168	-0.0242058	0.23510855
UBE2U	-0.3545245	0.22728188	-0.0939985	0.05475179	-0.5604236
UBE2V1	-0.3056113	0.09829299	-0.0612869	0.1041357	0.12472105
UBE2V2	-0.1359243	0.11664151	0.02002452	0.18074729	0.53822164
UBE2W	-0.1636918	0.16528577	0.13649341	0.44081848	-0.2389456
UBE2Z	-0.0311751	0.02832089	0.06280149	0.22232964	0.39355283
UBE3A	-0.3296472	0.03804037	-0.2568921	-0.1993787	-0.1097899
UBE3B	-0.1649284	0.20156281	0.26402181	-0.031771	0.08264352
UBE3C	-0.0882428	0.08604224	0.00139943	0.12370246	0.26112168
UBE3D	-0.0819751	0.0985173	0.06316895	0.09926475	0.2350979
UBE4A	-0.1386919	0.06697293	0.09252295	0.29802903	0.33422889
UBE4B	-0.1265904	0.09891709	0.05007136	0.23115135	-0.0960243

Gene symbol	Integrated intensity	SD Integrated intensity	Mean intensity replicate #1	Mean intensity replicate #2	Mean Intensity replicate #3
UBOX5	-0.0817879	0.31282623	0.29874263	0.15982897	-0.0720296
UBQLN1	-0.2070137	0.18104574	0.32180837	-0.1954143	0.29870858
UBQLN4	-0.1036695	0.06176109	-0.0930464	0.1368966	0.09341017
UBR1	-0.310188	0.19295141	0.11247863	0.26425386	-0.2816995
UBR2	-0.124499	0.06282972	0.35052459	0.04970479	0.3385394
UBR3	-0.3134407	0.04788071	-0.0896684	-0.0616791	-0.0049492
UBR4	0.1143318	0.07955161	0.6003841	0.24503217	0.47357586
UBR5	0.34853749	0.5273589	0.82941272	0.11523453	1.20601201
UBR7	-0.129995	0.12975733	0.17024461	0.39427628	0.08087687
UBXN1	0.00170969	0.06708021	0.30255199	0.29811634	0.55134236
UBXN11	0.20562419	0.59719973	0.5952462	1.1463931	-0.2744408
UBXN2A	-0.096741	0.34328195	-0.3987577	0.06465376	0.68367216
UBXN2B	-0.2418865	0.18983578	0.10746699	-0.0633402	0.13521912
UBXN7	-0.2259567	0.01412756	0.09989621	-0.0352852	0.22984188
UEVLD	-0.2738673	0.3154859	0.46157959	-0.3247014	-0.3173012
UFC1	-0.1606607	0.25187622	0.12237912	0.73771069	0.0197219
UFD1L	-0.1886849	0.05672623	0.18685971	0.10337055	0.1708081
UFM1	-0.1340965	0.09877397	-0.0895463	0.28114034	0.32713692
UHRF1	-0.1723933	0.06554926	-0.0347124	0.35463026	0.36725433
UHRF2	-0.2431949	0.08397897	-0.1377342	-0.1148181	0.47971399
UNKL	0.05553208	0.24898559	0.77221637	0.18892593	0.45820592
USP27X	0.00116998	0.23445427	0.15284255	0.34420467	0.42630541
VCP	-0.1024425	0.09401804	0.1886861	-0.1202315	0.50855695
VHL	-0.0084872	0.30565606	0.19426014	0.00747097	0.86244466
VHLL	-0.4521536	0.19120582	-0.2634288	-0.368539	0.0742603
VPRBP	0.09522049	0.04785501	0.49728338	0.31402982	0.58277428
VPS24	-0.0818907	0.05770361	0.18289826	0.50011404	0.23314917
VPS28	-0.1583918	0.32396601	0.41430279	-0.0490273	-0.012237
VPS36	-0.2694151	0.38041218	0.01997623	-0.3376928	0.61211178
VPS41	-0.134932	0.15092922	0.25908079	0.08216327	0.19402245
VPS8	-0.1607572	0.14555922	0.22792665	0.13617878	0.00661583
WAC	0.10115528	0.32147906	0.37994555	0.89489724	0.31233906
WASH1	-0.0365949	0.05006903	0.29002901	0.35625822	0.41179392
WDR21A	-0.1127904	0.00905712	0.23014652	0.01854536	0.44641645
WDR22	-0.2115852	0.01294598	0.18783971	0.12317661	0.16305484
WDR23	-0.2183999	0.03643095	-0.04746	0.30732678	0.13372781
WDR40A	-0.1582087	0.12565671	-0.1221754	0.07190919	0.58247895
WDR42A	-0.2119985	0.29808612	0.40534144	-0.1663553	0.0429731
WDSOF1	-0.1947814	0.25411847	0.43434091	-0.0819714	0.14063825
WDSUB1	-0.1310032	0.15925538	0.31863956	-0.0475056	0.20815637
WDTC1	-0.1708186	0.3812985	0.47432268	-0.0862303	-0.4114281
WFS1	-0.2561591	0.12690624	0.12511907	-0.1110511	0.43699515
WHSC1	-0.1079282	0.12647769	-0.0706751	0.16425107	0.1497975
WHSC1L1	-0.1010666	0.31984174	-0.2261004	0.92783873	0.5107952
WSB1	-0.2440836	0.11162884	0.21399405	0.04876113	-0.019853
WSB2	-0.2191451	0.18500929	-0.1527694	0.03001426	0.3790044
WWP1	-0.3177664	0.19565735	0.14454766	-0.0997997	-0.1302387
WWP2	-0.1248723	0.09358873	-0.0148089	0.25607735	0.23267122
WWTR1	-0.1241345	0.1694637	0.1664182	0.12399754	0.18421863
XBP1	-0.146238	0.28445198	0.00447548	-0.1802578	0.68626057
XIAP	-0.2251444	0.12080756	0.27376688	0.07292075	0.0986212
XRCC5	-0.2084053	0.11032626	-0.0165293	0.17190092	-0.1653406
ZBTB16	-0.0130223	0.30439866	0.6747773	-0.1744033	0.60282154
ZC3HC1	-0.2765377	0.14975352	0.2940226	0.0812447	0.19787443
ZER1	-0.1252963	0.21194986	0.10224835	-0.2036971	-0.2614415
ZFAND5	0.05848981	0.393357	0.01683463	0.62230352	0.32299809
ZFAND6	0.16387492	0.59069488	0.92429532	0.06906248	0.41165518
ZFP91	-0.2071655	0.13902728	0.18774	0.14197768	0.11028661
ZMYM2	-0.1399477	0.08831611	-0.0890664	0.20586049	0.4796786
ZMYND11	-0.0858105	0.40495006	0.61703531	0.21909264	-0.185506
ZNF207	0.31861131	0.41149256	0.07512575	0.21654347	1.08632103
ZNF645	-0.3303754	0.14669443	-0.085863	0.03872981	0.15370035
ZNF675	-0.1076632	0.32771595	0.60046575	-0.1512149	0.59653722
ZNF746	-0.0779804	0.15707158	0.13349761	0.53263418	0.02205732
ZNRF1	-0.1133409	0.33968127	0.41055695	0.16634032	0.099331
ZNRF2	-0.0062708	0.05962581	0.29660522	0.49763338	0.60109856
ZNRF3	-0.1799237	0.11075982	-0.1625521	0.57050541	0.15075438
ZNRF4	-0.1996004	0.00882311	-0.0733696	-0.0623855	0.22528968
ZRANB3	-0.1276479	0.28314164	0.06808022	0.06293066	0.40726955
ZSWIM2	-0.0228019	0.31354815	-0.09969	0.03874176	0.85595204
ZYG11A	-0.1449054	0.3289971	0.24582849	-0.011469	-0.0304274
ZYG11B	-0.073264	0.00887275	0.1935639	0.3063033	0.36082817
